# Targeting ERBB3 and AKT to overcome adaptive resistance in EML4-ALK-driven non-small cell lung cancer

**DOI:** 10.1038/s41419-024-07272-7

**Published:** 2024-12-18

**Authors:** Josephina Sampson, Hyun-min Ju, Nan Zhang, Sharon Yeoh, Jene Choi, Richard Bayliss

**Affiliations:** 1 Astbury Centre for Structural Molecular Biology and School of Molecular and Cellular Biology, Faculty of Biological Sciences, University of Leeds, Leeds, LS2 9JT UK; 2 Department of Pathology, Asan Medical Center, University of Ulsan College of Medicine, Seoul, 138-736 Korea

**Keywords:** Growth factor signalling, Non-small-cell lung cancer

## Abstract

The fusion event between EML4 and ALK drives a significant oncogenic activity in 5% of non-small cell lung cancer (NSCLC). Even though potent ALK-tyrosine kinase inhibitors (ALK-TKIs) are successfully used for the treatment of EML4-ALK-positive NSCLC patients, a subset of those patients eventually acquire resistance during their therapy. Here, we investigate the kinase responses in EML4-ALK V1 and V3-harbouring NSCLC cancer cells after acute inhibition with ALK TKI, lorlatinib (LOR). Using phosphopeptide chip array and upstream kinase prediction analysis, we identified a group of phosphorylated tyrosine peptides including ERBB and AKT proteins that are upregulated upon ALK-TKI treatment in EML4-ALK-positive NSCLC cell lines. Dual inhibition of ALK and ERBB receptors or AKT disrupts RAS/MAPK and AKT/PI3K signalling pathways, and enhances apoptosis in EML4-ALK + NSCLC cancer cells. Heregulin, an ERBB3 ligand, differentially modulates the sensitivity of EML4-ALK cell lines to ALK inhibitors. We found that EML4-ALK cells made resistant to LOR are sensitive to inhibition of ERBB and AKT. These findings emphasize the important roles of AKT and ERBB3 to regulate signalling after acute LOR treatment, identifying them as potential targets that may be beneficial to prevent adaptive resistance to EML4-ALK-targeted therapies in NSCLC.

## Introduction

Targeting driver oncogenes in non-small cell lung cancer (NSCLC) has revolutionized the clinical outcomes of those patients. One of the most successful examples in lung cancer survival is seen in NSCLC patients harbouring the oncogenic fusion between echinoderm microtubule-associated protein-like 4 (EML4) and anaplastic lymphoma kinase (ALK) [1]. EML4-ALK oncogenic fusion identified in 2007 and is present in 5% of patients with NSCLC [1]. Up to 15 different variants of EML4-ALK have been discovered so far with most of them having the ALK gene breakpoint at exon 20, but various breakpoints in the EML4 gene. The most frequent EML4-ALK variants commonly found in NSCLC patients are variant 1 (V1, 43%) and variant 3 (V3, 40%) [2]. EML4 is a microtubule-associated protein that contributes to cytoskeleton stability and chromosome congression during mitosis [3, 4]. ALK is a receptor tyrosine kinase (RTK) that activates mitogenic signals through MAPK, JAK/STAT, and PI3K/AKT signalling pathways [5–7].

Since the discovery of EML4-ALK oncogenic fusion, several small molecule ALK inhibitors have been developed and approved for the treatment of ALK-positive NSCLC patients including, crizotinib (1st generation), ceritinib, alectinib, and brigatinib (2nd generation), and lorlatinib (LOR, 3rd generation) [8]. Despite the progress on the development of potent ALK inhibitors, ALK-resistant mutations such as G1202R, F1174L, L1196M, G1202R/L1196M and G1202R/S1206Y, also known as on-target resistance, arise after ALK-tyrosine kinase inhibition (TKI) treatments with either crizotinib, ceritinib or alectinib [8]. In addition to on-target drug-resistance mutations, there are several reports suggesting the involvement of (i) alternative tyrosine kinase receptors, including EGFR, KIT, and SRC, (ii) mutations in key genes such as *p53*, *PI3KCA*, *YAP,* and *MET*, and (iii) activation of key proliferation and anti-apoptotic pathways [8, 9]. These are known as off-target resistance mechanisms that further complicate the development of systematic therapies for EML4-ALK-positive NSCLC patients.

EML4-ALK oncogenic fusion protein activates several downstream signaling pathways that are also controlled by other RTKs, such as ERBB (HER) family signalling [10]. Thus, upregulation or activation of RTK receptors, such as EGFR, trigger bypass signalling pathways that drive signalling in the presence of ALK-TKIs. One such example is the activation of signal transduction pathways through EGFR, ERBB2, and ERBB3 in ALK-TKIs-resistant EML4-ALK cell lines, in which the combination of erlotinib (ERL) or afatinib and crizotinib significantly affected cell survival [10–12].

Key driver oncoproteins such as EML4-ALK stimulate signalling pathways and evade cell death. Targeting these driver oncoproteins gives remarkable results in therapy, but often leads to drug resistance. One such mechanism of drug resistance is the loss of negative feedback pathways, also known as an adaptive response, that are essential in controlling the duration of signalling pathways [13]. Thus, inhibition of driver oncoproteins can paradoxically increase tyrosine phosphorylation either by increased autocatalytic activity or from loss of negative feedback responses [13]. Treatment of EML4-ALK-positive NSCLC patients with potent ALK inhibitors has been beneficial for their survival, however little is known about the adaptive responses from ALK inhibition. LOR has been approved as a first-line treatment for untreated EML4-ALK-positive NSCLC patients and as a second-line treatment for previously ALK-inhibitor-treated EML4-ALK-positive NSCLC patients [14, 15]. However, on-target resistant mutations, including F1174L/G1202R, G1202R/L1196M, L1196M/D1203M and G1202R/S1206Y, in the ALK domain have been recently reported after LOR treatment [16, 17]. Despite the recent progress in the field of ALK-TKIs, little is known about their molecular mechanisms and adaptive responses.

In the present study, we explored the vulnerability and adaptive responses of EML4-ALK-harbouring NSCLC cell lines upon acute ALK inhibition with LOR, the latest clinically approved ALK-TKI inhibitor. Using a peptide chip array to measure kinase activity, we observed increased tyrosine phosphorylation in several proteins, including the ERBB receptor family and AKT1 in LOR-treated EML4-ALK-harbouring cell lines, H3122 (V1) and H2228 (V3). We then probed the synergistic effect of ALK and ERBB loss either by chemical inhibition or knockdown in NSCLC cell lines, which led to a significant reduction of cell proliferation and survival in EML4-ALK + NSCLC cell lines only. We found that ERBB3 activity had a profound impact on EML4-ALK-driven cell lines and preferentially in H2228 cells. We further investigated the synergistic effect of ALK and AKT inhibition when used in combination with LOR to overcome adaptive resistance in EML4-ALK-harboured cell lines. We found that LOR-resistant H2228 cells were sensitive to inhibition of ERBB with ERL or sapitinib (SAP) and AKT with AKT VIII compound. Taken together, we identify candidate proteins that control key signalling pathways in NSCLC upon acute ALK inhibition that could be potentially targeted in combination with current ALK-TKI inhibitors.

## Results

### Kinase activity profiling revealed increased tyrosine phosphorylation after LOR treatment

**Fig. 1:**
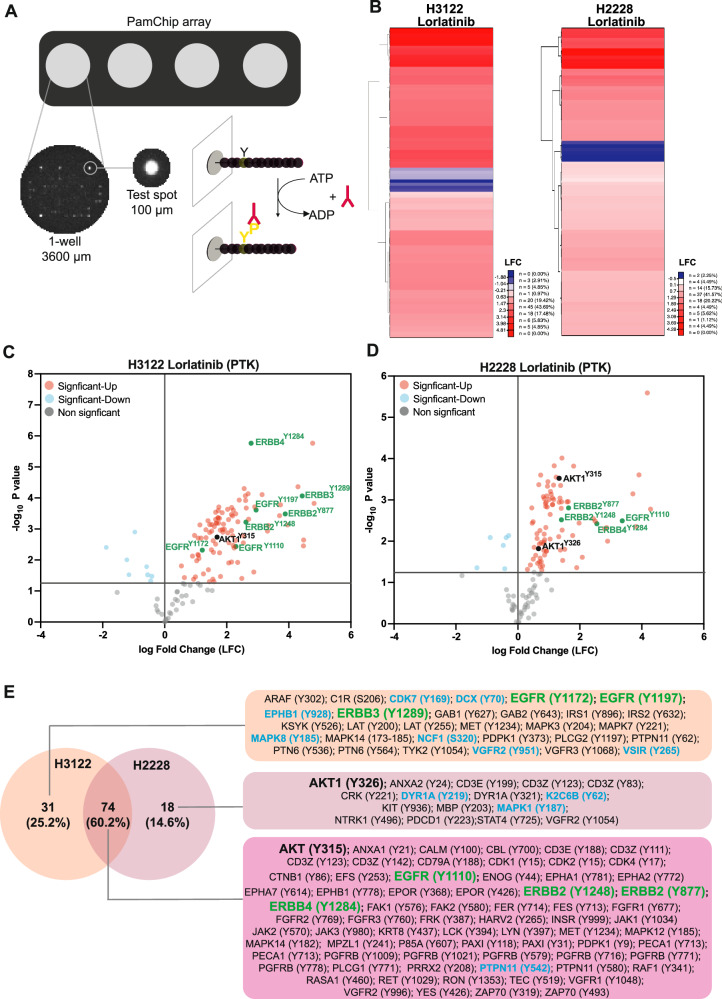
Analysis of phosphorylated tyrosines in LOR-treated EML4-ALK NSCLC cell lines using peptide chip array. **A** Schematic model showing the PTK PamChip peptide microarray system and experimental design. **B** Heat map of up-and down-regulated Tyrosine (PTK assay) bait peptide phosphorylation of lorlatinib (4 h) versus DMSO treatment by peptide chip array (*n* = 3 biological replicates) in H3122 and H2228 cell lines. Significant peptides (*p* < 0.05) were clustered using hierarchical order and Euclidean/ward algorithm. **C**, **D** Volcano plots of the bait peptide phosphorylation from PTK chip arrays in A showing significant up- (Red) and downregulation (blue) of peptide phosphorylation as defined by ANOVA and post-hoc Dunnett’s test versus DMSO treatment (*p* < 0.05). Non-significant peptides were highlighted in grey. Downregulated peptides were highlighted in light blue. ERBB family and AKT1 phosphopeptides were highlighted in green and black bold colours, respectively. **E** Venn diagram was generated to compare the phosphorylated Tyr peptides (up- and downregulated) after a 4 h treatment with lorlatinib in H3122 and H2228 cell lines. Tables of the specific phosphorylated Tyr sites and the associated proteins are shown. The down-regulated proteins were highlighted in cyan colour. The upregulated proteins selected for further analysis were highlighted in bold with green and black colours.

To identify adaptive response mechanisms, we examined the landscape of tyrosine phosphorylation upon acute LOR treatment in EML4-ALK-positive NSCLC cell lines using PamGene microarray peptide assay, known as protein tyrosine kinase (PTK) (Fig. 1A). LOR-treated lysates from cell lines harbouring EML4-ALK variant 1 (H3122) and variant 3 (H2228) were incubated on a porous microarray of 186 kinase substrates and tyrosine phosphorylation was measured by using a fluorescently labelled anti-phosphotyrosine antibody in the presence of ATP activity (Fig. 1A). The log2fold (LFC) signal intensities of each tyrosine bait peptide were calculated against DMSO treatment, clustered using the Ward/Euclidean analysis and represented as a heat map in each cell line (Fig. 1B; Supplementary Fig. S1; Data file S1). [18]. In the PTK array of LOR-treated H3122 cell line, we observed reduction of phosphorylation in a subset of tyrosine peptides that were associated with adaptor proteins including PTPN11^Y542^, kinases including mitogen-activated protein kinases (MAPK8), cyclin-dependent kinase 7 (CDK7), ephrin receptor (EPHB1) members receptors and vascular endothelial growth factor receptor (VGFR2) (Fig. 1B; Supplementary Fig. S1A, C). In the LOR-treated H2228 cell line, however, we identified a slightly different set of downregulated phosphotyrosine peptides including adaptor protein PTPN11^Y542^, kinases such as dual specificity tyrosine-phosphorylation-regulated kinase 1 A (DYRK1A)^Y312^ and MAPK1^Y187^ (Fig. 1B; Supplementary Fig. S1B, D).

Surprisingly, the overall tyrosine phosphorylation levels of proteins were significantly increased upon acute LOR treatment in both H3122 and H2228 cell lines using PTK peptide array (Fig. 1B; Supplementary Fig. S1A, B). In the PTK array of H3122, we found a unique pattern of significantly upregulated phospho proteins including non-RTKs such as ERBB family members, tyrosine kinase Yes (YES1), lymphocyte specific protein tyrosine kinase (LCK), LYN and MET, and several adaptor proteins including PTPN11^Y62^, PTN6^Y536^, PTN6^Y564^, IRS1^Y896^, IRS2^Y632^, GAB1^Y627^ and GAB2^Y643^ (Fig. 1C, E; Supplementary Fig. S1A; Data file S1). A different set of tyrosine peptides were significantly increased in LOR-treated H2228 cells, which were associated with increased phosphorylation of kinases such as AKT1^Y326^, KIT^Y936^, VGFR2^Y1054^, cell adhesion and migration CRK^Y221^, and glycoproteins involved in T-cell receptor activity including T-cell surface glycoprotein CD3 epsilon chain (CD3E) and CD3 zeta chain (CD3Z) (Fig. 1B–E; Supplementary Fig. S1B).

A significant upregulation of tyrosine phosphorylated peptides was identified in several adaptor proteins, non-receptor and receptor kinases. Among those phosphotyrosine peptides examined in both EML4-ALK + NSCLC cell lines, we identified a group of highly upregulated tyrosine kinases that belong to ERBB family members (EGFR^Y1110^, ERBB2^Y1248^, ERBB2^Y877^ and ERBB4^Y1284^), insulin receptor (INSR^Y999^), kinases such as AKT^Y315^, focal adhesion kinases including FAK1^Y576^ and FAK2^Y580^, Janus Kinases (JAK1^Y1034^, JAK2^Y570^, JAK3^Y980^), T-cell signalling kinase ZAP-70^Y319^ and ZAP70^Y493^, and several adaptor proteins including PTPN11^Y580^, P85A^Y607^ (PIK3R1), and EFS^Y253^ (Fig. 1C–E). In summary, a significant number of tyrosine peptides of several receptor, non-receptor kinases, T-cell signalling, and adaptor proteins were revealed to be highly phosphorylated after acute LOR treatment in both EML4-ALK + NSCLC cell lines.

### Phosphopeptide analysis identifies ALK-driven phosphorylation of ERBB receptor family and AKT1 after LOR treatment

**Fig. 2:**
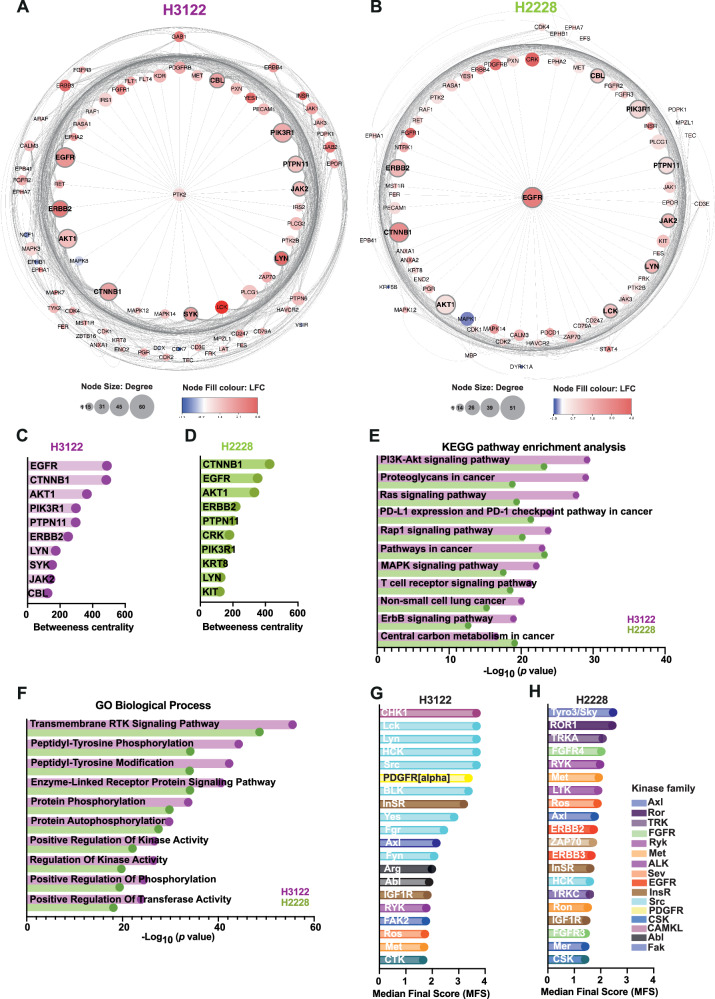
PTK peptide array identifies key kinase hubs upon LOR treatment in H3122 and H2228 cell lines. **A**, **B** Protein interaction network generated based on STRING database and visualized by Cytoscape. Networks were generated based on the LFC values of significant phosphotyrosine proteins identified in lorlatinib-treated H3122 and H2228 cell lines. In the schematic, node “hub” size indicates the number of connections (edges) to other proteins (nodes). Edges represent the events of phosphorylation, dephosphorylation, or binding between two proteins. The node fill colour defined the log2 fold-change (LFC) values of proteins (nodes) either up- (red) or downregulated (blue) after lorlatinib treatment in H3122 and H2228 cell lines. The top 10 most highly activated proteins from each network are highlighted in the grey circles. **C**, **D** Bar plot summarizing the top 10 proteins from A and B using betweeness centrality algorithm in Cytoscape plugin cytoHubba. Analysis was generated based on the set of phosphotyrosine kinases from PTK assay data after 4 h treatment with lorlatinib in H3122 (magenta) and H2228 (light green). **E** KEGG pathway enrichment analysis (Fisher’s exact test) for upregulated phosphotyrosine proteins in response to lorlatinib treatment in H3122 (magenta) and H2228 (light green). **F** GO terms for biological processes of significantly upregulated phosphorylated tyrosine proteins from Data file S2. **G**, **H** Upstream Kinase Activity (UKA) of predicted kinases was scored based on significantly upregulated phosphorylation of tyrosine peptides. This was presented as the median final score (MFS) quantification from upstream kinase analysis (UKA) algorithm. Predicted kinases were ordered by the sum of kinase activity and specificity. The top twenty kinases with a high MFS have a higher probability of being differentially active after acute lorlatinib treatment.

To identify the signalling hubs implicated after LOR treatment in H3122 and H2228 cell lines, we generated functional network maps using STRING database in Cytoscape. The phosphotyrosine interactome of 104 significantly up- or downregulated phosphorylated peptides over the DMSO treatment in H3122 cell line revealed a highly regulated network of proteins including EGFR, ERBB2, and AKT1, that play a central role in the activation of several signalling pathways (Fig. 2A). A similar network was found in the 90 tyrosine phosphorylated proteins identified in the PTK assay of LOR-treated H2228 cells (Fig. 2B). The betweenness centrality was calculated for each protein from each network using cytoHubba plugin in Cytoscape, from which we identified the top 10 most important proteins (nodes) controlling the information flow in signalling pathways of H3122 and H2228 cell lines (Fig. 2C, D; Supplementary Fig. S2A, B). We identified EGFR, catenin beta-1 (CTNNB1), AKT1, PIK3R1, adaptor protein PTPN11, ERBB2, tyrosine protein kinase (LYN), non-receptor tyrosine kinase (SYK), Janus kinase (JAK2) and an E3-ubiquitin-protein ligase (CBL) among the most active proteins after LOR treatment in H3122 cells (Fig. 2C; Supplementary Fig. S2A). In LOR-treated H2228 cells, however, we scored the activity of several other proteins, including adapter molecule CRK, keratin-type II cytoskeletal 8 (KRT8/K2C8), and tyrosine protein kinase (KIT) (Fig. 2D; Supplementary Fig. S2B). Furthermore, we calculated the degree centrality, which measures the nodes (proteins) with a high degree (hubs) within the signalling network (Supplementary Fig. S3A, C). In both networks, the degree measurement scored EGFR, AKT1, PIK3R1, CTNNB1, and ERBB2 among the top five most activated proteins after LOR treatment (Supplementary Fig. S3A–D). We performed Kyoto Encyclopedia of Genes and Genomes (KEGG) pathway enrichment analysis to identify the key pathways that are upregulated after ALK inhibition with LOR. In agreement with the measurements found by cytoHubba analysis (betweenness and degree centrality), the KEGG enrichment analysis identified PI3K-AKT signalling pathway as the most prominent pathway activated in both LOR-treated cell lines (Fig. 2E). In addition, proteins with high levels of tyrosine phosphorylation were significantly associated with Ras signalling pathways, activation of MAPK and ERBB signalling pathways, activation of cell adhesion and motility pathway by Rap1 (small GTPase protein), regulation of adaptive immune responses by PD-L1 expression and PD-1 checkpoint and T-cell receptor signalling pathways and as expected, signalling pathways implicated in NSCLC network (Fig. 2E). Similar to the KEGG analysis, the majority of molecular activities from the upregulated phosphorylated proteins are found in transmembrane RTK signal transduction pathway and tyrosine phosphorylation/modification events in both EML4-ALK+positive cell lines, as scored by using Gene Ontology (GO) term enrichment analysis (Fig. 2F). Based on the significantly phosphorylated peptides identified from PTK peptide array, we predicted upstream kinase activity using a group-based prediction system from PamGene, known as Upstream Kinase analysis (UKA). Kinase score (median final score (MFS)) was calculated based on the sum of the significance and the kinase specificity. We identified twenty kinases with the highest MFS and a higher probability of being differentially active between LOR and DMSO condition in H3122 and H2228 cell lines (Fig. 2G, H). Unexpectedly, we observed a different subset group of kinases upregulated in each cell line. Among the most active kinases, we scored a number of kinases that belong to the Src family including tyrosine-protein kinase HCK, Lck, Lyn, BLK, Yes, Fgr, Fyn, and proto-oncogene tyrosine kinase Src after LOR treatment in H3122 cell line (Fig. 2G). In addition, we predicted high activity in a serine/threonine-protein kinase CHK1, cytoskeletal kinase such as FAK2, tyrosine kinases Ros and Met in LOR-treated H3122 cells (Fig. 2G). In the H2228 cell line, however, the kinase predictions scored a variety of kinases that can be upregulated after LOR treatment (Fig. 2H). Among the predicted kinases, found exclusively in LOR-treated H2228, we scored kinases that belong to the EGFR family including HER2 (ERBB2) and HER3 (ERBB3), fibroblast growth factor receptor kinases such as FGFR3 and FGFR4, immune response kinase ZAP70 and neuronal receptor tyrosin kinases including TRKA and TRKC (Fig. 2H). Several predicted kinases, including insulin-mediated kinases (InSR and IGF1R) and tyrosine kinases Met and Ros were found in both EML4-ALK+positive NSCLC cell lines (Fig. 2G, H). Complementary analysis by proteomap visualisation tools highlighted RAS, MAPK, and ERBB signalling pathways as the major drivers of signal transduction (cyan colour) in LOR-treated H3122 and H2228 cell lines (Supplementary Fig. S4A–D). These findings demonstrate a strong dependency on RTK signalling pathways, ERBB, MAPK and PI3K/AKT, in which EML4-ALK+positive NSCLC cells adapt to overcome ALK inhibition.

### Dual inhibition of ERBB receptors and ALK kinase exhibits anti-proliferative effect in EML4-ALK-harbouring NSCLC cell lines

**Fig. 3:**
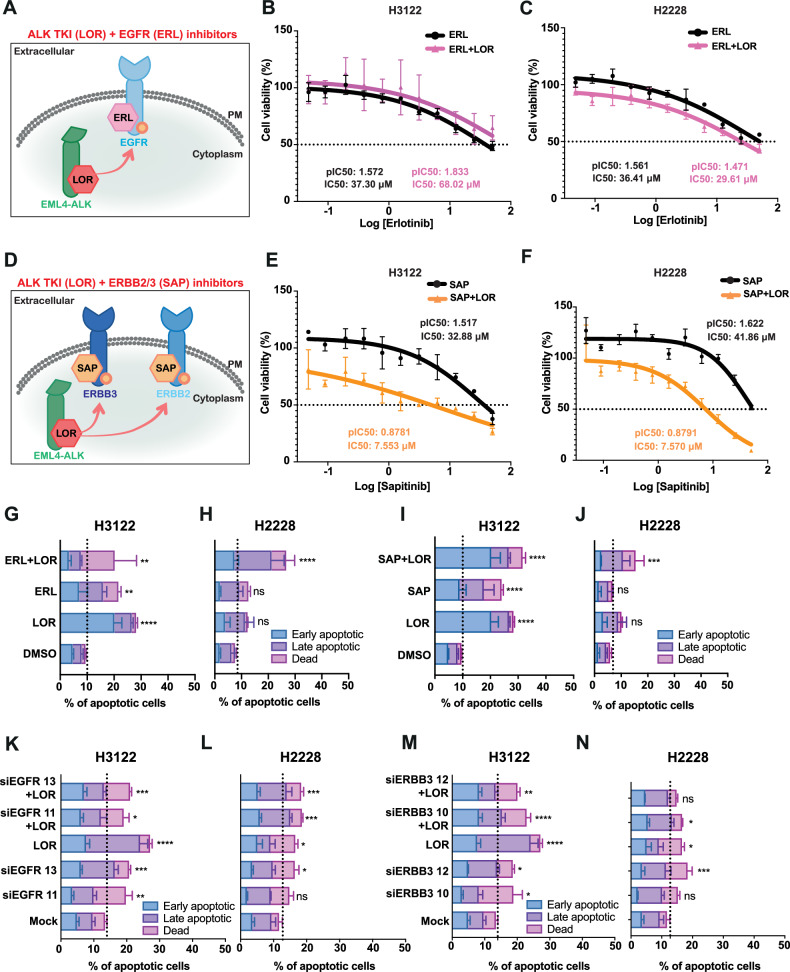
Dual inhibition of ERBB and ALK kinases enhances the anti-proliferative effect in EML4-ALK+positive NSCLC cell lines. **A** Model of the combination of ALK TKI (Lorlatinib-LOR) and EGFR (Erlotinib-ERL) treatment targeting EML4-ALK fusion cytoplasmic protein and up-regulated phospho EGFR receptor on the membrane, respectively. Red arrow indicates the upregulation of phospho EGFR after 4 h lorlatinib treatment. **B**, **C** EML4-ALK-positive NSCLC cell lines, H3122 (V1) and H2228 (V3), were treated with increasing doses of ERL and with or without LOR (3.12 nM) for 72 h. Cell viability was determined using CellTiter-Glo assays. The IC_50_ and pIC_50_ values were calculated using Prism 10.0 software. Data represent the mean of four independent biological replicates in each column; the bars denote ±SD. *p < 0.05, ****p < 0.0001 in comparison to ERL + LOR combination in H3122 and H2228 by two-way ANOVA (Sidak’s multiple comparisons test). **D** Model of the combination of ALK TKI (Lorlatinib-LOR) and ERBB2/3 (SAP) treatment targeting EML4-ALK fusion cytoplasmic protein and up-regulated phospho ERBB2/ERBB3 receptor on the membrane, respectively. Red arrows indicate the upregulation of phospho ERBB2 and ERBB3 receptors after 4 h lorlatinib treatment. **E**, **F** H3122, and H2228 cells were treated with increasing doses of SAP ± LOR (3.12 nM) for 72 h. Cell viability was determined using CellTiter-Glo assays. The IC_50_ and pIC_50_ values were calculated using Prism 10.0 software. Data represent the mean of four biological replicates in each column; the bars denote ±SD. H3122 and H2228 cells were treated with: **G**, **H** either LOR (100 nM), ERL (5 μM) or in combination; **I**, **J** either LOR (100 nM), SAP (200 nM) or in combination for 48 h before analysis by annexin V-based flow cytometry. **K**–**N** H3122 and H2228 cells were mock-depleted or depleted with siRNAs against EGFR or ERBB3 and in combination with LOR (100 nM) for 48 h before analysis by annexin V-based flow cytometry. Histograms represented the percentage of cells in apoptosis and were classified as early apoptotic, late apoptotic, and dead. Data represent the mean of four independent biological replicates; the bars denote ±SD. *p < 0.05, **p < 0.01, ***p < 0.001, ****p < 0.0001 in comparison to mock by two-way ANOVA.

Evidence from the peptide chip array and enrichment analysis pointed towards the activation of ERBB family members and specifically, EGFR, ERBB2, and ERBB3 as central players in controlling signal transduction (ERBB and MAPK signalling pathways) after LOR treatment in EML4-ALK+ positive NSCLC cell lines (H3122 and H2228) (Fig. 3A, D; Supplementary Fig. S4A–D). Because ERBB family members were one of the most upregulated class of proteins found in our peptide assay, we determined how the tyrosine kinase inhibitors (TKIs), ERL and SAP against EGFR and ERBB2/ERBB3, respectively, affect EML4-ALK-positive NSCLC cell lines (Fig. 3A, D) (Table 1). EGFR inhibition by ERL, either as a single agent or in combination with ALK inhibition (LOR) weakly reduced cell viability in both H3122 and H2228 cell lines (Fig. 3B). H2228 cells demonstrated higher sensitivity to the combination of ERL and LOR with a statistically significant twofold lower IC_50_ than in H3122 cells (Fig. 3C; Supplementary Fig. S4E, F). Overall, the ERL treatment alone did not significantly reduce cell viability. Similar to ERL monotherapy, a single treatment by SAP weakly inhibited cell viability, however, the combination treatment with LOR led to a statistically significant reduction of cell viability in both H3122 and H2228 cell lines (Fig. 3E, F; Supplementary Fig. S4E, F). In contrast, the combinations of either ERL or SAP and LOR did not significantly affect cell viability of BEAS2B, a non-cancerous bronchial epithelium diploid epithelial cell line (Supplementary Fig. S5A, B). No difference in cell viability of A549 cells, a KRAS G12S + NSCLC cell line, was observed upon ERL, SAP, and LOR treatments (Supplementary Fig. S5C, D). Hence, the differential cell survival observed between ALK+ and ALK- cell lines upon combination drug treatment is dependent on ERBB and ALK activities which are critical only in the EML4-ALK-driven NSCLC cell lines.

**Table 1:** Compounds used and concentrations.

Compound	Supplier	Final concentration (µM)
Lorlatinib (PF-06463922)	Selleck Chemicals	0.0312 or 0.1
Erlotinib	Selleck Chemicals	5
AKT VIII	Selleck Chemicals	1
Sapitinib	Selleck Chemicals	0.2

Dual treatment of the EML4-ALK + NSCLC cell lines with LOR and either ERL or SAP significantly increased the percentage of apoptotic cells (Fig. 3G–J; Supplementary Fig. S6A, B). Interestingly, monotherapy with LOR, ERL, or SAP led to a significant increase of apoptotic cell death in H3122, but not in H2228 cells (Fig. 3G–J; Supplementary Fig. S6A, B). In contrast, the single and combination treatments with ERL or SAP and LOR compounds, apart from the combination of SAP/LOR, did not significantly affect cell viability of A549 cells (Supplementary Fig. S6C–E). To complement the chemical inhibition against ERBB proteins, we performed a knockdown of EGFR and ERBB3 in combination with ALK inhibition in EML4-ALK-driven cell lines. Depletion of EGFR and ERBB3 alone or in combination with LOR led to a significant increase of cell death in both H3122 and H2228 cell lines (Fig. 3K–N, Supplementary Fig. S6F–I). While LOR alone had a minimal impact on cell death in H2228 cells, the combination with EGFR depletion led to greater cell death in those cells, recapitulating the effect we observed with the chemical inhibition of EGFR (Fig. 3H, L). We, therefore, concluded that ERBB and ALK activities are essential for the survival of EML4-ALK+driven NSCLC cell lines.

**Fig. 4:**
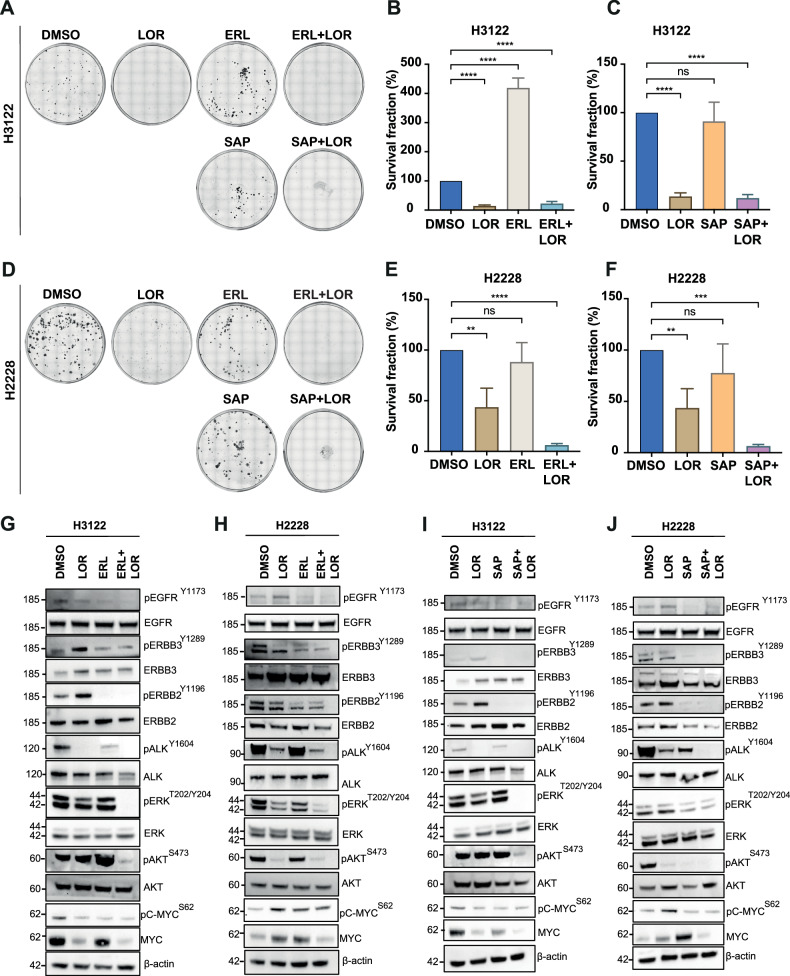
Loss of ERBB activities and cell proliferation signaling pathways upon dual inhibition with ERBB and ALK in EML4-ALK cell lines. **A**, **D** Colony formation assay of EML4-ALK-positive NSCLC cell lines, H3122 (V1) and H2228 (V3), treated with inhibitors against ERBB and ALK. Colonies of >50 cells grown were visible after 10 days in the presence of inhibitors, which were replaced every 72 h. Doses of the inhibitors used for the clonogenic assay: ERL (5 μM), SAP (200 nM), and LOR (100 nM). **B**, **C**, **E**, **F** Survival fraction percentages of H3122 and H2228 cells treated with inhibitors. Data represent the mean of three independent biological replicates; the bars denote ±SD. **p < 0.01, ****p < 0.0001 in comparison to DMSO by two-way ANOVA. **G**, **H** H3122 and H2228 were treated with either LOR (100 nM) for 4 h, ERL (5 μM) for 2 h or in combination. **I**, **J** H3122 and H2228 cells were treated with either LOR (100 nM) for 4 h, SAP (200 nM) for 2 h, or in combination. Representative western blots of phosphorylated and total protein expressions were used to assess the relative abundance in treated cells. β-actin was used as a loading control. Data represent the mean of two independent biological replicates.

Using clonogenic assays, we observed a strong anti-proliferative effect of the combination of LOR(100 nM) with either ERL (5 μM) or SAP (SAP) (200 nM) in both EML4-ALK+cell lines (Fig. 4A–F). Inhibition of ERBB receptors with either ERL or SAP alone had a minimal impact on colony formation and cell proliferation in both EML4-ALK-harbouring cells (Fig. 4A–F). In contrast, no significant anti-proliferative effect was observed in A549 cells after single and combination treatments with ERBB and ALK inhibitors (Supplementary Fig. S7A–C). To understand how these different treatments affect signalling processes, we evaluated the status of phosphorylation of EGFR, ERBB2, ERBB3 and ALK kinases, and the activity of RAS/MAPK and PI3K/AKT signalling pathways after a 4-hour incubation with either single or combination of inhibitors (Table 1; Table 2). Upon LOR monotherapy, the abundance of EGFR^Y1173^ and ERBB2^Y1196^ phosphorylation were subsequently increased predominantly in the H3122 (EML4-ALK-V1) cell line, whereas ERBB3^Y1289^ expression was elevated preferentially in H2228 (EML4-ALK V3) cells (Fig. 4G, J; Supplementary Fig. S8A, B), which is in agreement with the phosphopeptide analysis (Figs. 1 and 2). Overall, LOR treatment led to a twofold increase of ERBB3^Y1289^ phosphorylation in EML4-ALK V3-harbouring cell lines (H2228) compared to EML4-ALK V1 (H3122). Whereas EGFR^Y1173^ and ERBB2^Y1196^ phosphorylation was significantly increased predominantly in H3122 (EML4-ALK V1) compared to H2228 cells (Supplementary Fig. S8A, B). These differences reflect the differential genetic background in each EML4-ALK cell line and their signalling dependency on different proteins. Conversely, 2-hour treatment with either ERL, SAP or in combination with LOR (4 h; 100 nM) reduced the activity of ERBB proteins including EGFR^Y1173^, ERBB3^Y1289^, and ERBB2^Y1196^ phosphorylation in both EML4-ALK-driven cell lines (Fig. 4G–J; Supplementary Fig. S8A, B). The activity of ERBB2, the preferred dimerization partner of ERBB3, was significantly disrupted after the treatment with ERL in both H3122 and H2228 cell lines (Fig. 4G, H; Supplementary Fig. S8A, B). In contrast, SAP by itself abolished ERBB2^Y1196^ activity in both cell lines (Fig. 4I, J; Supplementary Fig. S8A, B).

**Table 2:** Antibodies used for western blotting (WB) and dilutions.

Antibody	Supplier	Identifier	WB dilution
Anti-ALK (D5F3) rabbit mAb	CST	3633	1:1000
Anti-ALK (31F12) mouse mAb	CST	3921	1:1000
Anti-phospho-ALK (Y1604) rabbit mAb	CST	3341	1:1000
Anti-phospho-EGFR (Y1173) rabbit mAb	CST	4407	1:1000
Anti-EGFR (D38B1) rabbit mAb	CST	4267	1:2000
Anti-phospho-ERBB3 (Y1289) rabbit mAb	CST	4791	1:1000
Anti-ERBB3 (1B2E) rabbit mAb	CST	4754	1:1000
Anti-phospho-ERBB2 (D66B7) (Y1196) rabbit mAb	CST	6942	1:1000
Anti-ERBB2 (D8F12) rabbit mAb	CST	4290	1:1000
Anti-β-actin (C-2) mouse mAb	Santa Cruz	sc-8432	1:1000
Anti-phospho-AKT (D9E) (S473) rabbit mAb	CST	2965	1:1000
Anti-AKT (40D4) mouse mAb	CST	2920	1:1000
Anti-phospho-ERK1/2 (D12.14.4E) (T202/Y204) rabbit mAb	CST	4370	1:1000
Anti-ERK1/2 rabbit mAb	CST	9102	1:1000
Anti-phospho-c-MYC (EPR17924) (S62) rabbit mAb	abcam	Ab185656	1:1000
Anti-c-MYC Rabbit mAb	CST	9402	1:1000

While LOR alone abolished ALK^Y1604^ phosphorylation in H3122 cells, there was a residual signal in the H2228 cells that was only eliminated in combination with SAP (Fig. 4G–J). We also observed a strong inhibition of ERK^T202/Y204^ and AKT^S473^ phosphorylation, showing loss of RAS/MAPK and PI3K/AKT signalling pathways upon the combination of ERBB and ALK inhibitors in both cell lines (Fig. 4G-J). Single treatments with either ERL or SAP reduced ERK^T202/Y204^ phosphorylation in H2228, but not in H3122 cells (Fig. 4G–J). It should be noted that SAP, but not ERL, monotherapy abolished the abundance of AKT^S473^ phosphorylation predominantly in H2228, but not in H3122 cells (Fig. 4G–J). In contrast, PI3K/AKT signalling pathway activities did not alter upon ERBB and ALK inhibition in the EML4-ALK-negative NSCLC cell line, A549 (Supplementary Fig. S7D, E). Whereas inhibition of ERK^T202/Y204^ phosphorylation was observed only after ERL and SAP inhibition in A549 cells (Supplementary Fig. S7D, E). C-MYC^S62^ phosphorylation was reduced only after ERL treatment in A549 cells, whereas the activity of c-MYC^S62^ was diminished upon combination of ERBB and ALK inhibitors in EML4-ALK-harbouring cell lines, suggesting the importance of c-MYC^S62^ in regulating cell proliferation pathways including RAS/MAPK and PI3K/AKT (Fig. 4G–J; Supplementary Fig. S7D, E). As expected, no ALK activity was present in A549 cells, and LOR had no impact on disrupting RAS/MAPK and PI3K/AKT signalling pathways in those cells (Supplementary Fig. S7D, E). Hence, these findings corroborate our observations that inhibition of ERBB and ALK is specifically disrupting RAS/MAPK and PI3K/AKT signalling pathways in cell lines harbouring EML4-ALK mutation.

To confirm whether ERBB proteins are important in controlling RAS/MAPK and PI3K/AKT signalling pathways, we conducted siRNA-mediated knockdown of *EGFR* and *ERBB3* in EML4-ALK+positive NSCLC cell lines. Consistent with the chemical inhibition of EGFR and ERBB3, depletion of either EGFR or ERBB3 led to a substantial reduction of ERK^T202/Y204^ and AKT^S473^ phosphorylation in both cell lines, but with a profound loss of AKT activity predominantly observed in EML4-ALK V3 cells (H2228) (Supplementary Fig. S8C, D). Similarly to ERBB and ALK inhibition, the combination of ALK inhibition (LOR) with either EGFR or ERBB3 knockdown diminished ERK^T202/Y204^ and AKT^S473^ phosphorylation in both cell lines (Supplementary Fig. S8C, D). As previously observed in ERL and SAP treatments, the EGFR and ERBB3 knockdown led to a profound reduction of ERK^T202/Y204^ and AKT^S473^ activities in H2228 (EML4-ALK V3) cells compared to H3122 (EML4-ALK V1) (Fig. 4G, J; Supplementary Fig. S8C, D). It is known that ERBB2-ERBB3 heterodimer receptor promotes cell proliferation preferentially via PI3K/AKT signalling pathway, therefore inhibition of the complex by SAP disrupts its preferred signalling pathway [19]. Furthermore, the combination of ERBB inhibitor or knockdown with LOR increases cell death and reduces proliferation in both cell lines by inhibiting oncogenic and adaptive response signalling. Taken together, inhibiting the ERBB3 and ALK pathways represents a potential route to selective targeting of EML4-ALK-driven cells, particularly the ones harboring the EML4-ALK V3 mutation.

### Heregulin-mediated ERBB3 activation alters signalling and cell survival in constitutively active EML4-ALK+positive NSCLC cells

From the phosphopeptide analysis and cellular assays, we identified elevated expression of the ERBB3 and ERBB2 proteins after LOR treatment in EML4-ALK+positive NSCLC cells, we therefore investigated the effect of ligand-induced ERBB receptor activation in the context of TKI inhibition. EML4-ALK-harbouring NSCLC cells were stimulated with heregulin β-1 (HRG), an ERBB3 ligand, and the phosphorylation of ERBB receptors and signalling pathways were analysed. We confirmed phosphorylation of ERBB2 and ERBB3 after 1- and 2-hours induction with HRG ligand (10 ng/ml) in both serum-starved NSCLC cell lines (Supplementary Fig. S9A, B). As expected, activation of HRG-ERBB2-ERBB3 protein complex increased the abundance of ERK^T202/Y204^ and AKT^S473^ phosphorylation, and consequently, activation of RAS/MAPK and PI3K/AKT signalling pathways (Supplementary Fig. S9A, B). HRG induction also led to an increase of EGFR^Y1173^ activity by activating HRG-EGFR-ERBB3 heterodimers and therefore contributing to further activation of RAS/MAPK and PI3K/AKT signalling pathways (Supplementary Fig. S9A, B). The activation of RAS/MAPK and PI3K/AKT pathways by HRG-ERBB2-ERBB3 or HRG-EGFR-ERBB3 complexes led to an abundance of C-MYC^S62^ phosphorylation in both EML4-ALK-driven NSCLC cell lines (Supplementary Fig. S9A, B).

**Fig. 5:**
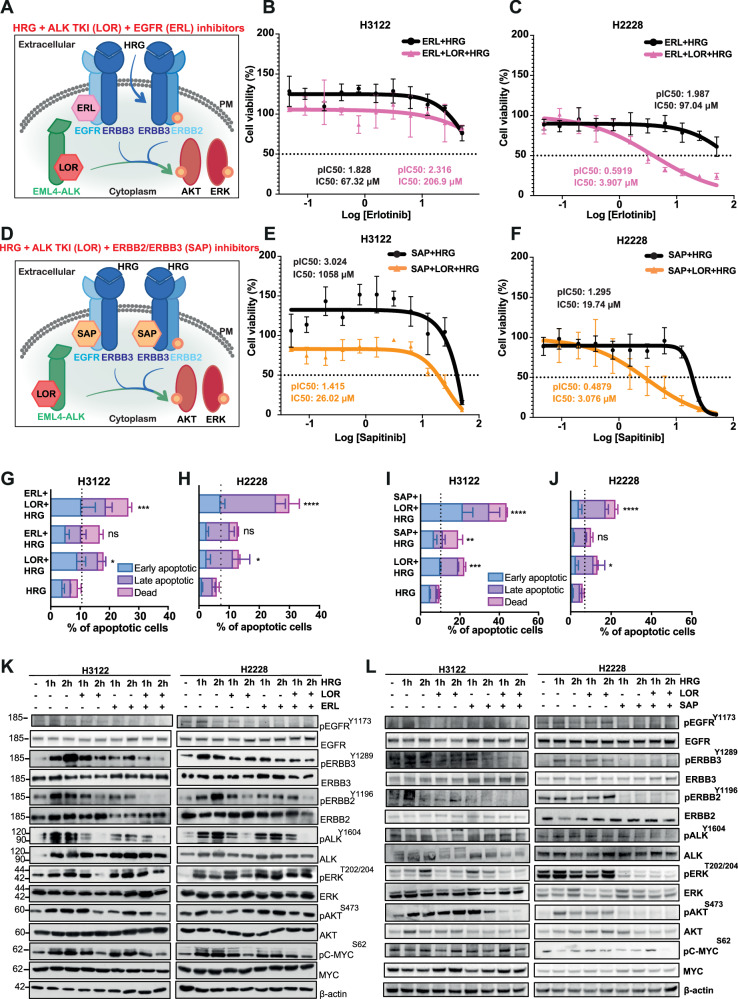
Heregulin β-1 induction increases the sensitivity of ERBB and ALK inhibition in EML4-ALK-harbouring cell lines. **A** Model of the combination of ALK TKI (Lorlatinib-LOR) and EGFR (Erlotinib-ERL) treatment targeting EML4-ALK fusion cytoplasmic protein and up-regulated phospho EGFR receptor on the membrane, respectively. Heregulin β-1 (HRG) ligand binds to ERBB3 receptor and promotes heterodimerization with EGFR and ERBB2 receptors on the membrane and subsequently activation of PI3K/AKT and MAPK signaling pathways. Lorlatinib inhibition feeds back into activation of AKT and ERK proteins. HRG-ERBB2/ERBB3 heterodimer is still active (Yellow phospho-Y) in the presence of erlotinib. **B**, **C** H3122 and H2228 serum-starved cells were treated with either LOR (3.12 nM), ERL (5 μM), or in combination in the presence of HRG for 72 h. Cell viability was determined using CellTiter-Glo assays. The IC_50_ values were calculated using Prism 10.0 software. Data represent the mean of three biological replicates in each column; the bars denote ±SD. **D** Model of the combination of ALK TKI (LOR) and ERBB2/ERBB3 (SAP) treatment targeting EML4-ALK fusion cytoplasmic protein and up-regulated phospho ERBB2/ERBB3 receptors on the membrane, respectively. HRG ligand binds to ERBB3 receptor and promotes heterodimerization with EGFR and ERBB2 receptors on the membrane. **E, F** H3122 and H2228 serum-starved cells were treated with either LOR (3.12 nM), SAP, or in combination in the presence of HRG for 72 h. Cell viability was determined using CellTiter-Glo assays. The IC_50_ values were calculated using Prism 10.0 software. Data represent the mean of three biological replicates in each column; the bars denote ±SD. H3122 and H2228 cells were treated; with either LOR (100 nM), **G**, **H** ERL (5 μM), **I**, **J** SAP (200 nM) or in combination in the presence of HRG for 48 h before analysis by annexin V-based flow cytometry. Histograms represent the percentage of cells in apoptosis and were classified as early apoptotic, late apoptotic, and dead. Data represent the mean of four independent biological replicates; the bars denote ±SD. *p < 0.5, **p < 0.01, ****p < 0.0001 in comparison to DMSO by two-way ANOVA. **K**, **L** H3122 and H2228 serum-starved cells were stimulated with HRG for 1 or 2 h and treated with either LOR (3.12 nM) for 4 h, ERL (5 μM) or SAP (200 nM) for 2 h or in combination. Representative western blots of phosphorylated and total protein expressions were used to assess the relative abundance in treated cells. β-actin was used as a loading control. Data representative of two independent biological replicates.

To assess the impact of HRG-ERBB2-ERBB3 and HRG-EGFR-ERBB3 signalling axis on cell viability and proliferation in EML4-ALK-harbouring NSCLC cells, we treated H3122 and H2228 cells with either ERL, SAP alone or in combination with LOR in the presence of HRG ligand (Fig. 5A, F). HRG binds to the ERBB3 receptor and promotes heterodimerization with EGFR and ERBB2 receptors (Fig. 5A, D). The single or combination treatments of ERL and LOR had a minimal effect in H3122 cells in the presence of HRG (Fig. 5B). In contrast, the combination of ERL plus LOR was more effective in reducing cell viability with a ~20-fold lower IC_50_ than ERL alone in H2228 (Fig. 5B, C). Interestingly, we observed greater sensitivity of HRG-stimulated H2228 cells against SAP either as a single treatment or in combination with LOR compared to H3122 cells (Fig. 5E, F). Interestingly, monotherapy with SAP had greater loss of cell viability with a ~50-fold lower IC_50_ in HRG-stimulated H2228 compared to HRG-stimulated H3122 cells (Fig. 5E, F). Consistent with the trends in the cell viability assays, the combination of LOR with either ERL or SAP induced higher levels of apoptosis compared to monotherapies in HRG-induced H3122 and H2228 cells (Fig. 5G, J; Supplementary Fig. S10A, B). Loss of EGFR and EML4-ALK activities after dual ERL and LOR treatment led to a near complete inhibition of cell proliferation in H3122 and H2228 cells, as assessed by colony formation assay (Supplementary Fig. S10C–E). However, both inhibitors had a profound effect on H2228 colony formation either with single or combination treatments, suggesting a strong dependency on ERBB receptor signalling for their proliferation (Supplementary Fig. S10C, E). Interestingly, monotherapy with ERL significantly decreased cell proliferation in H2228, but not in H3122 cells (Supplementary Fig. S10C, E). Activation of ERBB3 heterodimers by HRG in negative-EML4-ALK NSCLC cell line treated with either ERL or in combination with LOR had a minimal impact on cell viability with similar IC_50_ values (Supplementary Fig. S11A). Whereas SAP mono- or dual-treatment with LOR led to a 3-fold lower IC_50_ and increased sensitivity compared to ERL in HRG-stimulated A549 cells (Supplementary Fig. S11B). Consistent with the trends in the cell viability assay, the combination of LOR with either ERL or SAP did not induce high levels of apoptosis in HRG-induced A549 cells (Supplementary Fig. S11C, D). Hence, these data suggest the importance of HRG-ERBB signalling axes for cell proliferation and survival in EML4-ALK+positive NSCLC cell lines.

Taking a step further to elucidate the molecular mechanisms of ERBB and ALK signalling crosstalk, we analysed the effect of ERL, SAP and LOR treatments in HRG-stimulated EML4-ALK-driven cell lines. ERL mono-treatment had a minimal impact on the level of ERBB3^Y1289^, whereas the levels of EGFR^Y1173^ and ERBB2^Y1196^ phosphorylation were reduced in HRG-induced H3122 and H2228 cells (Fig. 5K). Interestingly, we observed a significant reduction of EGFR^Y1173^, ERBB2^Y1196^, and ERBB3^Y1289^ phosphorylation after treatment with ERL and LOR in both HRG-induced cell lines (Fig. 5K). However, the combination of ERL and LOR did not affect the MAPK and PI3K/AKT signalling pathways in HRG-stimulated H2228 cells (Fig. 5K). But this was not the case for the H3122-HRG induced cells, as we observed a reduced activity of ERK^T202/Y204^ and AKT^S473^ proteins upon ERL and LOR combination (Fig. 5K). The persistent activity of ERBB3^Y1289^ in HRG-stimulated H2228 cells maintains the levels of ERK and AKT phosphorylation and subsequently, downstream signalling pathways to overcome ERL and LOR inhibition. In contrast, SAP treatment used either as a single or in combination with LOR had a profound impact on MAPK and PI3K/AKT signalling pathways in both EML4-ALK-driven cell lines (Fig. 5L). Loss of ERBB3 activity by SAP inhibitor significantly reduced the EGFR^Y1173^, ERBB2^Y1196^, and ERBB3^Y1289^ phosphorylation, leading to disruption of ERBB3 heterodimers (Fig. 5L). Subsequently, this led to a significant reduction of ERK^T202/Y204^, AKT^S473^ and c-MYC^S62^ phosphorylation in both EML4-ALK-harbouring cell lines (Fig. 5L). Interestingly, the EML4-ALK V3-harbouring H2228 cells displayed higher sensitivity to SAP treatments with significant loss of loss of ERK, AKT, and c-MYC activities compared to EML4-ALK V1-harbouring H3122 cells (Fig. 5L). In the EML4-ALK-negative NSCLC cell line, ERL and SAP let to a reduction of ERK^T202/Y204^ and AKT^S473^ phosphorylation, whereas ALK inhibition did not alter MAPK and PI3K/AKT signalling pathways (Supplementary Fig. S11E, F). It should be noted that neither ERL, SAP or LOR disrupted c-MYC^S62^ phosphorylation in A549 cells (Supplementary Fig. S11E, F). As expected, reduction of EGFR^Y1173^, ERBB2^Y1196^ and ERBB3^Y1289^ phosphorylation was observed upon ERL and SAP treatments (Supplementary Fig. S11E, F). Taken together, these data show a differential impact of ERBB3 receptor activation by HRG, whereas H2228 cells, but not H3122, were sensitized to both ERL/LOR and SAP/LOR combinations. The loss of ERBB3 activity had a more significant impact on the survival and proliferation of H2228 than in H3122 cells, suggesting HRG-induced dependency on ERBB3 activity.

### Growth-inhibitory effect of AKT inhibitor and ALK-TKI combination in EML4-ALK-harbouring NSCLC cell lines

**Fig. 6:**
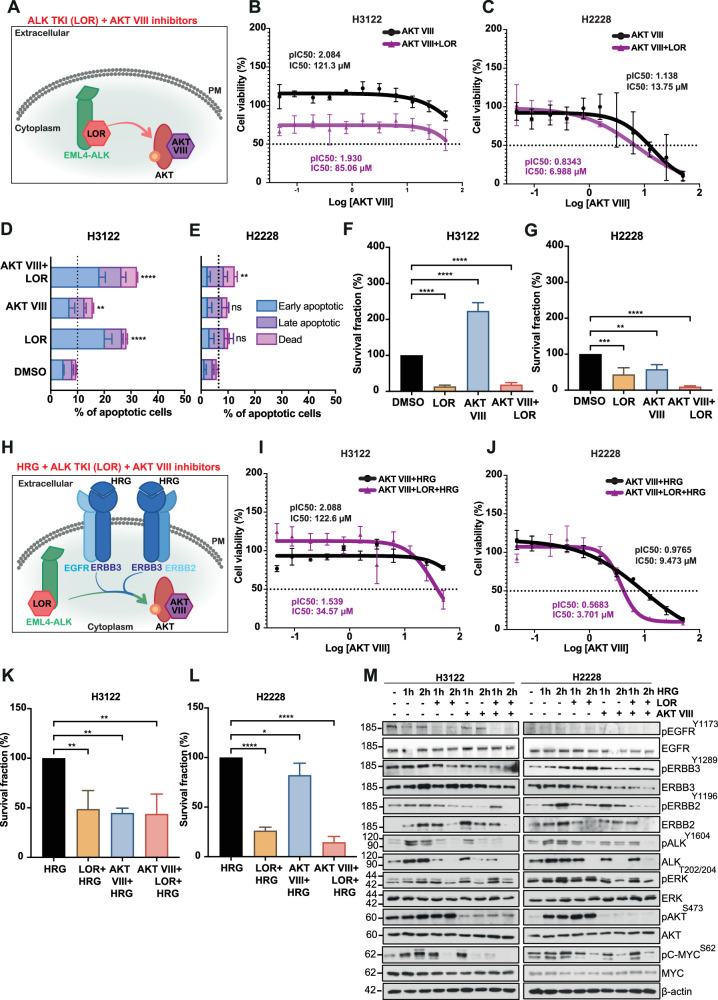
Loss of cell proliferation and downstream signalling after AKT and ALK inhibition in EML4-ALK-driven cell lines. **A** ALK inhibition by lorlatinib let to inactivation of the kinase, but increased phosphorylation (yellow arrow) of AKT activity in EML4-ALK+positive NSCLC cells. Model of the combination of ALK TKI (Lorlatinib-LOR) and AKT (AKT VIII) treatment targeting EML4-ALK fusion cytoplasmic protein and up-regulated phospho AKT protein, respectively. **B**, **C** H3122 and H2228 cells were treated with either LOR (3.12 nM), AKT VIII (1 μM) or in combination for 72 h. Cell viability was determined using CellTiter-Glo assays. The IC_50_ and pIC_50_ values were calculated using Prism 10.0 software. Data represent the mean of four biological replicates in each column; the bars denote ±SD. **D**, **E** H3122 and H2228 cells treated with either LOR (100 nM), AKT VIII (1 μM) or in combination for 48 h before analysis by annexin V-based flow cytometry. Histograms represent the percentage of cells in apoptosis and were classified as early apoptotic, late apoptotic and dead. Data represent the mean of four independent biological replicates; the bars denote ±SD. **p < 0.01, ****p < 0.0001 in comparison to DMSO by two-way ANOVA. **F**, **G** Survival fraction percentages of H3122 and H2228 cells treated with inhibitors from clonogenic assay in Supplementary Fig. S12G. Data represent the mean of three independent biological replicates; the bars denote ±SD. **p < 0.01, ***p < 0.001, ****p < 0.0001 in comparison to DMSO by two-way ANOVA. **H** Heregulin β-1 (HRG) ligand binds to ERBB3 receptor to promote heterodimerization with EGFR and ERBB2 receptors on the membrane. AKT activity is upregulated by ALK inhibition, lorlatinib, and by signals through ERBB receptors (green/blue arrows). Dual inhibition of ALK and AKT to block their activities. **I**, **J** EML4-ALK-positive NSCLC cell lines were serum-starved and treated with either LOR (3.12 nM), AKT VIII or in combination in the presence of HRG for 72 h. Cell viability was determined using CellTiter-Glo assays. The IC_50_ and pIC_50_ values were calculated using Prism 10.0 software. Data represent the mean of four biological replicates in each column; the bars denote ±SD. **K**, **L** Survival fraction percentages of H3122 and H2228 HRG-stimulated cells treated with compounds from colony formation assay in Supplementary Fig. S12H. Data represent the mean of three independent biological replicates; the bars denote ±SD. *p < 0.05, **p < 0.01, ****p < 0.0001 in comparison to DMSO by two-way ANOVA. **M** H3122 and H2228 serum-starved cells were stimulated with HRG for 1 or 2 h and treated with either LOR (3.12 nM) for 4 h, AKT VIII (1 μM) for 2 h or in combination. Representative western blots of phosphorylated and total protein expressions were used to assess the relative abundance in treated cells. β-actin was used as a loading control.

As we identified from peptide microarray and enrichment analysis, LOR treatment markedly increased the phosphorylation of RAC-alpha serine/threonine-protein kinase (AKT1) and phosphoinositide-3-kinase regulatory subunit 1 (PIK3R1/P85A) in EML4-ALK-harbouring cell lines (Fig. 1C–E; Fig. 2A–D; Fig. 6A). The PI3K/AKT signalling pathway identified as one of the top three signalling hubs in LOR-treated H3122 and H2228 cells (Fig. 2C–E). We therefore assessed whether AKT inhibition in combination with ALK-TKI (LOR) has anti-proliferative effects in EML4-ALK-driven NSCLC cell lines (Fig. 6A). Indeed, AKT inhibition, using AKT VIII compound, led to a significant reduction of cell viability in H2228, but not in H3122 cells (Fig. 6B, C; Supplementary Fig. S12A–D). EML4-ALK-driven cell lines displayed higher sensitivity to the combination of AKT inhibition with ALK-TKI, LOR, compared to single AKT VIII treatment (Fig. 6B, C; Supplementary Fig. S12A–D). We next examined whether the high levels of cytotoxicity from the combination of LOR and AKT VIII inhibitors were a result of apoptotic cell death. A single treatment with LOR or AKT VIII led to an ~2–3-fold increase in apoptosis in H3122 cells, assessed by Annexin V + PI staining (Fig. 6D; Supplementary Fig. S12E). Instead, neither LOR nor AKT VIII single treatment was sufficient to induce apoptosis in H2228 cells (Fig. 6E; Supplementary Fig. S12F). The loss of both AKT and ALK activities led to a significant increase in apoptosis in both NSCLC cell lines (Fig. 6D, E; Supplementary Fig. S12E, F). Dual inhibition of ALK and AKT in EML4-ALK-driven cell lines with modest concentrations of LOR and AKT VIII inhibitors led to a profound loss of cell proliferation as assessed by clonogenic assays (Fig. 6F, G; Supplementary Fig. S12G). Interestingly, AKT inhibition significantly reduced cell proliferation in H2228 cells, in contrast to H3122 cells (Fig. 6F, G; Supplementary Fig. S12G). Together, these data demonstrate that loss of AKT and ALK differentially affects EML4-ALK-driven cell lines, with H3122 more sensitive to ALK inhibition and H2228 more sensitive to AKT inhibition. This suggests that H2228 cells rely on AKT1 activity to overcome ALK inhibition and therefore, AKT1 is a key component of a bypass pathway independent of ALK activity.

Since ERBB2-ERBB3 heterodimer receptor activates PI3K/AKT pathway, we investigated whether activation of ERBB receptor by HRG will alter cell proliferation and survival of LOR-treated EML4-ALK-harbouring cell lines (Fig. 6H). We then reasoned that HRG induction would activate ERBB2-ERBB3 and EGFR-ERBB3 receptors on the membrane, and subsequently, inhibition of EML4-ALK by LOR will further increase AKT activity and subsequently trigger PI3K/AKT signalling pathway (Fig. 6H). In HRG-induced H3122 cells, we have seen a significant change in cell survival with a ~2.5-fold lower IC_50_ after dual inhibition with LOR and AKT VIII inhibitors compared to non-HRG induced H3122 cells (Fig. 6B, I). In contrast, reduced cell survival was observed with either single or combination of LOR and AKT VIII inhibitors in HRG-induced H2228 cells (Fig. 6J). Interestingly, H3122 cells exhibited less sensitivity to either single or combination of AKT VIII and ALK inhibition in the presence of HRG compared to non-induced cells (Fig. 6B, I).

We performed Annexin V assays and confirmed loss of ALK and AKT activities by combination treatments led to significant increase of cell death in both HRG-induced cell lines (Supplementary Fig. S12I, J). HRG-induced H2228 cells displayed increased cell death compared to non-HRG-induced cells, suggesting HRG is shifting the dependency of cell survival of H2228 cells to the ERBB3-ERBB2 signalling axis from EML4-ALK oncoprotein-generated signalling activities (Fig. 6E; Supplementary Fig. S12J). In line with the above set of data, clonogenic assays demonstrated a significant loss of cell proliferation by either single or combination treatment in EML4-ALK-harbouring cell lines (Fig. 6K, L; Supplementary Fig. S12H). It should be noted that AKT and ALK inhibition had a minimal effect on cell viability in non-induced and HRG-induced non-cancerous epithelial BEAS2B cells (Supplementary Fig. S13A, B). In the EML4-ALK-negative NSCLC cell line, A549, AKT inhibition in the presence or absence of LOR did not affect their cell viability with IC_50_ values of 150.9 and 135.87 μM, respectively, neither induced cell death (Supplementary Fig. S14A–C). Activation of ERBB3 heterodimers with HRG ligand increased the sensitivity to AKT inhibition compared to the non-induced cells, however this effect did not accompany with elevated cell death in A549 cell line (Supplementary Fig. S14D–F).

As HRG ligand promotes ERBB3 heterodimerization, we investigated the effects on downstream signalling activation of LOR and AKT VIII inhibitors in HRG-induced EML4-ALK-driven cell lines. For this experiment, cells were serum-starved and treated with HRG ligands for one or two hours. The presence of HRG (after 1 or 2 h) elevated ERBB3 and ERBB2 phosphorylation and promoted heterodimerization, and rapidly induced AKT and ERK phosphorylation (Fig. 6M). As seen before, LOR treatment had a lesser impact on ALK activity and subsequently, c-MYC activation in H2228 cells compared to H3122 cells, underlying the role of other factors that maintain ALK activity in H2228 cells (Fig. 6M). AKT phosphorylation was maintained with LOR treatment and HRG induction in both cell lines (Fig. 6M), consistent with phosphotyrosine data from the peptide array (Fig. 1C, D). Interestingly, we observed an increase of ALK and MYC phosphorylation after HRG induction in both cell lines (Fig. 6M). Either single or combination treatments after 2-hour HRG induction led to a significant loss of EGFR, ERBB2 and ERBB3 receptor activities and consequently, disruption of AKT phosphorylation in both cell lines (Fig. 6M). We observed a small reduction of ERK phosphorylation after the combination of LOR and AKT VIII treatment in both cell lines, suggesting that HRG-ERBB2-ERBB3 axis is sufficient to drive activation of MAPK signalling pathway (Fig. 6M). AKT inhibition alone or in combination with LOR had a minimal effect on the PI3K/AKT and MAPK signalling pathways in the EML4-ALK-negative NSCLC cell line, A549 (Supplementary Fig. S14G). Additionally, the activities of EGFR, ERBB2, ERBB3 receptors were not affected upon AKT and ALK inhibition in A549 cells (Supplementary Fig. S14G). Overall, these data suggest the sensitivity of EML4-ALK-driven cell lines to combining LOR and AKT VIII inhibitors and the importance of AKT/PI3K signalling pathway in EML4-ALK+positive NSCLC, specifically in the EML4-ALK V3 cell line (H2228).

### ERBB and AKT inhibitors reduce cell proliferation of LOR-resistant H2228 cells via loss of MAPK and PI3K/AKT signalling pathways

**Fig. 7:**
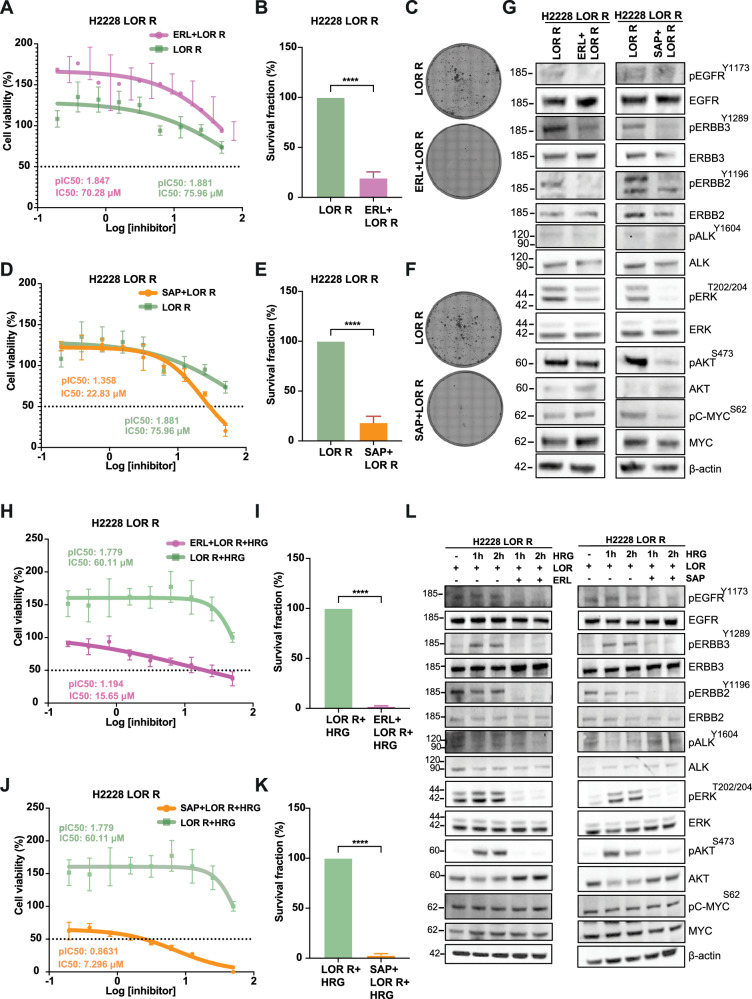
Loss of EGFR and ERBB3 activities sensitises lorlatinib-resistant H2228 cells. **A**, **D** H2228 LOR R cells were grown in lorlatinib (LOR) (500 nM) and treated with either erlotinib (ERL) (5 μM) or sapitinib (SAP) (200 nM) for 72 h. Cell viability was determined using CellTiter-Glo assays. The IC_50_ and pIC_50_ values were calculated using Prism 10.0 software. Data represent the mean of four biological replicates in each column; the bars denote ±SD. **B**, **C**, **E**, **F** Survival fraction percentages of LOR R H2228 cells treated with inhibitors, ERL (5 μM) or SAP (200 nM) from clonogenic assay. Data represent the mean of three independent biological replicates; the bars denote ±SD. ** ****p < 0.0001 in comparison to LOR R by student *t* test. **G** H2228 LOR R cells were grown in LOR (500 nM) and treated with either ERL (5 μM) or SAP (200 nM) for 2 h. Representative western blots of phosphorylated and total protein expressions were used to assess the relative abundance in treated cells. β-actin was used as a loading control. Data represent the mean of two independent biological replicates. **H**, **J** H2228 LOR R cells were grown in LOR (500 nM) and treated with ERL (5 μM) or SAP (200 nM) in the presence of heregulin β-1 (HRG) for 72 h. Cell viability was determined using CellTiter-Glo assays. The IC_50_ and pIC_50_ values were calculated using Prism 10.0 software. Data represent the mean of four biological replicates in each column; the bars denote ±SD. **I**, **K** Survival fraction percentages of H2228 LOR R cells treated with inhibitors, erlotinib or sapitinib. Data represent the mean of three independent biological replicates; the bars denote ±SD. ****p < 0.0001 in comparison to LOR R by student *t* test. **L** H2228 LOR R serum-starved cells were stimulated with HRG for 1 or 2 h and treated with were grown in LOR (500 nM) and treated with either ERL (5 μM) or SAP (200 nM) for 2 h. Representative western blots of phosphorylated and total protein expressions were used to assess the relative abundance in treated cells. β-actin was used as a loading control.

Considering the efficacy of both ERL and SAP in inhibiting the activities of ERBB receptors in EML4-ALK-driven NSCLC cells, we next hypothesized that their pronounced anti-proliferative activity could sensitize the LOR-resistant (LOR R) H2228 cells. To mimic the context of adaptive resistance to ALK inhibitors, we first generated a resistant cell line derived from the parental H2228 cells with acquired resistance to LOR obtained by long-term exposure to increasing concentrations of the compound (Supplementary Fig. S15A, B). We validated the resistance of LOR R H2228 cell line versus the parental H2228 cell line using cell viability assay with increasing doses of LOR for 72 h. The LOR R-H2228 cells displayed an ~5-fold lower IC_50_ compared to parental H2228 cell line (Supplementary Fig. S15A, B). We next examined the effect of ERL and SAP in LOR R H2228 cells. The LOR R H2228 cells displayed a minimal response to ERL treatment in cell viability assays, however this was accompanied with a significant loss of cell proliferation using colony formation assay (Fig. 7A–C; Supplementary Fig. S15C). In contrast, ERBB3 inhibitor (SAP) had a profound impact on cell viability of LOR R H2228 cell line with a ~ 3-fold lower IC_50_ compared to LOR R H2228 cells that was followed by a significant reduction of cell proliferation using colony formation assay (Fig. 7D–F; Supplementary Fig. S15D). However, analysis of apoptotic profiles after ERL and SAP treatment did not display cell death via apoptotic pathway in LOR R H2228 cells (Supplementary Fig. S15E–G).

To follow up on this observation, we next examined the activities of ERBB receptors and their effect on MAPK and PI3K/AKT signalling pathways upon erlotinib and SAP in resistant-LOR R H2228 cells. ERL treatment reduced ERK^T202/Y204^ and AKT^S473^ phosphorylation in drug-resistant LOR R H2228, while ERBB3 inhibition by SAP abolished ERK^T202/Y204^ and AKT^S473^ phosphorylation, and subsequently c-MYC^S62^ activity leading to loss of MAPK and PI3K/AKT pathways (Fig. 7G). Immunoblotting confirmed the loss of the EGFR and ERBB3 activities by ERL and SAP, respectively, in LOR R H2228 cell line (Fig. 7G). Notably, ALK phosphorylation was maintained in drug-resistant LOR R H2228 cell line (Fig. 7G). Together, these findings indicate that treatment with SAP reduces cell proliferation in drug-resistant LOR R H2228 cells.

Considering the importance of ERBB receptors in regulating proliferation pathways in LOR-sensitive EML4-ALK NSCLC cell lines, we next investigated how activation of ERBB3 receptor by HRG could impact cell proliferation and survival of LOR-resistant LOR R EML4-ALK cells. The treatment with ERL reduced the cell viability of LOR R H2228 cells with IC_50_ value of 15.65 μM versus the LOR R H2228 with IC_50_ value of 60.11 μM (Fig. 7H; Supplementary Fig. S15H). This effect was accompanied with a significant loss of cell proliferation in ERL + LOR R H2228 cells measured by colony formation assay (Fig. 7I; Supplementary Fig. S15M). The addition of SAP has a profound impact on cell viability of HRG-induced LOR R H2228 cells with an ~8-fold lower IC_50_ compared to alone HRG-LOR R H2228 cells (Fig. 7J; Supplementary Fig. S15I). Additionally, dual treatment with SAP and LOR significantly reduced cell proliferation in HRG-LOR R H2228 cells compared to LOR alone (Fig. 7K; Supplementary Fig. S15M). However, neither ERL nor SAP treatment induced cell death via apoptotic pathway in LOR R H2228 cells suggesting that cancer cells have slower cell growth (cytostasis) without induction of cell death (Supplementary Fig. S15E–G and S15J–L). In agreement with the loss of cell proliferation, both ERL and SAP led to loss of EGFR^Y1173^, ERBB2^Y1196^ and ERBB3^Y1289^ phosphorylation in HRG-induced resistant LOR R H2228 cells (Fig. 7L). The combination of ERL or SAP with LOR abolished ERK^T202/Y204^ and AKT^S473^ phosphorylation and subsequently, loss of RAS/MAPK and PI3K/AKT signalling pathways in HRG-stimulated LOR R H2228 cells (Fig. 7L).

**Fig. 8:**
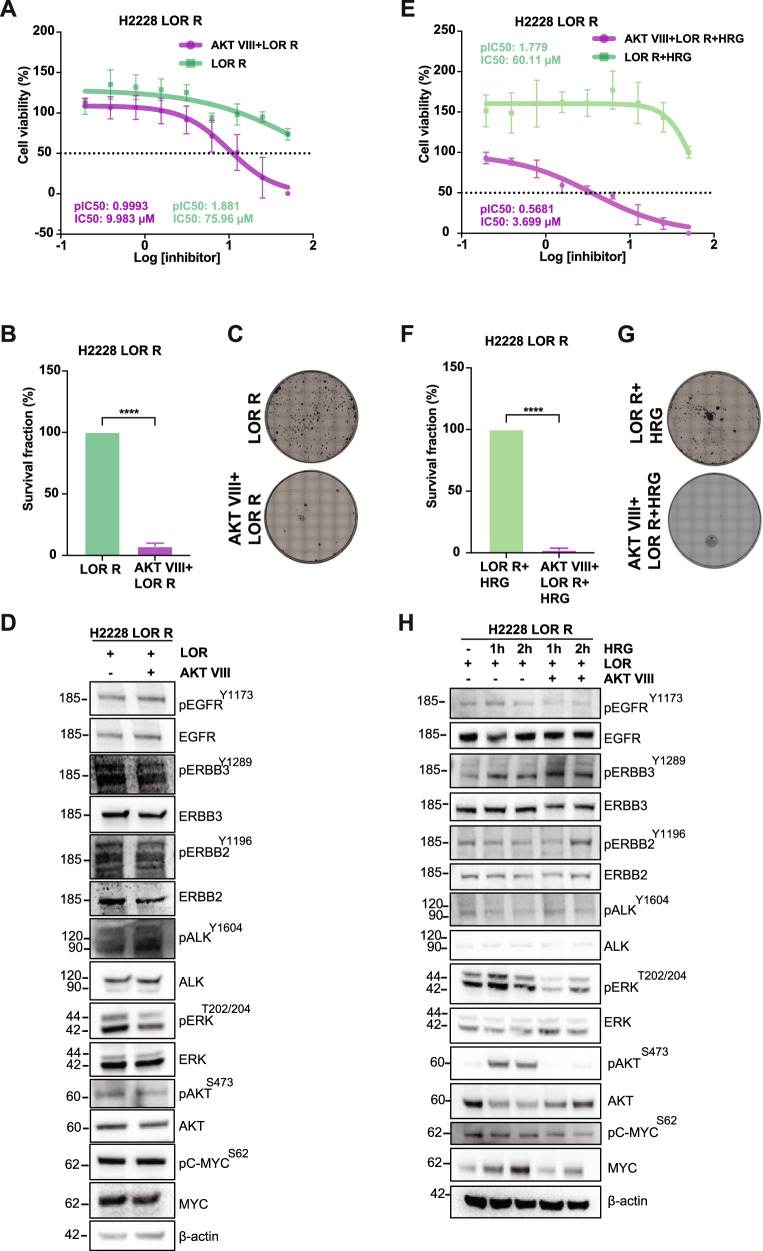
AKT inhibition induces cytotoxicity in lorlatinib-resistant H2228 cells. **A** H2228 LOR R cells were grown in lorlatinib (LOR) (500 nM) and treated with AKT VIII inhibitor (1 μM) for 72 h. Cell viability was determined using CellTiter-Glo assays. The IC_50_ and pIC_50_ values were calculated using Prism 10.0 software. Data represent the mean of four biological replicates in each column; the bars denote ±SD. **B** Survival fraction percentages of H2228 LOR R cells treated with AKT VIII inhibitor (1 μM) from colony formation assay. Data represent the mean of three independent biological replicates; the bars denote ±SD. ****p < 0.0001 in comparison to LOR R by student *t* test. **C** Colony formation assay of drug-resistant H2228 LOR R treated with AKT VIII inhibitor. Colonies of >50 cells grown were visible after ten days in the presence of inhibitors, which were replaced every 72 h. Doses of the inhibitors used for the clonogenic assay: AKT VIII (1 μM) and LOR (500 nM). **D** H2228 LOR R cells were treated with AKT VIII inhibitor for 2 h and lysed for western blotting analysis. Representative western blots of phosphorylated and total protein expressions were used to assess the relative abundance in treated cells. β-actin was used as a loading control. **E** H2228 LOR R cells were grown in LOR (500 nM) and treated with AKT VIII (1 μM) in the presence of heregulin β-1 (HRG) for 72 h. Cell viability was determined using CellTiter-Glo assays. The IC_50_ and pIC_50_ values were calculated using Prism 10.0 software. Data represent the mean of four biological replicates in each column; the bars denote ±SD. **F** Survival fraction percentages of HRG-induced H2228 LOR R cells treated with AKT VIII inhibitor from colony formation assay. Data represent the mean of three independent biological replicates; the bars denote ±SD. ****p < 0.0001 in comparison to LOR R by student *t* test. **G** Colony formation assay of drug-resistant H2228 LOR R serum-starved cells treated with AKT VIII inhibitor in the presence of HRG. Colonies of >50 cells grown were visible after 10 days in the presence of inhibitors, which were replaced every 72 h. Doses of the inhibitors used for the clonogenic assay: AKT VIII (1 μM) and lorlatinib (500 nM). **H** H2228 LOR R serum-starved cells were stimulated with HRG for 1 or 2 h and treated with were grown in LOR (500 nM) and treated with AKT VIII (1 μM) for 2 h. Representative western blots of phosphorylated and total protein expressions were used to assess the relative abundance in treated cells. β-actin was used as a loading control.

Finally, we probed the contribution of AKT to the survival and proliferation of LOR-resistant H2228 cells. The addition of AKT VIII inhibitor re-sensitized these cells to LOR with IC_50_ of 9.983 μM compared to 75.96 μM with LOR alone (Fig. 8A; Supplementary Fig. S16A). AKT inhibition significantly reduced cell proliferation in LOR R H2228 that was accompanied with a reduction of MAPK and PI3K/AKT signalling pathways as observed by immunoblot analysis of ERK^T202/Y204^ and AKT^S473^ phosphorylation (Fig. 8B–D). In the presence of HRG, in which leads to activation of ERBB3 heterodimers, AKT inhibition led to a significant reduction of cell viability in resistant LOR R H2228 cells with a very low IC_50_ value of 3.699 μM versus a 60.11 μM of LOR R H2228 cells (Fig. 8E; Supplementary Fig. S16B). Furthermore, AKT inhibition via AKT VIII compound led to a significant loss of cell proliferation in HRG-stimulated LOR R H2228 cells, and this effect was accompanied with reduction of ERK^T202/Y204^ and AKT^S473^ activities suggesting disruption of MAPK and PI3K/AKT signalling pathways in resistant LOR R H2228 cells (Fig. 8F–H). We observed that AKT inhibition did not induce cell death in LOR R H2228 cells via the apoptotic pathway (Supplementary Fig. S16C–F). Nevertheless, targeting AKT proved effective in re-sensitizing LOR-resistant H2228 cells (EML4-ALK V3), as well as the parental H2228 cells.

## Discussion

**Fig. 9:**
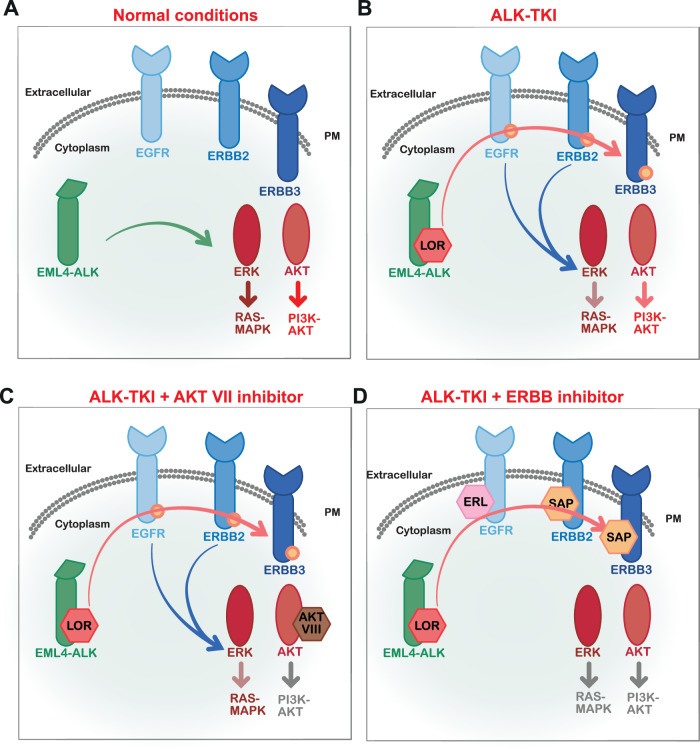
Models summarizing the mechanism of crosstalk between EML4-ALK and ERBB receptors and downstream signaling. **A** Schematic model showing the mechanism of downstream activation by EML4-ALK. Under normal conditions, EML4-ALK fusion protein induces activation of RAS/MAPK and PI3K/AKT signalling pathways. **B** Loss of EML4-ALK activity by lorlatinib (LOR) induces EGFR, ERBB2, ERBB3 phosphorylation, and subsequent ERK and AKT activation. **C** Dual inhibition of EML4-ALK and AKT abolishes PI3K/AKT signalling, but ERBB membrane receptors still signal and activate RAS/MAPK signalling pathways. **D** Inhibition of EML4-ALK and ERBB activities abrogates downstream signalling pathways. The ERBB and EML4-ALK signalling axis is required for the survival of EML4-ALK-harboring NSCLC cells.

Constitutively activated oncoproteins drive key signalling pathways that allow cells to escape regulatory mechanisms of homeostasis, such as negative feedback pathways that control the duration of signalling output [13]. Loss of these negative feedback regulatory pathways can further disrupt signalling pathways and favour cell survival. It has been frequently observed that targeted drug therapies, although effective at inhibiting driver oncoproteins and their signalling pathways, can also disrupt negative feedback pathways. It has been shown before that mTORC1 inhibition activated parallel growth factor pathways, including PI3K/AKT and increased ERK levels in patients and cell lines (breast, melanoma, and colon) treated with rapamycin [20]. In a similar context, our study has uncovered that EML4-ALK inhibition by the 3rd generation ALK-TKI, LOR, led to significant upregulation of ERBB receptors (EGFR, ERBB2 and ERBB3) and AKT, and thereby promotes bypass activation of RAS/MAPK and PI3K/AKT signalling pathways (Fig. 9B). In the presence of constitutively expressed EML4-ALK fusion protein, the RAS/MAPK and PI3K/AKT signalling pathways are highly active and promote cell proliferation (Fig. 9A). Loss of AKT and EML4-ALK activities dampens the PI3K/AKT pathway and increases cell death, but RAS/MAPK pathway remains active (Fig. 9C). However, the disruption of the crosstalk between EML4-ALK and ERBB receptors has a profound impact on both RAS/MAPK and PI3K/AKT signalling pathways and consequently, reduced proliferation and cell survival (Fig. 9D). Furthermore, the inhibition of ERBB or AKT activities had a marginal effect on the cell viability and proliferation of LOR-resistant cells, implying that ERBB and AKT activities act in part to promote resistance to LOR. The crosstalk between EML4-ALK fusion protein and ERBB membrane receptors is thus a potential target to overcome adaptive resistance of ALK-TKI inhibition.

Resistance to ALK-TKIs is achieved either by on-target mechanisms via mutations within the kinase domain of ALK or off-target mechanisms via activation of bypass signalling pathways and overexpression of alternative receptor tyrosine kinases (RTK). Several studies reported the presence of both EML4-ALK and EGFR rearrangements in patients with lung cancer [11, 21–23]. One such example is the overexpression of ERBB family of RTK observed in EML4-ALK-positive NSCLC tumours. In a recent case, patient with NSCLC harboured both EGFR and EML4-ALK mutations after acquiring resistance to the EGFR TM790M inhibitor [21]. Analysis of primary tumours from multifocal lung adenocarcinomas reported ~5% of EGFR/ALK co-alterations [22]. Another clinical case of an EML4-ALK+ positive NSCLC patient reported the co-existence of EGFR mutation and a response to both EGFR and ALK-tyrosine kinase inhibitors [23]. In addition, a crizotinib-treated NSCLC patient developed resistance with the presence of EGFR as a secondary mutation [11]. We detected high levels of phosphorylation in ERBB family members upon acute inhibition with LOR, suggesting activation of bypass signalling pathways such as PI3K/AKT and RAS/MAPK via those membrane receptors. EGFR signalling has been previously reported to be upregulated and control signalling cascades in drug-resistance models after ALK inhibition [24, 25]. Katayama et al. [24] have recently reported the EGFR signalling in EML4-ALK+positive models after LOR drug-resistance. Development of drug resistance to ALK-TKI leads to hyperactivation and development of secondary mutations in kinases such as EGFR, ERBB3, it is therefore worth exploring further the combination of ALK-TKI and ERRB inhibitors to overcome any emerge resistance of these tumours.

ERBB3 overexpression occurs in several cancers and has a central role in the development of drug-resistance to several tyrosine kinase inhibitors including ALK-TKI [10]. Indeed, a recent study by Kim et al. reported high levels of ERBB3 expression in both primary and secondary tumours in EML4-ALK+positive NSCLC patients that was associated with poor prognosis [26]. Similarly, high levels of ERBB3 expression and activation of bypass signalling pathways were observed in EML4-ALK+positive NSCLC cells treated with either 1^st^ or 2^nd^ generation ALK-TKI such as crizotinib, ceritinib and alectinib [27, 28]. Here, we have detected high levels of ERBB3 expression in EML4-ALK positive NSCLC cells after acute inhibition with LOR and subsequently, activation of PI3K/AKT signalling pathway, and we propose ERBB3 as a mechanism of LOR-treated EML4-ALK-rearragened NSCLC cells to escape from ALK-TKI inhibition. In multiple cancers, the overexpression of ERBB3 is associated with high levels of ERBB2 and EGFR [29, 30]. In fact, our phospho proteomic data identified a subset of ERBB2, ERBB3 and EGFR proteins to be upregulated in LOR-treated ALK-rearranged cell lines. Interestingly, H2228 cells exhibited higher sensitivity to ALK inhibition in the presence of heregulin (HRG), suggesting a shift on the dependency of cell survival via the ERBB3 signalling axis. Hence, dual inhibition of ALK and ERBB3 activities has a significant impact on the survival of cells harbouring EML4-ALK. A recent study has reported high levels of HRG and high phospho ERBB3, and consequently, high AKT and ERK phosphorylation upon cisplatin treatment in bladder cancer, suggesting a therapeutic strategy of targeting ERBB3 in those cells [31]. We are currently exploring the role of HRG in EML4-ALK+positive NSCLC patients and the mechanism between ERBB3 and EML4-ALK activation in those patients. A more detailed understanding of the molecular wiring in these pathways is required to elucidate the mechanism that connects ERBB3 and EML4-ALK in drug resistance.

Given the importance of ERBB3 to be frequently upregulated in several cancers and its implication in drug-resistance, a huge effort has been put for the development of anti-ERBB3 therapies. Several monoclonal antibodies (mAbs) against ERBB3 are currently in clinical trials to assess their efficacy in several cancers including lung cancer [32]. A notable example of ERBB3 monoclonal antibody is patritumab deruxtecan, which prevents the binding of HRG to ERBB3 [33, 34]. Single treatment of patritumab or in combination with EGFR inhibitor ERL sensitises ERL-resistant NSCLC models [34]. Seribantumab (MM-121) is another human monoclonal antibody against ERBB3 that inhibits its dimerization by preventing HRG binding to ERBB3 [32]. Seribantumab displayed anti-proliferative effects in several cancer models and its currently in clinical phase I and II against advanced solid tumours with NRG1 gene fusion [35]. We demonstrated anti-proliferative effects with the combination of LOR and SAP inhibitors in EML4-ALK+harbouring NSCLC cells, resulting in a significant loss of ERBB2, ERBB3, and subsequently, loss of PI3K/AKT and RAS/MAPK signalling pathways. Interestingly, the presence of HRG confer resistance to ERBB3 and AKT inhibitors in H3122 cells compared to H2228 cells, it is therefore important to consider the type of EML4-ALK variant for any future therapy combinations of AKT-TKI and ERBB3 inhibition. The IC_50_s we determined using our best combination of inhibitors were too high to be confident of clinical translation, and more effective combinations of therapeutics are needed. It will therefore be interesting to explore in depth the anti-proliferative effects of targeting both ERBB and ALK proteins in EML4-ALK-rearranged NSCLC with small molecule and antibody-based inhibitors to overcome adaptive drug-resistance of ALK-TKIs in NSCLC.

While EML4-ALK has a driving role in activating downstream signalling cascades such as RAS/MAPK, PI3K/AKT and JAK/STAT3 in EML4-ALK-rearranged cancers, other proteins also feedback into those signalling pathways and might contribute to disease progression [5, 36]. As well as the overexpression of ERBB proteins following ALK-TKI treatment in EML4-ALK-rearranged NSCLC cells, we also identified high levels of AKT1 and PI3K activities upon LOR treatment. Indeed, one of the major signalling pathways upregulated in LOR-treated EML4-ALK+positive NSCLC cells was the PI3K/AKT pathway. This is frequently de-regulated in several cancers and commonly found to contribute to drug resistance [37]. A novel AKT inhibitor, capivasertib, has been recently approved by FDA for treatment of patients with advanced or metastatic hormone-receptor (HR)-positive and HER2-negative breast cancer [38]. This opens exciting avenues for the use of capivasertib in combination with tyrosine kinase inhibitors for the treatment of ALK-TKI resistance. In fact, our in vitro data demonstrated strong anti-proliferative effects in EML4-ALK+positive NSCLC cells treated with both AKT VIII and LOR inhibitors. In addition, we observed a reduction of phospho ERK levels upon AKT VIII inhibition in the presence of growth factor heregulin. The crosstalk between PI3K/AKT and RAS/MAPK signalling pathways allows cancer cells to evade apoptosis [39]. There is therefore a clear rationale for targeting the PI3K/AKT proliferation pathway to overcome drug-resistance in several cancers including NSCLC.

While our work focused predominantly in characterizing the impact of ERBB and AKT proteins in LOR-treated EML4-ALK+driven cells, other non-RTK proteins including PTPN11 (SHP2) identified in our microarray analysis (Fig. 1E; Supplementary Fig. S1A–D). Protein-tyrosine phosphatase non-receptor type 11 (PTPN11), also known as SHP2, is a tyrosine phosphatase important for regulating signals through RTK/RAS/ERK signalling cascade [40, 41]. Upon ALK inhibition, the Tyr580 (Y580) and Tyr62 (Y62) residues of PTPN11 were upregulated, while the Tyr542 (Y542) residue was downregulated in both NSCLC cells lines (Fig. 1E; Supplementary Fig. S1A–D). Phosphorylation of PTPN11 at Tyr542 and Tyr580 are the two most important post-translational modification for PTPN11 activation and frequently implicated in various signalling pathways including RAS/MAPK and PI3K/AKT [42]. It is known that PTPN11 Y542 and Y580 residues interacts with RTKs such as EGFR and with GRB2 to link RTK to the RAS/MAPK signalling pathways, as well as to the adaptor GAB1 protein to activate PI3K/AKT signalling cascade [43–45]. Upregulation of PTPN11 phosphatases have been reported to mediate resistance to ALK-TKI in ALK+ anaplastic large cell lymphoma and in a variety of other cancers [42, 46]. The loss of phosphorylation of PTPTN11 at Y542, but not at Y580, might suggest the importance of each residue in controlling signalling activation in EML4-ALK+driven NSCLC cell lines. Phosphorylation of PTPN11 at Y62 impact the activation of RAS/MAPK and PI3K/AKT signalling pathways for cell survival and proliferation, and has been implicated to drug resistance in cancer [47]. PTPN11 is a pleotropic protein with important functions in cell signalling for proliferation and survival, and PTPN11 dysregulation of tyrosine phosphorylation is associated with several cancers by regulating migration and invasion, tumor microenvironment and cancer immunity [42, 48]. It is therefore an interesting target to overcome drug resistance in cancer. Future work could explore in depth the functional role of the PTPN11 phosphorylated residues (Y62, Y542 and Y580) in signalling activation, drug resistance and cancer immunity in the EML4-ALK + NSCLC.

Understanding the molecular pathways that lead to adaptive resistance mechanisms will enable us to suggest new ways of targeting ALK-TKI-resistant NSCLC. In our study, we identified proteins that are upregulated upon acute treatment with LOR in EML4-ALK+positive NSCLC cells. We focused on ERBB family and AKT1 proteins that activate RAS/MAPK and PI3K/AKT signalling pathways. We explored the anti-proliferative effects of targeting those adaptive mechanisms to overcome drug-resistance in EML4-ALK+positive NSCLC cells. The presence of HRG has a significant impact on the survival of EML4-ALK+harbouring cell lines, consistent with the role of ERBB3 as a bypass pathway upon ALK inhibition. The ERBB and AKT activities are essential for the proliferation and survival of LOR-resistant cells, independently of ALK activity. The discovery of a mechanistic link between acute inhibition of ALK with the TKI LOR, the activities of ERBB3 and AKT1, and the important role of HRG/ERBB3 signalling axis in drug response, opens up new avenues for targeting the ERBB3 and AKT signalling axis in the optimisation of therapy for ALK + NSCLC patients.

## Material and methods

### Cell culture and drug treatments

The human NSCLC cell lines H3122, H2228, BEAS2B, and A549 were obtained from ATCC. All cells were maintained in culture at 37 °C in a 5% CO_2_ atmosphere for a maximum of 2 months. We relied on the provenance of the original collection for authenticity. Cell lines were regularly tested for mycoplasma contamination using a highly sensitive and specific PCR-based assay (EZ-PCR Mycoplasma kit, Geneflow). NCI-H3122, NCI-H2228, BEAS2B and A549 were cultured in RPMI-1640 medium. All media were from Invitrogen-GIBCO and supplemented with 10% heat-inactivated fetal bovine serum (FBS), 100 IU/mL penicillin, and 100 mg/mL streptomycin. LOR (PF-06463922), ERL, AKT VIII inhibitor, and SAP were purchased from Selleck Chemicals, and stock solutions were prepared in DMSO. Unless otherwise indicated, the following compounds were added to cells for either 1 or 2 h: LOR (3.12 nM); ERL (5 µM); AKT VIII (1 µM); SAP (200 nM) (Table 1). The compounds were diluted in a fresh media before each experiment, and control cells were treated with the same volume of DMSO.

### Drug-resistant cell lines

The parental H2228 cell line was exposed to a low concentration (25 nM) of LOR compound until cells could proliferate fully in the presence of it. Cells were then exposed to a 50% higher concentration, and the process was repeated until the H2228 cell line was resistant to 500 nM LOR. The resistant LOR R H2228 cell line was cultured in 500 nM LOR by adding fresh drug after every passage. LOR-resistant H2228 cell line was assessed every 4 weeks to assess their capacity to maintain resistance to LOR by using cell viability assay.

### siRNA knockdown

Cells were seeded at 50% confluency in RPMI medium. After 24 h, cells were transfected with siRNAs duplexes (final concentration of 25 nmol/L) in Opti-MEM Reduced Serum Medium with DharmaFECT Transfection Reagent 4 (Thermo Fisher Scientific) according to manufacturer’s instructions. ON-TARGETplus siRNA duplexes were used against human EGFR (J-003114-11, J-003114-13) and ERBB3 (J-003127-10, J-003127-12) or GAPDH (J-004253-06). Cells were prepared for annexin V-FITC or western blotting analysis 52 h after transfection of siRNA duplexes.

### ERBB3 dimerization using HRG β-1 induction

Recombinant human HRG β-1 was obtained from peprotech (100-03). Cells were seeded in a complete growth medium. After 24 h, cells were serum-starved using medium without growth factor (FBS) and P/S for 24 h. HRG ligand (10 nM) was added to cells for either 1 or 2 h to induce ERBB3 dimerization, unless otherwise stated.

### Cell viability assay

Cells were seeded in a complete growth medium in 96-well plates at 3 × 10^3^ cells per well. After 24 h, the cells were incubated with either LOR (3.12 nM), ERL (5 μM), SAP (200 nM), AKT VIII (1 μM), or in combination in the presence of 10% of FBS. For HRG induction, cells were serum-starved overnight before 72 h treatment. After 72 h of treatment, cell viability was determined using the CellTiter-Glo^®^ Luminescent Cell Viability Assay (Promega, Madison, WI, USA) according to the manufacturer’s instructions. The half-maximal inhibitory concentration (IC_50_) values were calculated from dose-response curves (non-linear regression log (inhibitors) vs response-(four parameters) in the Prism 10.0 software (GraphPad, San Diego, CA, USA). For the cell titer glo (CTG) assays, LOR final dose was 3.12 nM, as we wanted to assess the effect of ERL and SAP without killing cells. It should be noted that 3.12 nM LOR was sufficient to block ALK activity (Fig. 5N).

### Cell extracts and western blotting analysis

Whole-cell lysates were prepared in RIPA lysis buffer (Cell Signalling Technology, Danvers, MA, USA) containing a protease inhibitor cocktail (Tech & Innovation^TM^, Bucheon, Korea) and a phosphatase inhibitor cocktail (45065; Santa Cruz, CA, USA). Proteins were separated on an 8% or 10% SDS-PAGE gel, transferred to polyvinylidene fluoride membranes using an iBlot^TM^ dry blotting system (Invitrogen). Immunoblot analysis was performed with the following primary antibodies (Table 2):

Secondary antibodies were rabbit or mouse horseradish peroxidase-labelled secondary antibodies (1:10,000; Amersham, UK). The blots were visualised using the SuperSignal West Pico Chemiluminescent Substrate (Thermo Fisher Scientific-Pierce, Rockford, IL, USA). Source data from Western blotting is shown in Supplementary material.

### Apoptosis assay

Annexin V apoptosis assay (FITC Annexin V, Biolegend) was performed according to manufacturer’s instructions. Cells seeded in six-well plates were treated with either LOR (100 nM), ERL (5 μM), AKT VIII (1 μM), SAP (200 nM) or in combination for 48 h. For siRNA of EGFR and ERBB3, cells were seeded in 6-well plates and depleted with siRNA oligos in the presence or absence of LOR (100 nM) for 48 h. Cells were harvested and collected by centrifugation. About 5 × 10^5^ cells were incubated in 100 µL Annexin binding buffer, 5 µL Annexin V-FITC, and 10 µL propidium iodide (PI) for 15 min on ice. Samples were kept in the dark and diluted in a further 400 µL Annexin binding buffer before analysis by flow cytometry. Samples were processed on a CytoFLEX S flow cytometer (Beckman Coulter) and analysed using Kaluza (Beckman Coulter). For the annexin V assays, we used LOR at 100 nM concentration as we wanted to assess the impact of the short treatment recapitulating the phospho peptide array data.

### Colony formation assay

Twenty-four hours post treatment, cells were trypsinised and reseeded in a six-well plate at 400 cells per well and left to incubate for 10 days. Colonies were then fixed with methanol 100%, stained (0.5% crystal violet), and counted using DotCount software. The surviving fraction (SF) for each treatment was calculated using plating efficiency of treated samples/plating efficiency of control. Data represent the mean of three independent experiments ± SD.

### Peptide chip array

Biological triplicates of H3122 and H2228 cells were treated for 4 h with 100 nM LOR. Cells were washed with PBS on ice and lysed with M-PER mammalian extraction buffer (Pierce) containing Halt phosphatase and Halt protease inhibitor cocktails (Pierce) on ice. Cell extracts were cleared by centrifugation for 10 min at 12,000 rpm at 4 °C, and protein concentration were determined by BCA assays (Pierce). Aliquots of lysate were snap-frozen in liquid nitrogen. 5 μg of protein extract was used for the PTK array protocol (V1.9). Measurements were performed on a PamStation12 from PamGene (Wolvenhoek 10,‘s-Hertogenbosch, Netherlands). For the PTK analysis, a single-step reaction was performed, where cell lysates, ATP, and fluorescein isothiocyanate (FITC)-labeled pY20 antibody were incubated on the chip. The phosphorylation status of the individual Tyrosine peptides (186 in total) was monitored by fluorescence detection in real-time. Signal intensities were analysed in the BioNavigator software (PamGen) as a function of time and expressed as LFC versus DMSO treatment after 4 h.

### Statistical and bioinformatic analysis

All quantitative data represent means and standard deviation (SD) of at least three independent experiments. Statistical analyses were performed using, unpaired Student’s *t* test, one-way ANOVA, and two-way ANOVA from Prism 10.0 software. ****P < 0.0001, ***P < 0.001, **P < 0.01, *P < 0.05. Venn diagrams were generated in Venny 2.1 (https://bioinfogp.cnb.csic.es/tools/venny/index.html). Network plots were generated in Cytoscape 3.9.0 using STRING and were analysed by cytoHubba plugin [49]. Heat maps were generated in NG-CHM Interactive Builder version 2 (https://build.ngchm.net/NGCHM-web-builder/Select_Matrix.html?v=2.22.0). Proteomaps were generated in Bionic Visualization Proteomaps version 2 (bionic-vis (uni-greifswald.de)). The UKA predicted differential kinase activity in LOR compared to DMSO. UKA algorithm used knowledge from databases of kinase-substrate relationships (Phosphosite PLUS, Phospho.elm, UniPROT, Reactome, Kinexus (PhopshoNet), and Human Protein Reference Database. MFS was calculated based on the sum of the kinase significance and kinase specificity (represents the specificity of the change in kinase activity). The KEGG pathway enrichment and GO processes were analysed using Enrichr (https://maayanlab.cloud/Enrichr/). Analysis of KEGG pathway annotation enrichment was generated from gene sets of upregulated phosphorylated tyrosine proteins (Data File S2) from ALK TKI-treated H3122 and H2228 cell lines. Statistical significance of peptides by Fisher’s exact test (*p* < 0.05). For the GO enrichment analysis related to biological process and molecular function of upregulated phosphotyrosine proteins from Data File S2.

## Supplementary information

Supplementary Text

Supplementary Figs.

Supplemental Material Raw Blots

Dataset 1

Dataset 2

Dataset 3

Dataset 4

## Data Availability

The phosphopeptide chip array data needed to evaluate the conclusions in the paper are present in the manuscript, supporting excel files-data files and the supplementary materials.
