# Supplementary material for: Targeting ERBB3 and AKT to overcome adaptive resistance in EML4-ALK-driven non-small cell lung cancer
Source: Cell Death Dis. 2024 Dec 18;15(12):912. doi: 10.1038/s41419-024-07272-7 (PMC11655848; doi:10.1038/s41419-024-07272-7)
Supplement: Supplementary file 1 — Supplementary Text [file 41419_2024_7272_MOESM1_ESM.docx]

Targeting ERBB3 and AKT to overcome adaptive resistance in EML4-ALK-driven non-small cell lung cancer

**Josephina Sampson^1*^, Hyun-min Ju^2^, Nan Zhang^1^, Sharon Yeoh^1^, Jene Choi^2*^, Richard Bayliss^1*^**

**Figure S1. Heat map of detailed tyrosine phosphorylated peptides from PTK peptide array in H3122 and H2228 cell lines**

**A-B.** Heat map illustrates up- or down-regulation of tyrosine phosphorylated peptides after 4-hour lorlatinib treatment in H3122 and H2228 cell lines as were analysed by PTK peptide array. Log fold change (LFC) values were used to generate the heat map (n=3 biological replicates). Significant peptides (*p* <0.05) were clustered using hierarchical order and Euclidean/ward algorithm. **C-D.** Tables summarizing the phosphorylated tyrosine peptides that were downregulated after 4 hour treatment with lorlatinib in H3122 and H2228 cell lines (n=3).

**Figure S2. List of proteins involved in signalling pathways in H3122 and H2228-lorlatinib treated cells from betweenness centrality analysis**

**A-B.** Proteins were sorted according to their communication paths and information flow using betweenness centrality algorithm in cytoHubba plugin in Cytoscape. Proteins with high betweenness centrality represent key proteins that influence the flow in signalling pathways.

**Figure S3. List of proteins involved in signalling pathways in H3122 and H2228-lorlatinib treated cells from degree of the nodes analysis**

**A, C.** Proteins (nodes) were sorted according to their number of links using degree centrality algorithm in cytoHubba plugin in Cytoscape. Proteins with high degree centrality represent key proteins that hold most of information in signalling pathways. **B, D.** Bar plot summarizing the top 10 proteins from A and B using degree centrality algorithm in Cytoscape plugin cytoHubba based on significantly upregulated phosphorylation of tyrosine peptides from PTK peptide array.

**Figure S4. Proteomaps of biological processes based on the significantly upregulated tyrosine peptides**

**A-B.** Proteomaps generated to visualise the distributions of phosphorylated tyrosine peptides (LFC values) from PTK assay in H3122 and H2228. Each tile in the map represents a protein; size is proportional to its LFC value. Tile colours represent different protein. Proteins that share similar colour and near to each other means they share similar function. Proteomaps were generated by Bionic Visualization. The different colour labelling indicates the biological processes. **C-D.** Proteomaps generated to visualise the distributions of phosphorylated tyrosine peptides (LFC values) from PTK assay in H3122 and H2228. Each tile in the map represents a protein that belongs to biological process; size is proportional to its LFC value. **E-F.** Table summarizing the statistical analysis of erlotinib/sapitinib/lorlatinib combinations in H3122 and H2228 cell lines from the Figure 3B-C, E-F. Data represent the mean of four independent biological replicates; * *p* < 0.05, **** *p* < 0.0001 by two-way ANOVA and Šídák's and Tukey’s multiple comparisons tests.

**Figure S5. No anti-proliferative effects in EML4-ALK-negative NSCLC cell lines upon dual inhibition of ERBB and ALK**

**A-B**. BEAS2B or **C-D.** A549 cells were treated with either erlotinib (ERL) or sapitinib (SAP) -/+ lorlatinib (LOR) (3.12 nM) for 72 hours. Cell viability was determined using CellTiter-Glo assays. The IC_50_ and pIC_50_ values were calculated using Prism 10.0 software. Data represent the mean of four independent biological replicates in each column; the bars denote ±SD. Table summarizing the statistical analysis of erlotinib/sapitinib/lorlatinib combination in A549 cell line. Data represent the mean of four independent biological replicates; **** *p* < 0.0001 by two-way ANOVA and Tukey’s multiple comparisons tests.

**Figure S6. Apoptotic profiles of chemical inhibition and knockdown of EGFR and ERBB3 in NSCLC cell lines**

**A-B.** H3122 and H2228 cells **C-E.** A549 cells were treated with either LOR (100 nM), ERL (5 μM), SAP (200 nM) or in combination for 48 hours before analysis by annexin V-FITC/PI staining. Data represent the mean of four biological replicates in each column; the bars denote ±SD. * *p* < 0.05 in comparison to DMSO by two-way ANOVA. **F-I.** H3122 and H2228 cells were mock-depleted or depleted with siRNAs against EGFR or ERBB3 and in combination with LOR (100 nM) for 48 hours before analysis by annexin V-based flow cytometry. Dot-plot profiles of untreated and treated cells were generated using Kaluza analysis software. Data represent the mean of three independent biological replicates in each column; the bars denote ±SD. The number of cells in each quadrant were shown as percentage.

**Figure S7. Chemical inhibition of ERBB and ALK proteins in EML4-ALK-negative NSCLC cell line**

**A.** Colony formation assay of A549 cells treated with inhibitors against ERBB and ALK. Colonies of >50 cells grown were visible after ten days in the presence of inhibitors, which were replaced every 72 hours. Doses of the inhibitors used for the clonogenic assay: ERL (5 μM), SAP (200 nM) and LOR (100 nM). **B-C.** Survival fraction percentages of A549 cells treated with inhibitors. Data represent the mean of three independent biological replicates; the bars denote ±SD. ** *p* < 0.01 in comparison to DMSO by two-way ANOVA. **D-E**. A549 cells were treated with either LOR (100 nM) for 4 hours, ERL (5 μM) or SAP (200 nM) for 2 hours or in combination. Representative western blots of phosphorylated and total protein expressions were used to assess the relative abundance in treated cells. β-actin was used as a loading control. Western blotting analysis was performed in two independent biological replicates.

**Figure S8. Disruption of signalling pathways upon loss of EGFR and ERBB3 activities in H3122 and H2228 cells**

**A-B**. Histograms represent the relative intensity of EGFR^Y1173^, ERBB2^Y1196^ and ERBB3^Y1289^ proteins upon each drug condition in H3122 and H2228 cell lines from Figure 4G-J. Protein expressions were normalised to β-actin loading control. Data represent the mean of two biological replicates in each column; the bars denote ±SD. ** *p* < 0.01 in comparison to DMSO by two-way ANOVA. **C-D**. H3122 and H2228 cells were mock-depleted or depleted with siRNAs against EGFR or ERBB3 for 52 hours before western blotting analysis. Representative western blots of phosphorylated and total protein expressions were used to assess the relative abundance in mock- and siRNA-depleted cells. β-actin was used as a loading control. Western blotting analysis was performed in two independent biological replicates.

**Figure S9. Activation of ERBB2/ERBB3 and EGFR/ERBB3 heterodimer receptors by heregulin ligand in EML4-ALK+positive NSCLC cell lines**

**A-B.** Serum-starved H3122 and H2228 cells were stimulated with heregulin β-1 (HRG-10 ng/ml) for 1 and 2 hours. Representative western blots of phosphorylated and total protein expressions were used to assess the relative abundance in treated cells. β-actin was used as a loading control. Western blotting analysis was performed in two independent biological replicates.

**Figure S10. Apoptosis profiles of erlotinib/sapitinib and -/+ lorlatinib treatments in HRG-induced EML4-ALK+positive NSCLC cell lines**

**A-B.** H3122 and H2228 cells were stimulated with heregulin β-1 (HRG) and treated with either LOR (100 nM), ERL (5 μM), SAP (200 nM) or in combination for 48 hours before analysis by annexin V-FITC/PI staining. Dot-plot profiles of untreated and treated cells were generated using Kaluza analysis software. The number of cells in each quadrant were shown as percentage. **C.** Colony formation assay of EML4-ALK-positive NSCLC cell lines, H3122 and H2228, were serum-starved and treated with inhibitors against ERBB and ALK in the presence of HRG. Colonies of >50 cells grown were visible after ten days in the presence of inhibitors, which were replaced every 72 hours. Doses of the inhibitors used for the clonogenic assay: ERL (5 μM) and LOR (100 nM). **D-E.** Survival fraction percentages of H3122 and H2228 cells treated with inhibitors from colony formation assay. Data represent the mean of three independent biological replicates; the bars denote ±SD. * *p* < 0.05, ** *p* < 0.01, **** *p* < 0.0001 in comparison to DMSO by two-way ANOVA.

**Figure S11. ERBB inhibitors did not affect the survival of Heregulin-stimulated negative-EML4-ALK NSCLC cell line**

**A-B.** A549 cells were stimulated with heregulin β-1 (HRG) and treated with either erlotinib (ERL) or sapitinib (SAP) -/+ lorlatinib (LOR) (3.12 nM) for 72 hours. Cell viability was determined using CellTiter-Glo assays. The IC_50_ and pIC_50_ values were calculated using Prism 10.0 software. Data represent the mean of four independent biological replicates in each column; the bars denote ±SD. **C-D.** A549 cells were stimulated with HRG and treated with either LOR (100 nM), ERL (5 μM), SAP (200 nM) or in combination for 48 hours before analysis by annexin V-FITC/PI staining. Data represent the mean of three independent biological replicates in each column; the bars denote ±SD. Treatments in comparison to HRG by two-way ANOVA. **E-F.** A549 serum-starved cells were stimulated with HRG for 1 or 2 hours and treated with either LOR (3.12 nM) for 4 hours, SAP (200 nM) for 2 hours or in combination. Representative western blots of phosphorylated and total protein expressions were used to assess the relative abundance in treated cells. β-actin was used as a loading control. Data representative of two independent biological replicates.

**Figure S12. The combinations of AKT VIII inhibitor and lorlatinib in non- and HRG-stimulated H3122 and H2228 cell lines**

**A-D.** Table summarizing the statistical analysis of AKT VIII/LOR combination in H3122 and H2228 cell lines from the Figure 6B-C. Data represent the mean of four independent biological replicates; ** *p* < 0.01, **** *p* < 0.0001 by two-way ANOVA and Tukey’s multiple comparisons tests. **E-F.** H3122 cells were treated with either LOR (100 nM) or AKT VIII (1 μM) in combination for 48 hours before analysis by annexin V-FITC/PI staining. Dot-plot profiles of untreated and treated cells were generated using Kaluza analysis software. Data represent the mean of three independent biological replicates in each column; the bars denote ±SD. The number of cells in each quadrant were shown as percentage. **G-H.** Colony formation assay of H3122 and H2228, were cyclin or serum-starved and treated with inhibitors against AKT and ALK in the presence of HRG. Colonies of >50 cells grown were visible after ten days in the presence of inhibitors, which were replaced every 72 hours. Doses of the inhibitors used for the clonogenic assay: AKT VIII (1 μM) and LOR (100 nM). **I-J.** H3122 and H2228 cells were treated; with either LOR (100 nM), AKT VIII (1 μM) or in combination in the presence of HRG for 48 hours before analysis by annexin V-based flow cytometry. Histograms represent the percentage of cells in apoptosis and were classified as early apoptotic, late apoptotic and dead. Data represent the mean of four independent biological replicates; the bars denote ±SD. ** *p* < 0.01, *** *p* < 0.001, **** *p* < 0.0001 in comparison to DMSO by two-way ANOVA.

**Figure S13. AKT inhibition did not affect the survival of non-cancerous epithelial BEAS2B cell line**

BEAS2B cells were **A.** treated with AKT VII -/+ lorlatinib (LOR) (3.12 nM) or **B.** stimulated with heregulin β-1 (HRG) and treated with AKT VIII -/+ lorlatinib (LOR) (3.12 nM) for 72 hours. Cell viability was determined using CellTiter-Glo assays. The IC_50_ and pIC_50_ values were calculated using Prism 10.0 software. Data represent the mean of four independent biological replicates in each column; the bars denote ±SD. Table summarizing the statistical analysis of AKT VIII/lorlatinib combination in BEAS2B cell line. Data represent the mean of four independent biological replicates; **** *p* < 0.0001 by two-way ANOVA and Tukey’s multiple comparisons tests.

**Figure S14. AKT inhibition did not affect the survival of Heregulin-stimulated negative-EML4-ALK NSCLC cell line**

A549 cells were **A.** treated with AKT VIII and -/+ LOR (3.12 nM) or **D.** stimulated with heregulin β-1 (HRG) and treated with AKT VIII -/+ LOR (3.12 nM) for 72 hours. Cell viability was determined using CellTiter-Glo assays. The IC_50_ and pIC_50_ values were calculated using Prism 10.0 software. Data represent the mean of four independent biological replicates in each column; the bars denote ±SD. Table summarizing the statistical analysis of AKT VIII/LOR combination in BEAS2B cell line from the panel 14A and 14D. Data represent the mean of four independent biological replicates; **** *p* < 0.0001 by two-way ANOVA and Tukey’s multiple comparisons tests. **B-C.** A549 cells were treated with AKT VIII and lorlatinib inhibitors for 48 hours before analysis by annexin V-FITC/PI staining; or **E-F.** A549 cells were stimulated with HRG and treated with either LOR (100 nM), AKT VIII (1 μM) or in combination for 48 hours before analysis by annexin V-FITC/PI staining. Data represent the mean of three independent biological replicates in each column; the bars denote ±SD. Treatments in comparison to HRG by two-way ANOVA. **G.** A549 serum-starved cells were stimulated with HRG for 1 or 2 hours and treated with either LOR (3.12 nM) for 4 hours, AKT VIII (1 μM) for 2 hours or in combination. Representative western blots of phosphorylated and total protein expressions were used to assess the relative abundance in treated cells. β-actin was used as a loading control. Data representative of two biological replicates.

**Figure S15. EGFR and ERBB3 inhibition sensitise lorlatinib-resistant H2228 cell line**

**A.** CellTiter-Glow assay of LOR R-resistant H2228 and parental H2228 cell lines, treated with indicated concentrations of lorlatinib for 72 hours. The IC_50_ and pIC_50_ values were calculated using Prism 10.0 software. Data represent the mean of four independent biological replicates in each column; the bars denote ±SD. **B.** Table summarizing the statistical analysis of parental versus LOR R-resistant in H2228 cell line. Data represent the mean of four independent biological replicates; **** *p* < 0.0001 by two-way ANOVA and Tukey’s multiple comparisons tests. **C.** Table summarizing the statistical analysis of ERL/LOR or **D.** SAP/LOR combination in H2228-LOR R cell line. Data represent the mean of four independent biological replicates; **** *p* < 0.0001 by two-way ANOVA and Tukey’s multiple comparisons tests. **E-F.** Drug-resistant-LOR R (500 nM) H2228 cells were treated with ERL (5 μM) or SAP (200 nM) inhibitors for 48 hours before analysis by annexin V-FITC/PI staining. Data represent the mean of three independent biological replicates in each column; the bars denote ±SD. * *p* < 0.05 of treatments in comparison to LOR R by two-way ANOVA. **G.** Dot-plot profiles of LOR R and ERL or SAP treated cells were generated using Kaluza analysis software. **H.** Table summarizing the statistical analysis of ERL/LOR or **I.** SAP/LOR combination in HRG-induced H2228-LOR R cell line. Data represent the mean of four independent biological replicates; *** *p* < 0.001, **** *p* < 0.0001 by two-way ANOVA and Tukey’s multiple comparisons tests. **J-K.** Histograms represent the percentage of cells in apoptosis and were classified as early apoptotic, late apoptotic and dead in HRG-induced LOR R treated cells with either ERL or SAP for 48 hours. **L.** Annexin V profiles of HRG induced LOR R H2228 cells treated with either ERL or SAP for 48 hours. Dot-plot profiles of HRG induced LOR R H2228 cells treated with either ERL or SAP were generated using Kaluza analysis software. **M.** Colony formation assay of LOR R H2228 stimulated with HRG and treated with ERL or SAP inhibitors. Colonies of >50 cells grown were visible after ten days in the presence of inhibitors, which were replaced every 72 hours.

**Figure S16. AKT inhibition in lorlatinib-resistant H2228 cell line**

**A.** Table summarizing the statistical analysis of LOR R versus LOR R/AKT VIII combination in resistant-LOR R H2228 cell line. **B.** AKT VIII/LOR combination in HRG-induced H2228-LOR R cell line. Data represent the mean of four independent biological replicates; * *p* < 0.05, ** *p* < 0.01, *** *p* < 0.001, **** *p* < 0.0001 by two-way ANOVA and Tukey’s multiple comparisons tests. **C-D.** Histograms represent the percentage of cells in apoptosis and were classified as early apoptotic, late apoptotic and dead in cyclin or HRG-induced LOR R treated cells with AKT VIII inhibitor for 48 hours. **E-F.** Annexin V profiles (dot-plot) of cyclin or HRG induced LOR R H2228 cells treated with AKT VIII inhibitor for 48 hours were generated using Kaluza analysis software.

**Data file S1. Table showing the LFC and *p* values for all Tyrosine phosphorylation from Pamgene kinase activity profiling assay**

Excel sheet summarizes the mean LFC values and *p* values from three biological replicates. H3122 and H2228 cells were treated for 4 hour with either DMSO (4 hour) or lorlatinib (100 nM). Data from data file S1 were used to generate Figure 1 and 2. Data file is in the online supplementary materials as an ‘.xlsx’ file.

**Data file S2. Table showing the LFC and *p* values of significant upregulated Tyrosine phosphorylated proteins**

Excel sheet summarizes the mean LFC values and *p* values from three biological replicates. H3122 and H2228 cells were treated for 4 hour with either DMSO or lorlatinib (100 nM). Data from data file S2 were used to generate Figure 2. Data file is in the online supplementary materials as an ‘.xlsx’ file.

**Data file S3. Table showing the mean and median kinase score values of kinases predicted using Upstream Kinase Analysis (UKA) algorithm in H3122 cell line**

Excel sheet summarizes the mean final and median final scores of predicted kinases using Upstream Kinase Analysis (UKA) algorithm. H3122 cells were treated for 4 hour with either DMSO or lorlatinib (100 nM). Data from data file S3 were used to generate Figure 2G. Data file is in the online supplementary materials as an ‘.xlsx’ file.

**Data file S4. Table showing the mean and median kinase score values of kinases predicted using Upstream Kinase Analysis (UKA) algorithm in H2228 cell line**

Excel sheet summarizes the mean final and median final scores of predicted kinases using Upstream Kinase Analysis (UKA) algorithm. H2228 cells were treated for 4 hour with either DMSO or lorlatinib (100 nM). Data from data file S4 were used to generate Figure 2H. Data file is in the online supplementary materials as an ‘.xlsx’ file.
