# Supplementary material for: Targeting ERBB3 and AKT to overcome adaptive resistance in EML4-ALK-driven non-small cell lung cancer
Source: Cell Death Dis. 2024 Dec 18;15(12):912. doi: 10.1038/s41419-024-07272-7 (PMC11655848; doi:10.1038/s41419-024-07272-7)

A

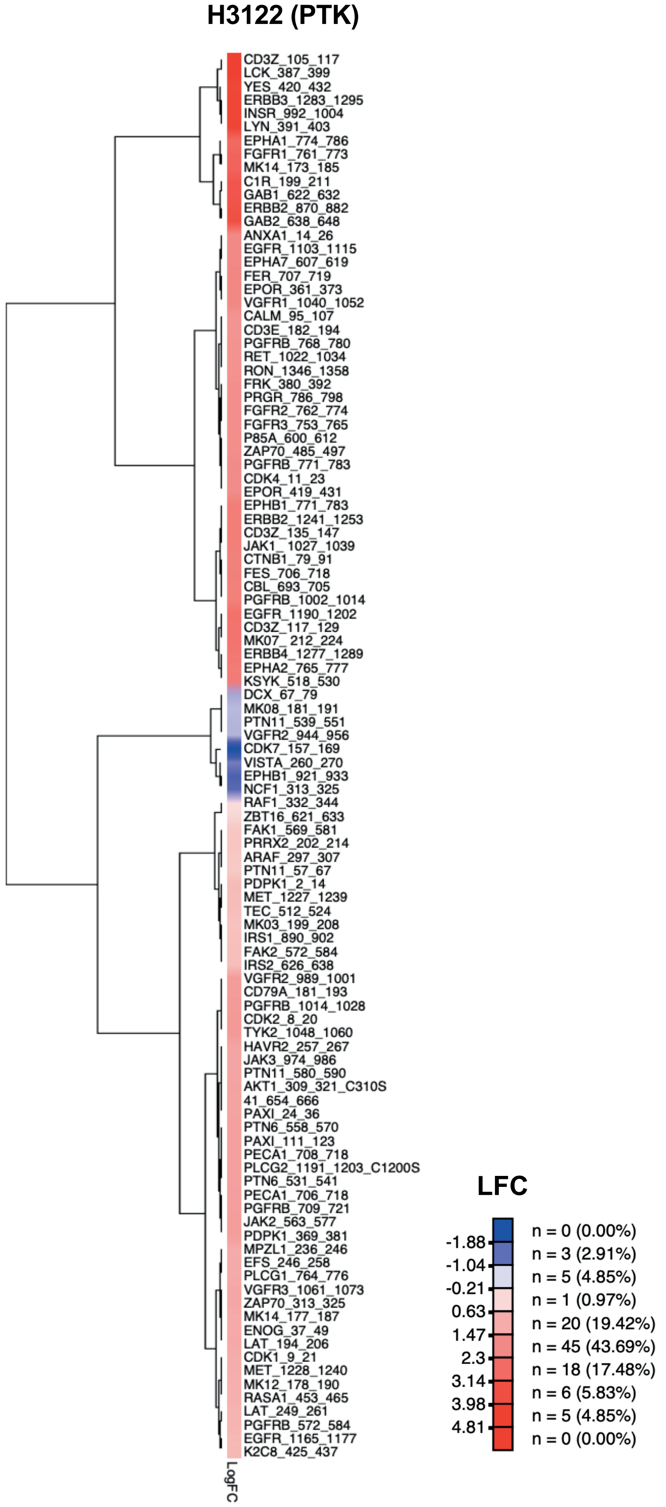

B

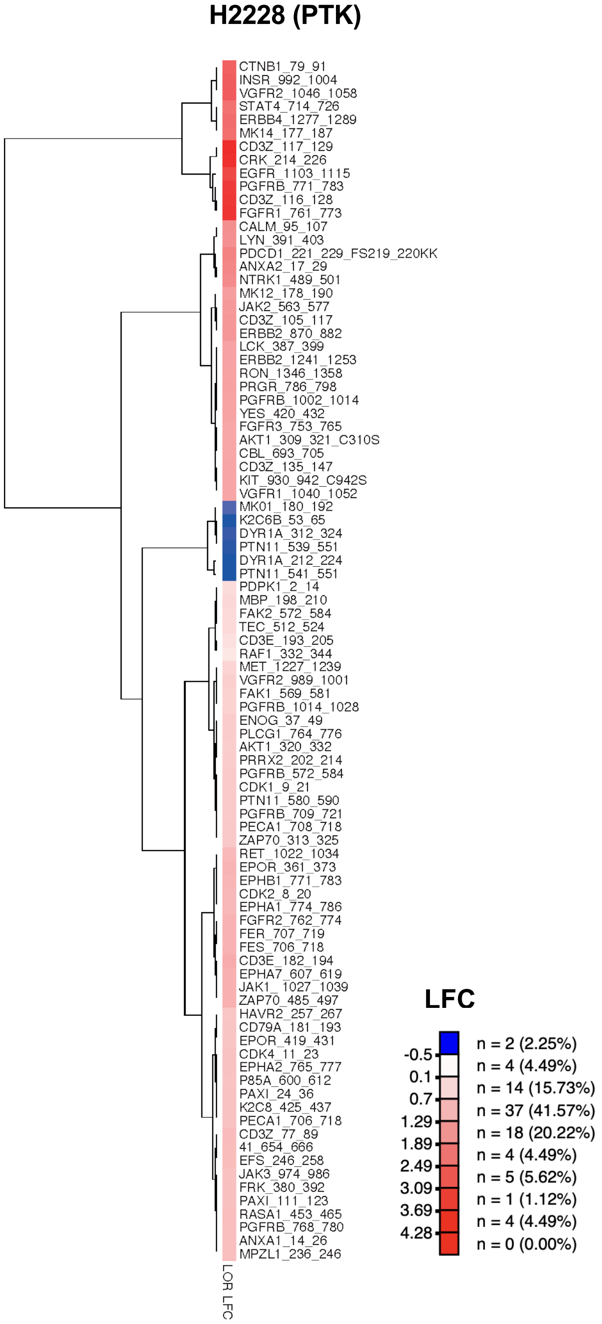

C

H3122

Protein ID	UniProt	LogFoldChange (LFC)
CDK7_157_169	P50613	-1.879466891
EPHB1_921_933	P54762	-1.15942204
NCF1_313_325	P14598	-1.08339417
VSIR_260_270	Q9H7M9	-0.882885754
DCX_67_79	O43602	-0.548716068
VGFR2_944_956	P35968	-0.468338817
PTPN11_539_551	Q06124	-0.451090187
MAPK8_181_191	P45983	-0.415050358

D

H2228

Protein ID	UniProt	LogFoldChange (LFC)
DYR1A_212_224	Q13627	-1.3214762
PTPN11_541_551	Q06124	-0.8958717
K2C6B_53_65	P04259	-0.4723465
PTPN11_539_551	Q06124	-0.423555
DYR1A_312_324	Q13627	-0.3770512
MAPK1_180_192	P28482	-0.2987693

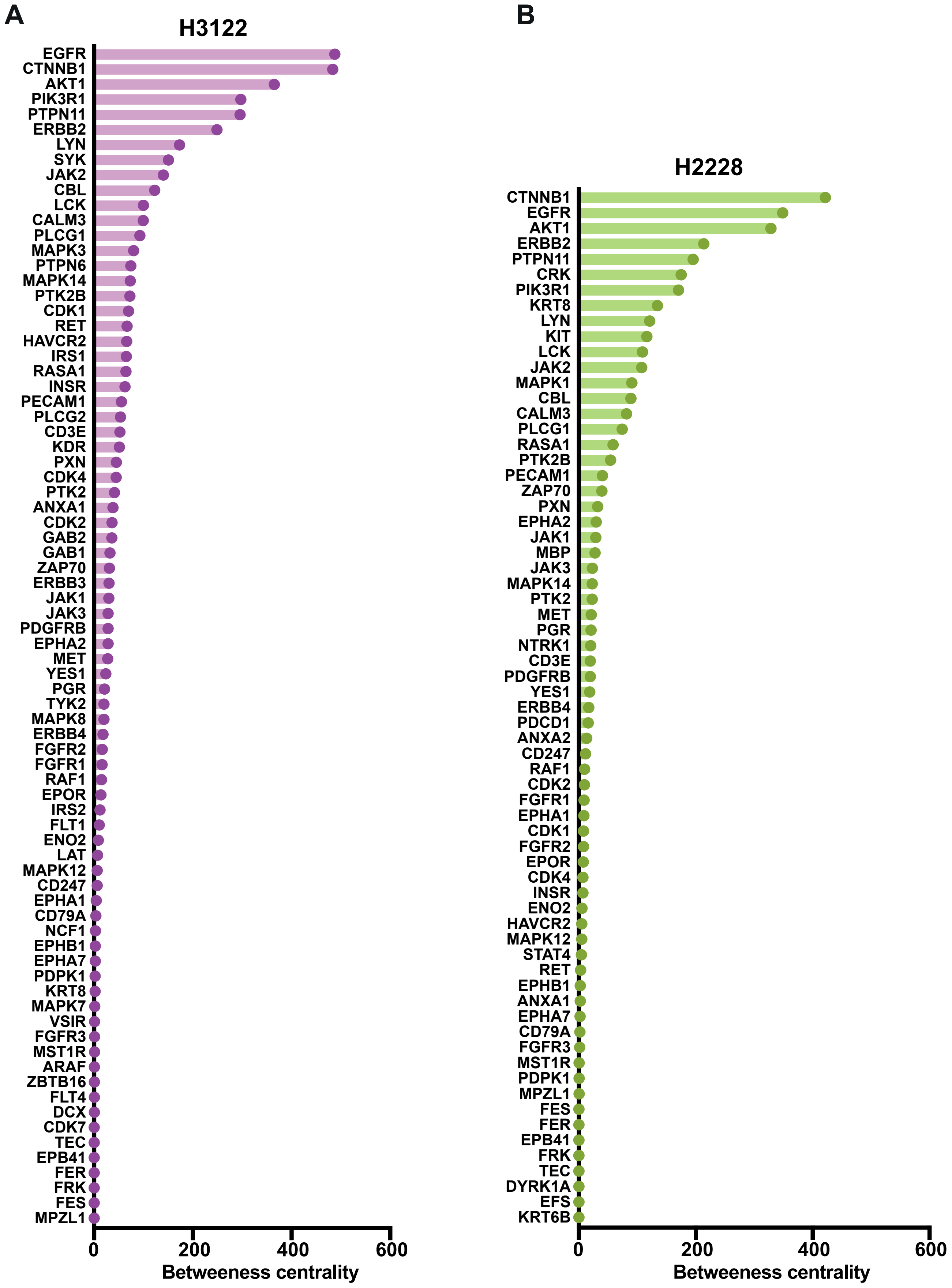
A
H3122
EGFR
CTNNB1
AKT1
PIK3R1
PTPN11
ERBB2
LYN
SYK
JAK2
CBL
LCK
CALM3
PLCG1
MAPK3
PTPN6
MAPK14
PTK2B
CDK1
RET
HAVCR2
IRS1
RASA1
INSR
PECAM1
PLCG2
CD3E
KDR
PXN
CDK4
PTK2
ANXA1
CDK2
GAB2
GAB1
ZAP70
ERBB3
JAK1
JAK3
PDGFRB
EPHA2
MET
YES1
PGR
TYK2
MAPK8
ERBB4
FGFR2
FGFR1
RAF1
EPOR
IRS2
FLT1
ENO2
LAT
MAPK12
CD247
EPHA1
CD79A
NCF1
EPHB1
EPHA7
PDPK1
KRT8
MAPK7
VSIR
FGFR3
MST1R
ARAF
ZBTB16
FLT4
DCX
CDK7
TEC
EPB41
FER
FRK
FES
MPZL1
0
200
400
600
Betweeness centrality
B
H2228
CTNNB1
EGFR
AKT1
ERBB2
PTPN11
CRK
PIK3R1
KRT8
LYN
KIT
LCK
JAK2
MAPK1
CBL
CALM3
PLCG1
RASA1
PTK2B
PECAM1
ZAP70
PXN
EPHA2
JAK1
MBP
JAK3
MAPK14
PTK2
MET
PGR
NTRK1
CD3E
PDGFRB
YES1
ERBB4
PDCD1
ANXA2
CD247
RAF1
CDK2
FGFR1
EPHA1
CDK1
FGFR2
EPOR
CDK4
INSR
ENO2
HAVCR2
MAPK12
STAT4
RET
EPHB1
ANXA1
EPHA7
CD79A
FGFR3
MST1R
PDPK1
MPZL1
FES
FER
EPB41
FRK
TEC
DYRK1A
EFS
KRT6B
0
200
400
600
Betweeness centrality

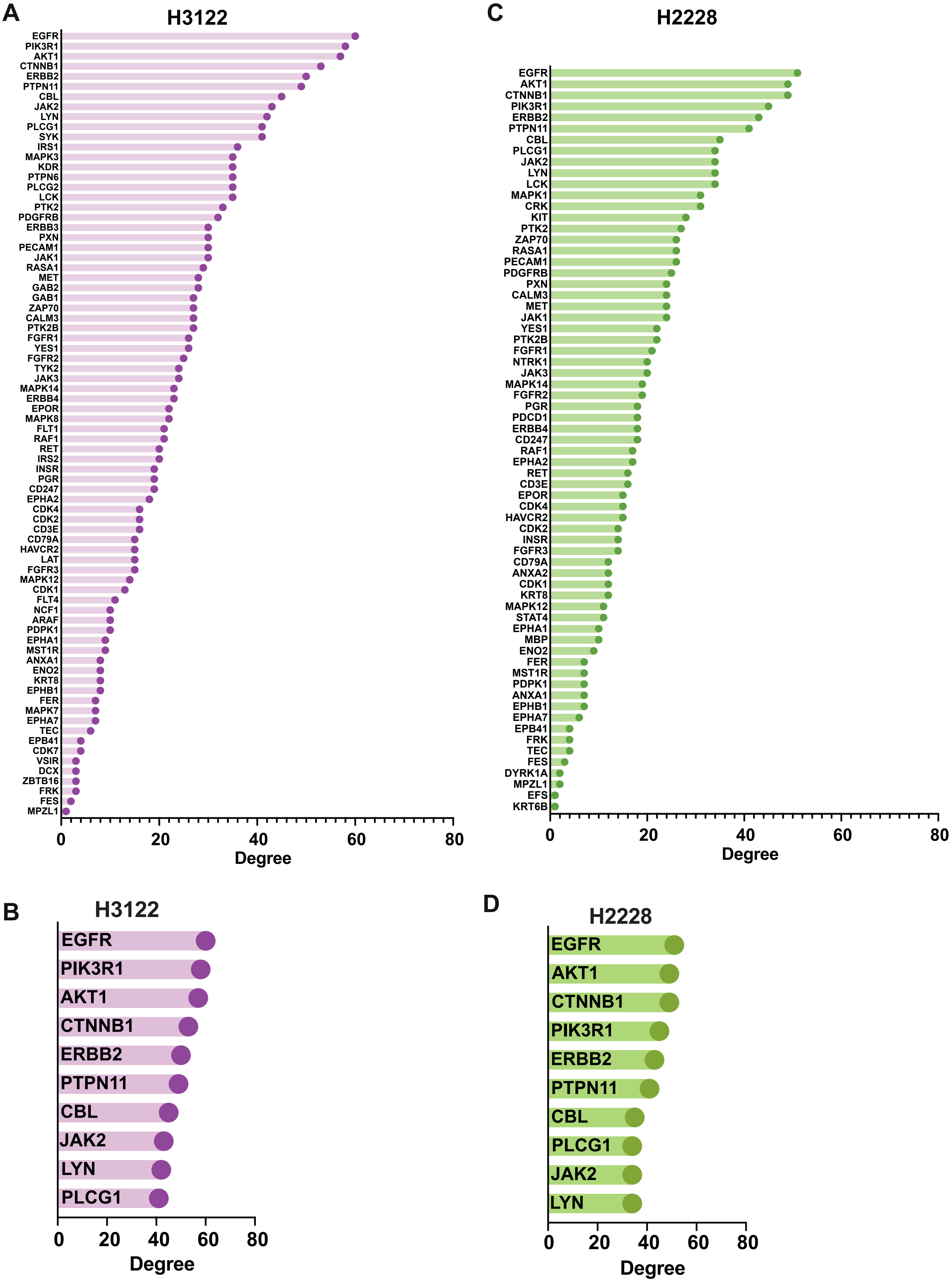
A
H3122
EGFR
PIK3R1
AKT1
CTNNB1
ERBB2
PTPN11
CBL
JAK2
LYN
PLCG1
SYK
IRS1
MAPK3
KDR
PTPN6
PLCG2
LCK
PTK2
PDGFRB
ERBB3
PXN
PECAM1
JAK1
RASA1
MET
GAB2
GAB1
ZAP70
CALM3
PTK2B
FGFR1
YES1
FGFR2
TYK2
JAK3
MAPK14
ERBB4
EPOR
MAPK8
FLT1
RAF1
RET
IRS2
INSR
PGR
CD247
EPHA2
CDK4
CDK2
CD3E
CD79A
HAVCR2
LAT
FGFR3
MAPK12
CDK1
FLT4
NCF1
ARAF
PDPK1
EPHA1
MST1R
ANXA1
ENO2
KRT8
EPHB1
FER
MAPK7
EPHA7
TEC
EPB41
CDK7
VSIR
DCX
ZBTB16
FRK
FES
MPZL1
0
20
40
60
80
Degree
C
H2228
EGFR
AKT1
CTNNB1
PIK3R1
ERBB2
PTPN11
CBL
PLCG1
JAK2
LYN
LCK
MAPK1
CRK
KIT
PTK2
ZAP70
RASA1
PECAM1
PDGFRB
PXN
CALM3
MET
JAK1
YES1
PTK2B
FGFR1
NTRK1
JAK3
MAPK14
FGFR2
PGR
PDCD1
ERBB4
CD247
RAF1
EPHA2
RET
CD3E
EPOR
CDK4
HAVCR2
CDK2
INSR
FGFR3
CD79A
ANXA2
CDK1
KRT8
MAPK12
STAT4
EPHA1
MBP
ENO2
FER
MST1R
PDPK1
ANXA1
EPHB1
EPHA7
EPB41
FRK
TEC
FES
DYRK1A
MPZL1
EFS
KRT6B
0
20
40
60
80
Degree
B
H3122
EGFR
PIK3R1
AKT1
CTNNB1
ERBB2
PTPN11
CBL
JAK2
LYN
PLCG1
0
20
40
60
80
Degree
D
H2228
EGFR
AKT1
CTNNB1
PIK3R1
ERBB2
PTPN11
CBL
PLCG1
JAK2
LYN
0
20
40
60
80
Degree

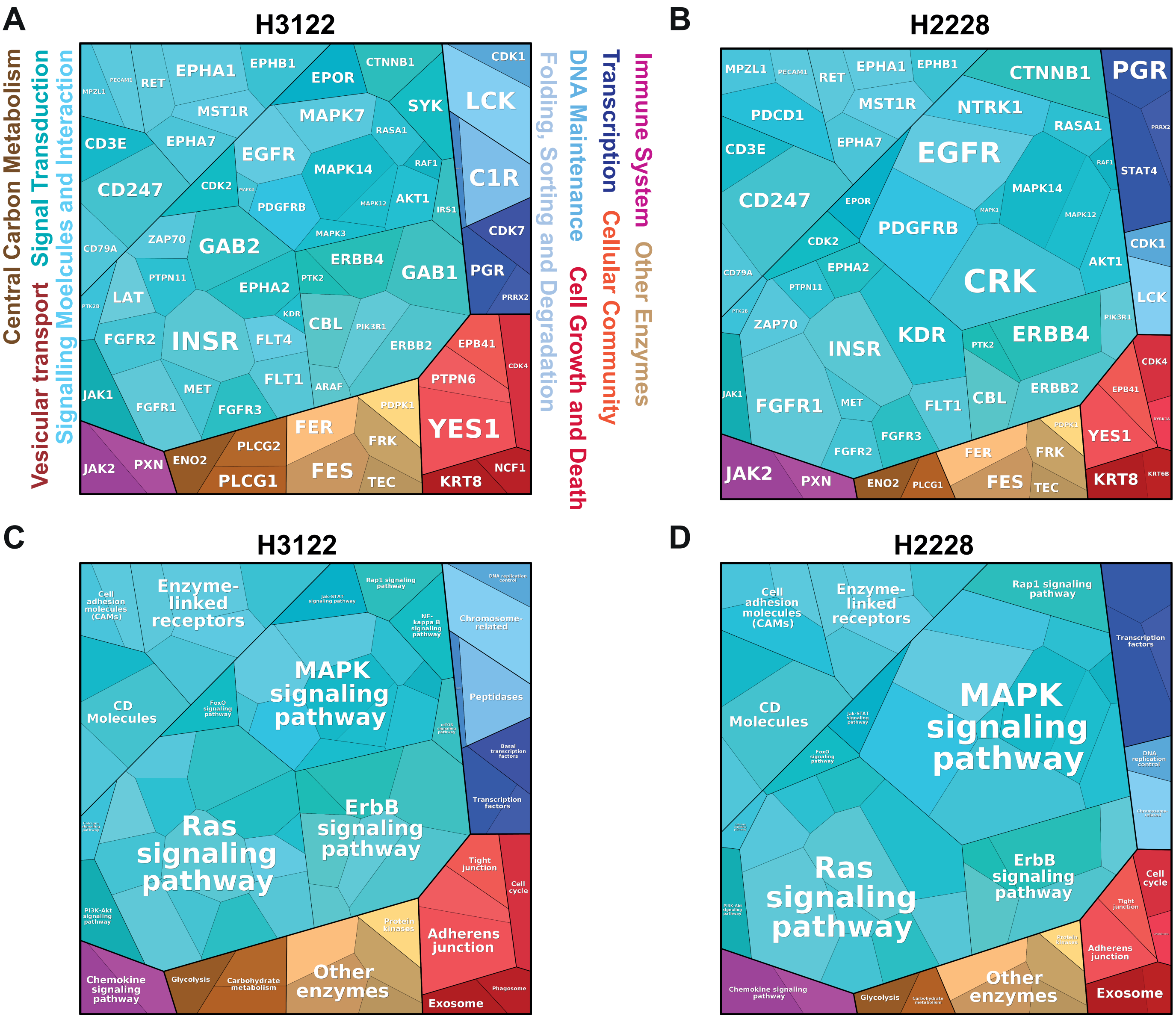

E

ERL+LOR-H3122vsH2228 (Two-way ANOVA)

Difference between column means	Value	P value
Mean of ERL+LOR (3.12 nM)-H3122	89.46	
Mean of ERL+LOR (3.12 nM)-H2228	76.32	
Difference between means	13.14	
SE of difference	7.576	
95% CI of difference	-10.97 to 37.25	
Row Factor		<0.0001 (****)
Row Factor x Column Factor		0.0385 (*)
Column Factor		0.1813 (ns)

F

SAP+LOR-H3122 (Two-way ANOVA)

Difference between column means	Value	P value
Mean of SAP-H3122	91.10	
Mean of SAP+LOR (3.12 nM)-H3122	61.04	
Difference between means	30.06	
SE of difference	3.237	
95% CI of difference	16.14 to 43.99	
Row Factor		<0.0001 (****)
Row Factor x Column Factor		<0.0001 (****)
Column Factor		0.0114 (*)

SAP+LOR-H2228 (Two-way ANOVA)

Difference between column means	Value	P value
Mean of SAP-H2228	104.6	
Mean of SAP+LOR (3.12 nM)-H2228	70.31	
Difference between means	34.33	
SE of difference	1.516	
95% CI of difference	29.15 to 39.16	
Row Factor		<0.0001 (****)
Row Factor x Column Factor		<0.0001 (****)
Column Factor		0.0002 (****)

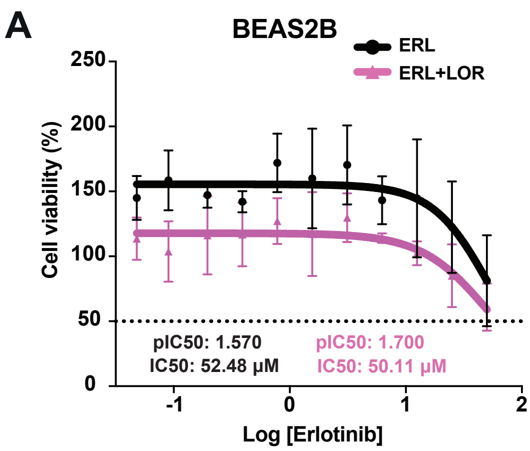

ERLvsERL+LOR-BEAS2B (Two-way ANOVA)

Difference between column means	Value	P value
Mean of ERL	140.5	
Mean of ERL+LOR (3.12 nM)	107.2	
Difference between means	33.31	
SE of difference	6.027	
95% CI of difference	14.13 to 52.49	
Row Factor		<0.0001 (****)
Row Factor x Column Factor		0.0117 (*)
Column Factor		0.5802 (ns)

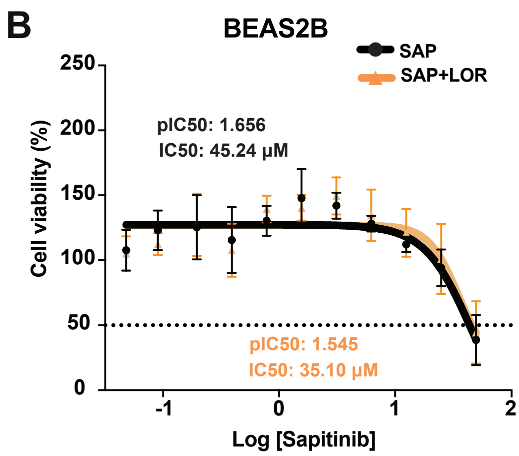

SAPvsSAP+LOR-BEAS2B (Two-way ANOVA)

Difference between column means	Value	P value
Mean of SAP	113.8	
Mean of SAP+LOR (3.12 nM)	115.3	
Difference between means	-1.494	
SE of difference	4.675	
95% CI of difference	-16.37 to 13.38	
Row Factor		<0.0001 (****)
Row Factor x Column Factor		0.7702 (ns)
Column Factor		0.1045 (ns)

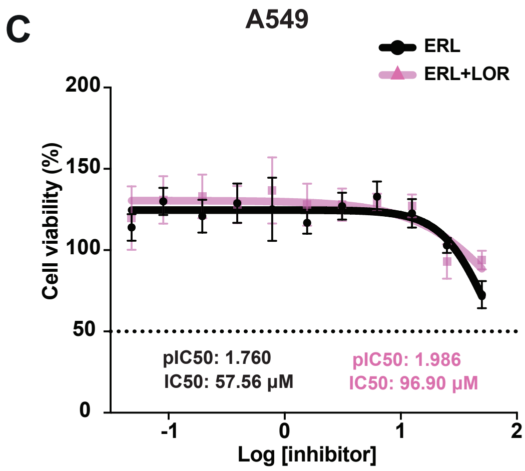

ERLvsERL+LOR-A549 (Two-way ANOVA)

Difference between column means	Value	P value
Mean of ERL	116.1	
Mean of ERL+LOR (3.12 nM)	120.7	
Difference between means	-4.514	
SE of difference	3.578	
95% CI of difference	-15.90 to 6.874	
Row Factor		<0.0001 (****)
Row Factor x Column Factor		0.2963 (ns)
Column Factor		0.2683 (ns)

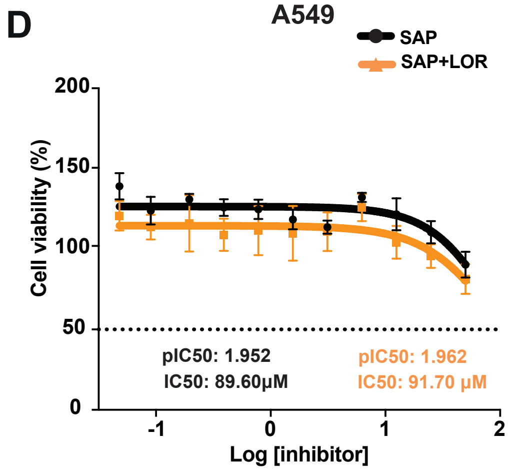

SAPvsSAP+LOR-A549 (Two-way ANOVA)

Difference between column means	Value	P value
Mean of SAP	119.2	
Mean of SAP+LOR (3.12 nM)	108.2	
Difference between means	11.05	
SE of difference	3.922	
95% CI of difference	-1.431 to 23.53	
Row Factor		<0.0001 (****)
Row Factor x Column Factor		0.0669 (ns)
Column Factor		0.0792 (ns)

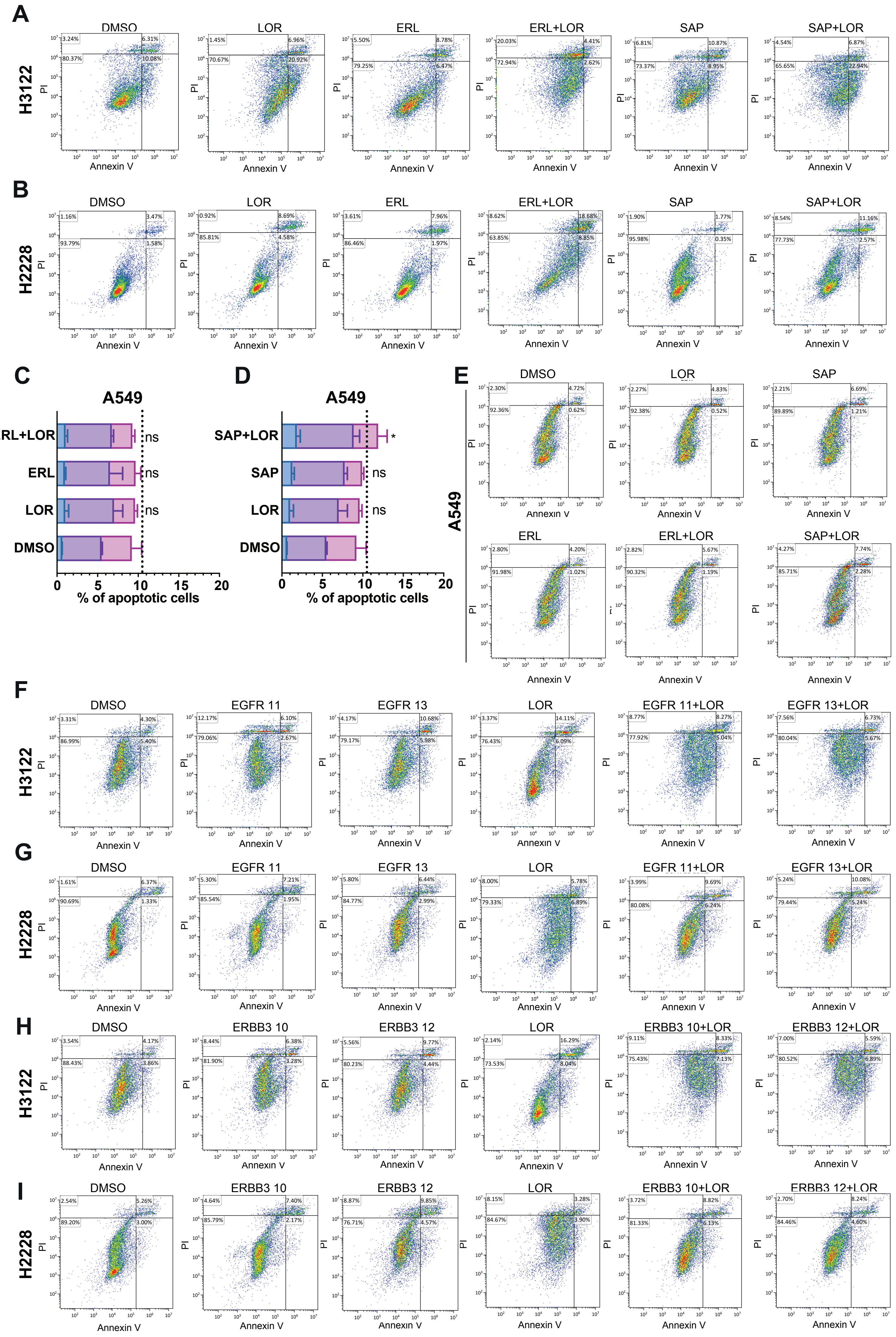
A
H3122
DMSO
3.24%
6.31%
80.37%
10.08%
LOR
1.45%
6.96%
70.67%
20.92%
ERL
5.50%
8.78%
79.25%
6.47%
ERL+LOR
20.03%
4.41%
72.94%
2.62%
SAP
6.81%
10.87%
73.37%
8.95%
SAP+LOR
4.54%
6.87%
65.65%
22.94%
PI
Annexin V
B
H2228
DMSO
1.16%
3.47%
93.79%
1.58%
LOR
0.92%
8.69%
85.81%
4.58%
ERL
3.61%
7.96%
86.46%
1.97%
ERL+LOR
8.62%
18.68%
63.85%
8.85%
SAP
1.90%
1.77%
95.98%
0.35%
SAP+LOR
8.54%
11.16%
77.73%
2.57%
C
A549
ERL+LOR
ERL
LOR
DMSO
ns
% of apoptotic cells
D
A549
SAP+LOR
SAP
LOR
DMSO
*
ns
% of apoptotic cells
E
A549
DMSO
2.30%
4.72%
92.36%
0.62%
LOR
2.27%
4.83%
92.38%
0.52%
SAP
2.21%
6.69%
89.89%
1.21%
ERL
2.80%
4.20%
91.98%
1.02%
ERL+LOR
2.82%
5.67%
90.32%
1.19%
SAP+LOR
4.27%
7.74%
85.71%
2.28%
F
H3122
DMSO
3.31%
4.30%
86.99%
5.40%
EGFR 11
12.17%
6.10%
79.06%
2.67%
EGFR 13
4.17%
10.68%
79.17%
5.98%
LOR
3.37%
14.11%
76.43%
6.09%
EGFR 11+LOR
8.77%
8.27%
77.92%
5.04%
EGFR 13+LOR
7.56%
6.73%
80.04%
5.67%
G
H2228
DMSO
1.61%
6.37%
90.69%
1.33%
EGFR 11
5.30%
7.21%
85.54%
1.95%
EGFR 13
5.80%
6.44%
84.77%
2.99%
LOR
8.00%
5.78%
79.33%
6.89%
EGFR 11+LOR
3.99%
9.69%
80.08%
6.24%
EGFR 13+LOR
5.24%
10.08%
79.44%
5.24%
H
H3122
DMSO
3.54%
4.17%
88.43%
3.86%
ERBB3 10
8.44%
6.38%
81.90%
3.28%
ERBB3 12
5.56%
9.77%
80.23%
4.44%
LOR
2.14%
16.29%
73.53%
8.04%
ERBB3 10+LOR
9.11%
8.33%
75.43%
7.13%
ERBB3 12+LOR
7.00%
5.59%
80.52%
6.89%
I
H2228
DMSO
2.54%
5.26%
89.20%
3.00%
ERBB3 10
4.64%
7.40%
85.79%
2.17%
ERBB3 12
8.87%
9.85%
76.71%
4.57%
LOR
8.15%
3.28%
84.67%
3.90%
ERBB3 10+LOR
3.72%
8.82%
81.33%
6.13%
ERBB3 12+LOR
2.70%
8.24%
84.46%
4.60%

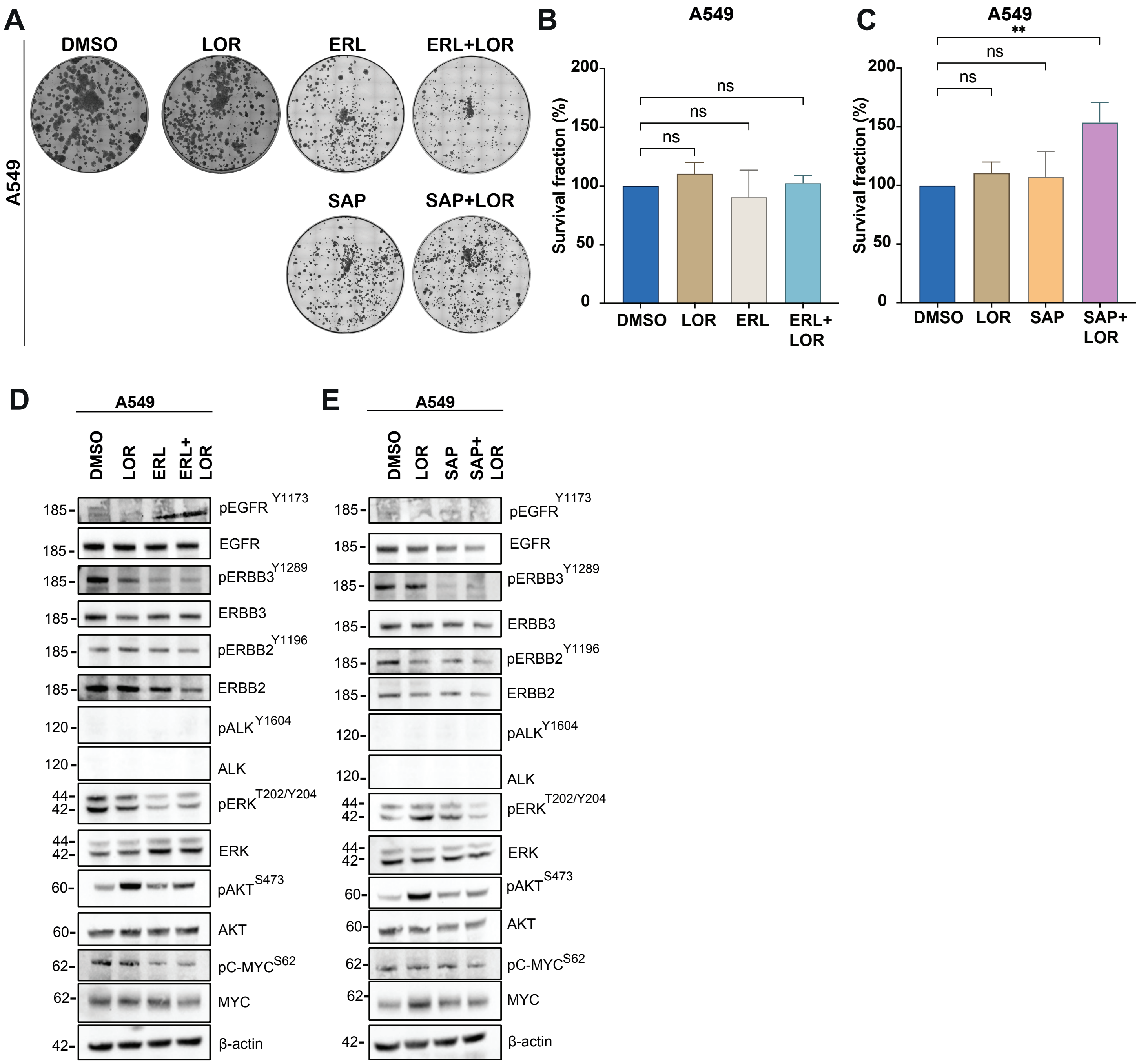
A
DMSO
LOR
ERL
ERL+LOR
SAP
SAP+LOR
A549
B
A549
Survival fraction (%)
200
150
100
50
0
ns
ns
ns
DMSO
LOR
ERL
ERL+ LOR
C
A549
**
ns
ns
Survival fraction (%)
200
150
100
50
0
DMSO
LOR
SAP
SAP+ LOR
D
A549
DMSO
LOR
ERL
ERL+ LOR
185
pEGFR Y1173
185
EGFR
185
pERBB3 Y1289
185
ERBB3
185
pERBB2 Y1196
185
ERBB2
120
pALK Y1604
120
ALK
44
42
pERK T202/Y204
44
42
ERK
60
pAKT S473
60
AKT
62
pC-MYC S62
62
MYC
42
β-actin
E
A549
DMSO
LOR
SAP
SAP+ LOR
185
pEGFR Y1173
185
EGFR
185
pERBB3 Y1289
185
ERBB3
185
pERBB2 Y1196
185
ERBB2
120
pALK Y1604
120
ALK
44
42
pERK T202/Y204
44
42
ERK
60
pAKT S473
60
AKT
62
pC-MYC S62
62
MYC
42
β-actin

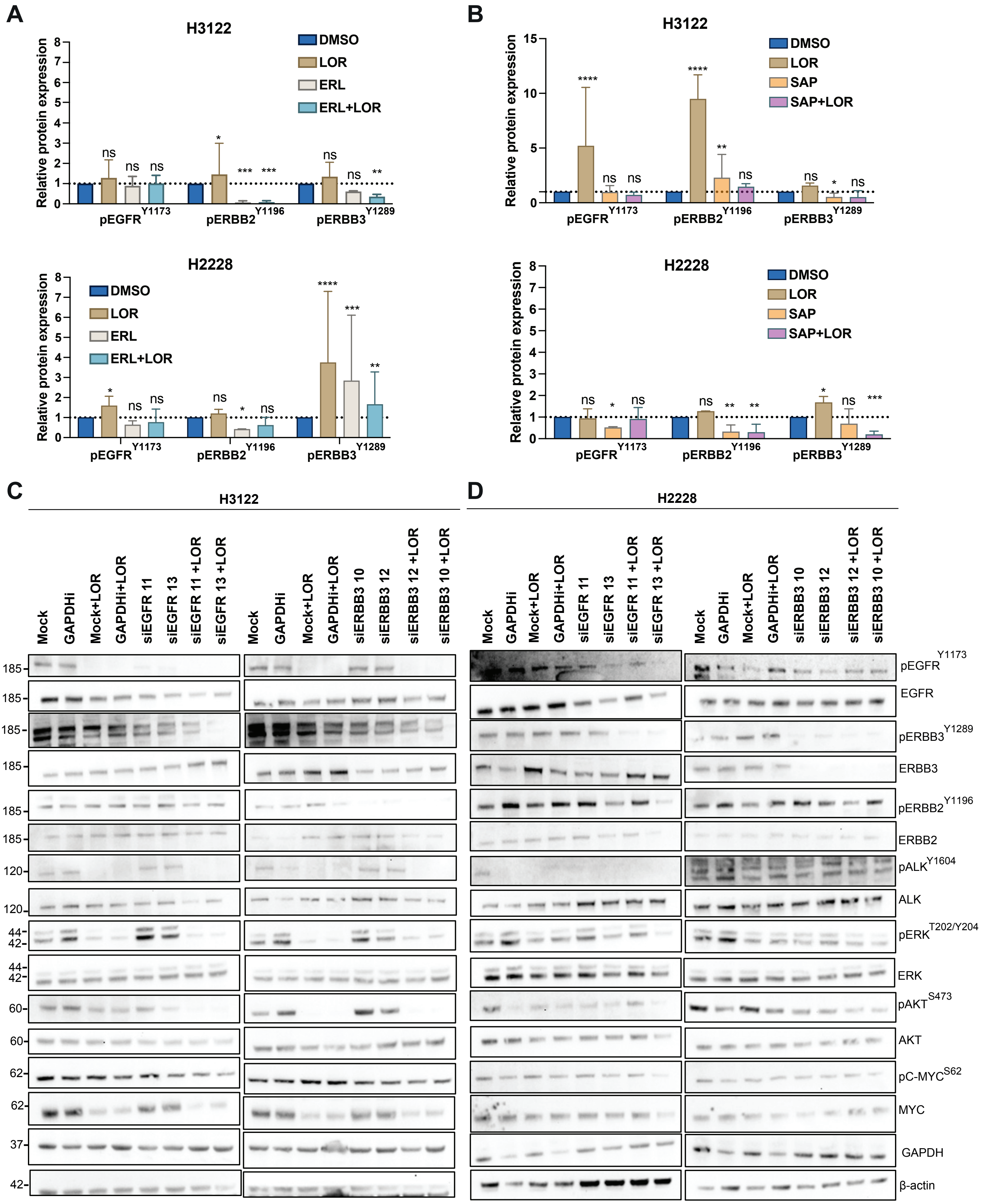

A
H3122
Relative protein expression
DMSO
LOR
ERL
ERL+LOR
pEGFR Y1173
pERBB2 Y1196
pERBB3 Y1289
H2228
B
SAP
SAP+LOR
C
Mock
GAPDHi
Mock+LOR
GAPDHi+LOR
siEGFR 11
siEGFR 13
siEGFR 11 +LOR
siEGFR 13 +LOR
siERBB3 10
siERBB3 12
siERBB3 12 +LOR
siERBB3 10 +LOR
185
120
44
42
60
62
37
D
pEGFR Y1173
EGFR
pERBB3 Y1289
ERBB3
pERBB2 Y1196
ERBB2
pALK Y1604
ALK
pERK T202/Y204
ERK
pAKT S473
AKT
pC-MYC S62
MYC
GAPDH
β-actin

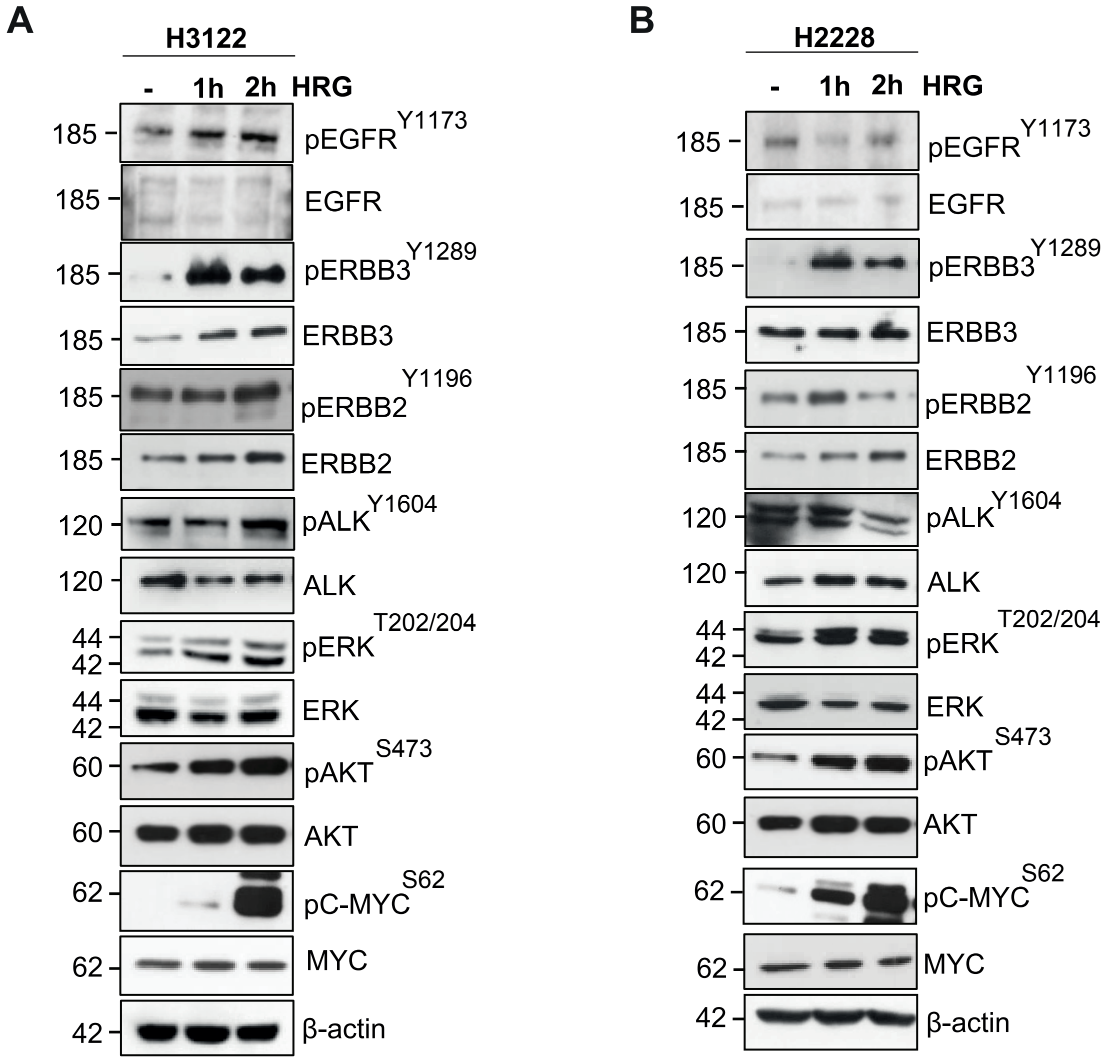

A
H3122
- 1h 2h HRG
185 pEGFR Y1173
185 EGFR
185 pERBB3 Y1289
185 ERBB3
185 pERBB2 Y1196
185 ERBB2
120 pALK Y1604
120 ALK
44 42 pERK T202/204
44 42 ERK
60 pAKT S473
60 AKT
62 pC-MYC S62
62 MYC
42 β-actin
B
H2228
- 1h 2h HRG
185 pEGFR Y1173
185 EGFR
185 pERBB3 Y1289
185 ERBB3
185 pERBB2 Y1196
185 ERBB2
120 pALK Y1604
120 ALK
44 42 pERK T202/204
44 42 ERK
60 pAKT S473
60 AKT
62 pC-MYC S62
62 MYC
42 β-actin

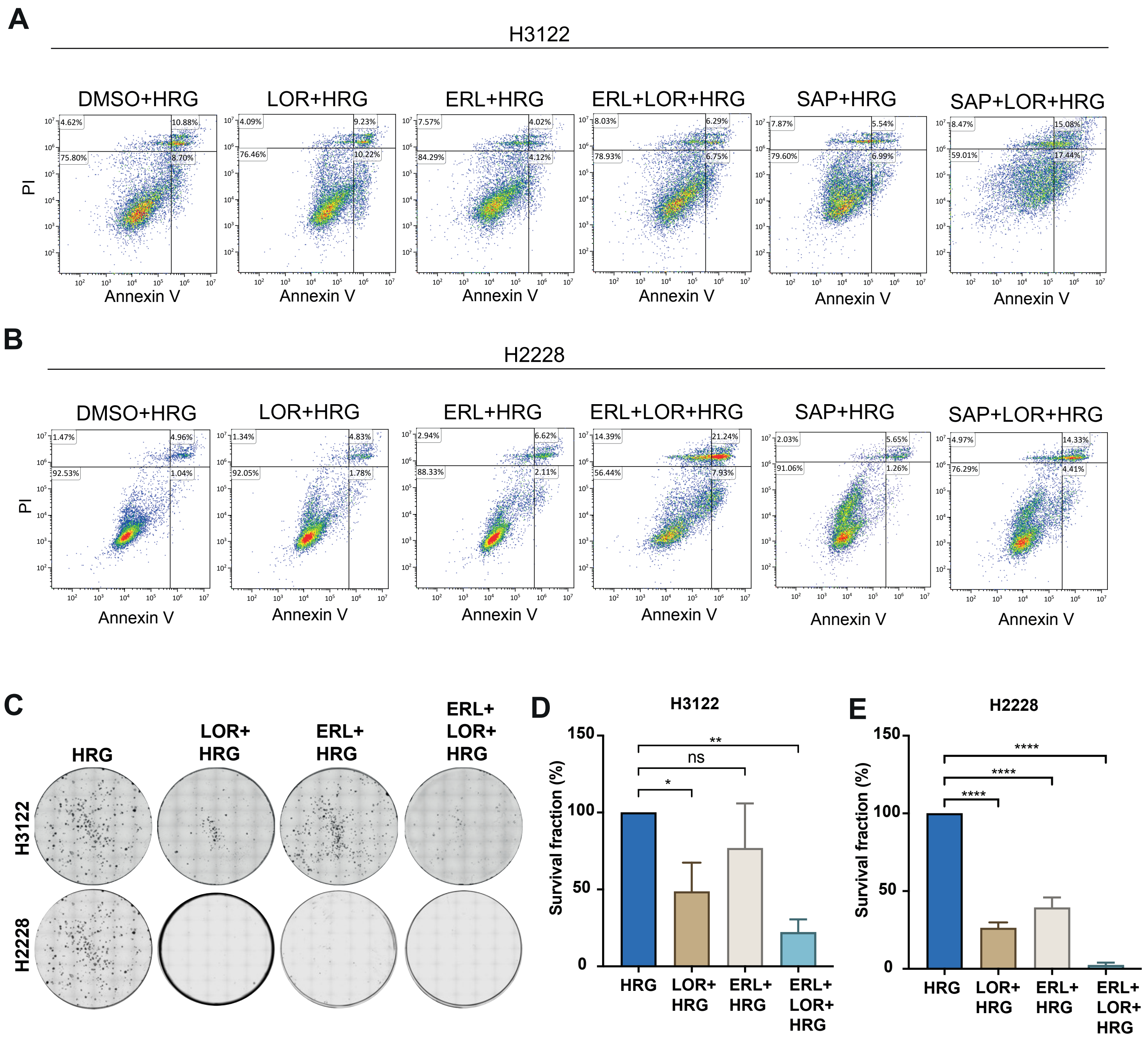
A
H3122
DMSO+HRG
LOR+HRG
ERL+HRG
ERL+LOR+HRG
SAP+HRG
SAP+LOR+HRG
PI
Annexin V
4.62%
10.88%
75.80%
8.70%
4.09%
9.23%
76.46%
10.22%
7.57%
4.02%
84.29%
4.12%
8.03%
6.29%
78.93%
6.75%
7.87%
5.54%
79.60%
6.99%
8.47%
15.08%
59.01%
17.44%
B
H2228
1.47%
4.96%
92.53%
1.04%
1.34%
4.83%
92.05%
1.78%
2.94%
6.62%
88.33%
2.11%
14.39%
21.24%
56.44%
7.93%
2.03%
5.65%
91.06%
1.26%
4.97%
14.33%
76.29%
4.41%
C
HRG
LOR+ HRG
ERL+ HRG
ERL+ LOR+ HRG
H3122
H2228
D
H3122
Survival fraction (%)
150
100
50
0
**
ns
*
HRG
LOR+ HRG
ERL+ HRG
ERL+ LOR+ HRG
E
H2228
Survival fraction (%)
150
100
50
0
****
****
****
HRG
LOR+ HRG
ERL+ HRG
ERL+ LOR+ HRG

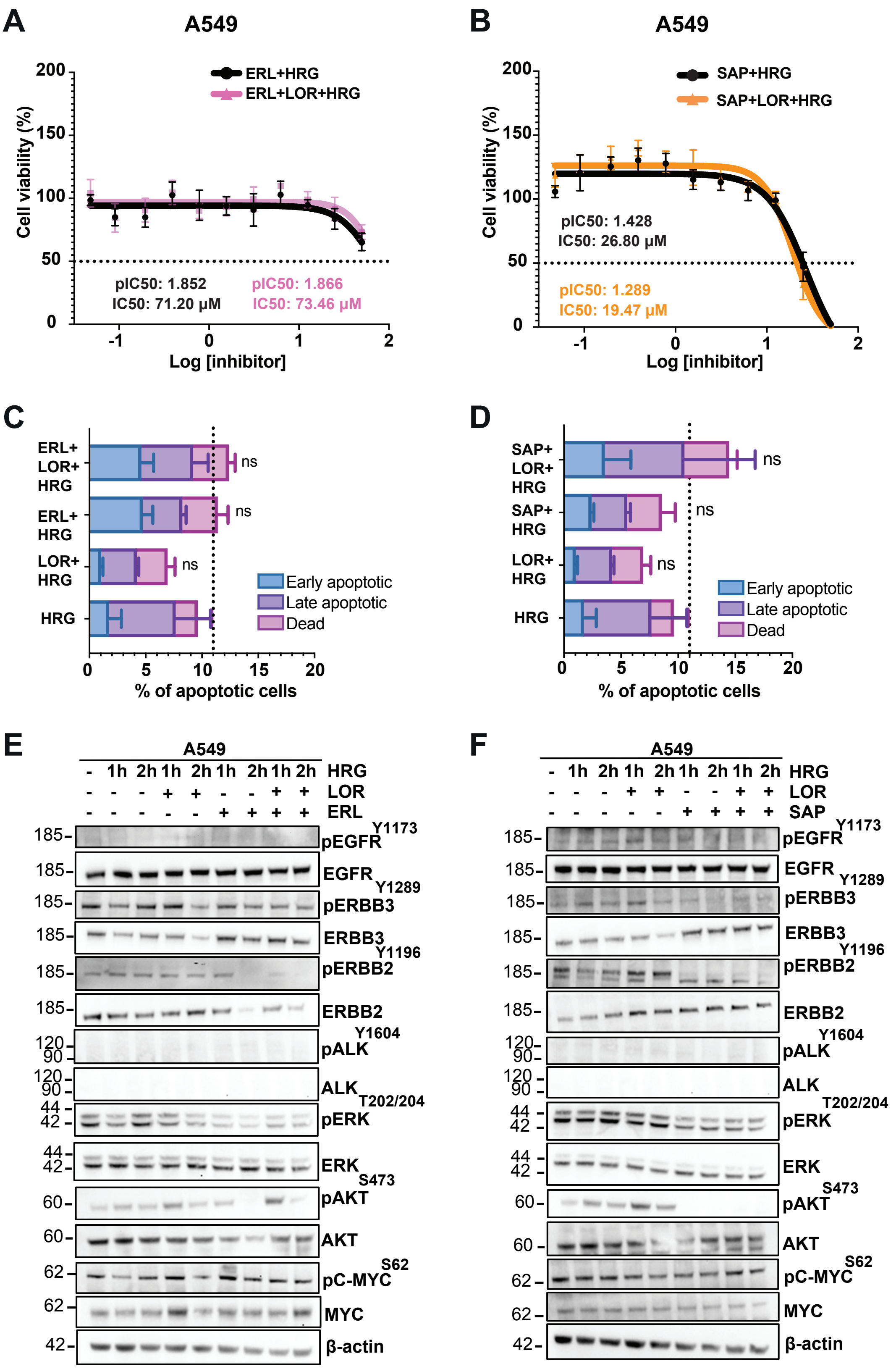
A
A549
ERL+HRG
ERL+LOR+HRG
Cell viability (%)
200
150
100
50
0
-1
0
1
2
Log [inhibitor]
pIC50: 1.852
IC50: 71.20 µM
pIC50: 1.866
IC50: 73.46 µM
B
A549
SAP+HRG
SAP+LOR+HRG
Cell viability (%)
pIC50: 1.428
IC50: 26.80 µM
pIC50: 1.289
IC50: 19.47 µM
Log [inhibitor]
C
ERL+ LOR+ HRG
ERL+ HRG
LOR+ HRG
HRG
ns
Early apoptotic
Late apoptotic
Dead
0
5
10
15
20
% of apoptotic cells
D
SAP+ LOR+ HRG
SAP+ HRG
LOR+ HRG
HRG
ns
Early apoptotic
Late apoptotic
Dead
% of apoptotic cells
E
A549
- 1h 2h 1h 2h 1h 2h 1h 2h HRG
- - - + + - - + + LOR
- - - - - + + + + ERL
185 pEGFR Y1173
185 EGFR
185 pERBB3 Y1289
185 ERBB3
185 pERBB2 Y1196
185 ERBB2
120 90 pALK Y1604
120 90 ALK
44 42 pERK T202/204
44 42 ERK
60 pAKT S473
60 AKT
62 pC-MYC S62
62 MYC
42 β-actin
F
A549
- 1h 2h 1h 2h 1h 2h 1h 2h HRG
- - - + + - - + + LOR
- - - - - + + + + SAP
185 pEGFR Y1173
185 EGFR
185 pERBB3 Y1289
185 ERBB3
185 pERBB2 Y1196
185 ERBB2
120 90 pALK Y1604
120 90 ALK
44 42 pERK T202/204
44 42 ERK
60 pAKT S473
60 AKT
62 pC-MYC S62
62 MYC
42 β-actin

A

AKT VIIIvsAKT VIII+LOR-H3122 (Two-way ANOVA)

Difference between column means	Value	P value
Mean of AKT VIII	109.5	
Mean of AKT VIII+LOR (3.12 nM)	74.22	
Difference between means	35.31	
SE of difference	4.616	
95% CI of difference	20.62 to 50.00	
Row Factor		<0.0001 (****)
Row Factor x Column Factor		<0.0001 (****)
Column Factor		0.0046 (**)

B

AKT VIIIvsAKT VIII+LOR-H2228 (Two-way ANOVA)

Difference between column means	Value	P value
Mean of AKT VIII	72.64	
Mean of AKT VIII+LOR (3.12 nM)	77.62	
Difference between means	-4.983	
SE of difference	1.957	
95% CI of difference	-11.21 to 1.245	
Row Factor		<0.0001 (****)
Row Factor x Column Factor		0.0842 (ns)
Column Factor		0.0604 (ns)

C

AKT VIII-H3122vsH2228 (Two-way ANOVA)

Difference between column means	Value	P value
Mean of AKT VIII-H3122	109.5	
Mean of AKT VIII-H2228	72.64	
Difference between means	36.89	
SE of difference	5.281	
95% CI of difference	20.08 to 53.70	
Row Factor		<0.0001 (****)
Row Factor x Column Factor		0.0060 (**)
Column Factor		<0.0001 (****)

D

AKT VIII+LOR-H3122vsH2228 (Two-way ANOVA)

Difference between column means	Value	P value
Mean of AKT VIII+LOR (3.12 nM)-H3122	74.59	
Mean of AKT VIII+LOR (3.12 nM)-H2228	77.62	
Difference between means	5.034	
SE of difference	8.065	
95% CI of difference	-97.44 to 107.5	
Row Factor		<0.0001 (****)
Row Factor x Column Factor		<0.0001 (****)
Column Factor		0.6330 (ns)

E

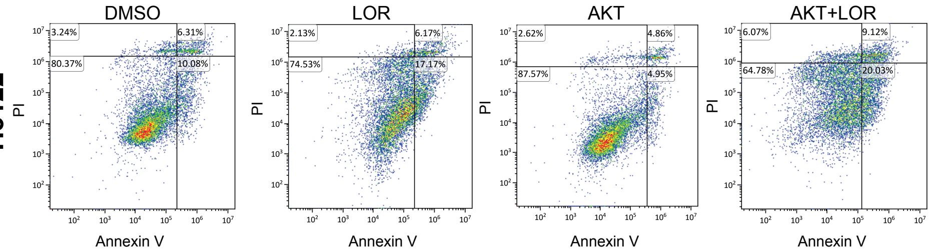

F

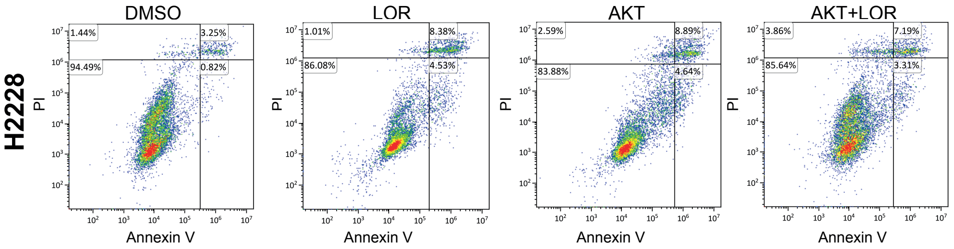

G

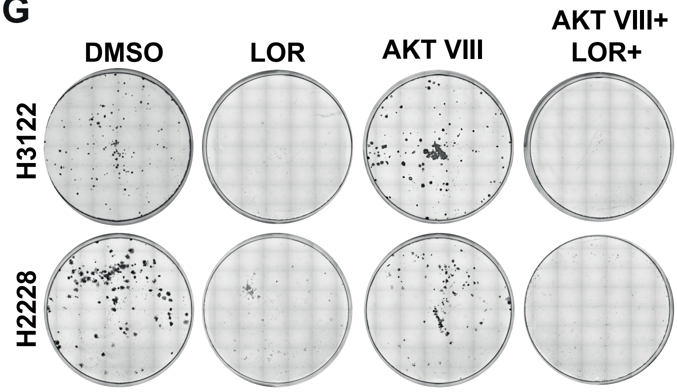

H

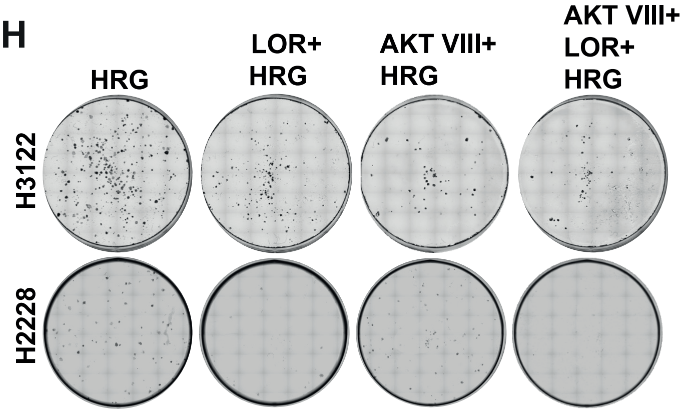

I

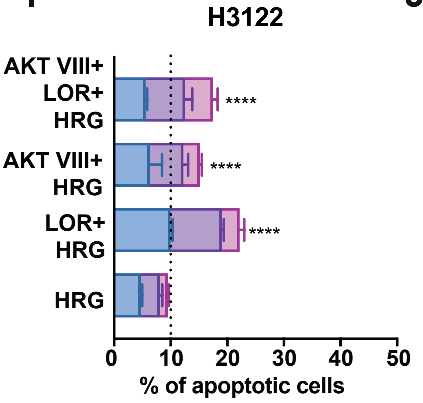

J

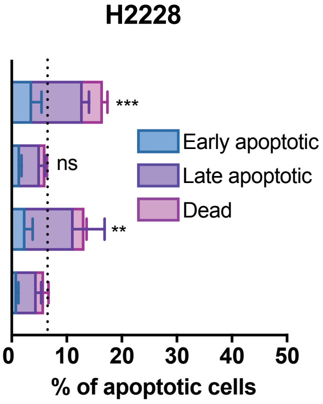

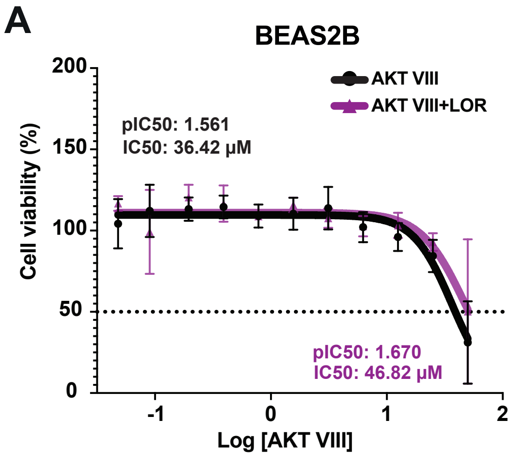

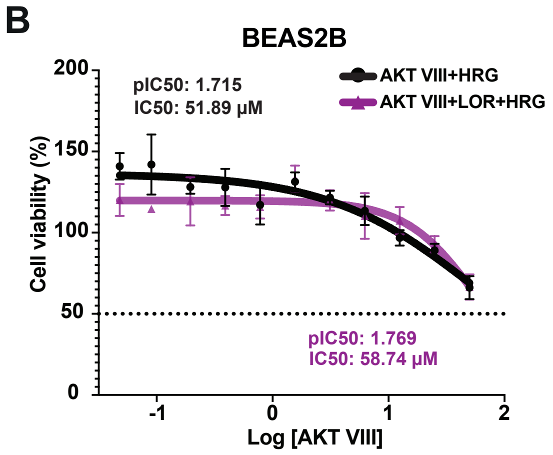

AKT VIIIvsAKT VIII+LOR-BEAS2B (Two-way ANOVA)

Difference between column means	Value	P value
Mean of AKT VIII	99.24	
Mean of AKT VIII+LOR (3.12 nM)	102.9	
Difference between means	-3.612	
SE of difference	3.257	
95% CI of difference	-13.98 to 6.754	
Row Factor		<0.0001 (****)
Row Factor x Column Factor		0.0258 (*)
Column Factor		0.3483 (ns)

AKT VIII+HRGvsAKT VIII+LOR+HRG-BEAS2B (Two-way ANOVA)

Difference between column means	Value	P value
Mean of AKT VIII+HRG	114.5	
Mean of AKT VIII+LOR (3.12 nM)+HRG	109.7	
Difference between means	4.783	
SE of difference	3.992	
95% CI of difference	-7.923 to 17.49	
Row Factor		<0.0001 (****)
Row Factor x Column Factor		0.0012 (**)
Column Factor		0.3169 (ns)

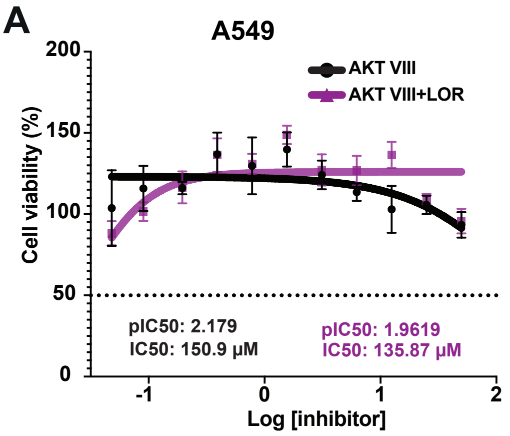

AKT VIIIvsAKT VIII+LOR-A549 (Two-way ANOVA)

Difference between column means	Value	P value
Mean of AKT VIII	115.1	
Mean of AKT VIII+LOR (3.12 nM)	118.1	
Difference between means	-2.999	
SE of difference	5.320	
95% CI of difference	-19.93 to 13.93	
Row Factor		<0.0001 (****)
Row Factor x Column Factor		0.078 (ns)
Column Factor		0.6124 (ns)

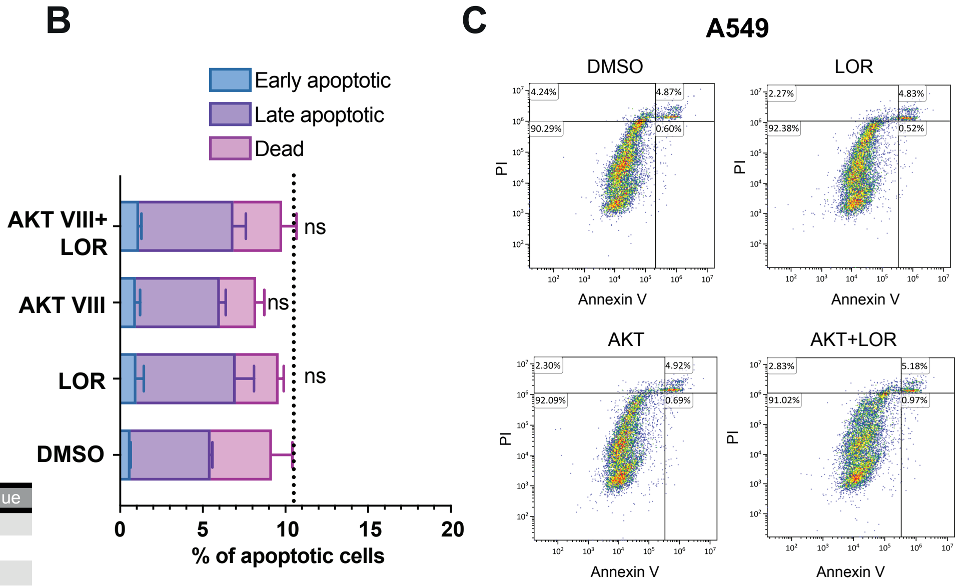

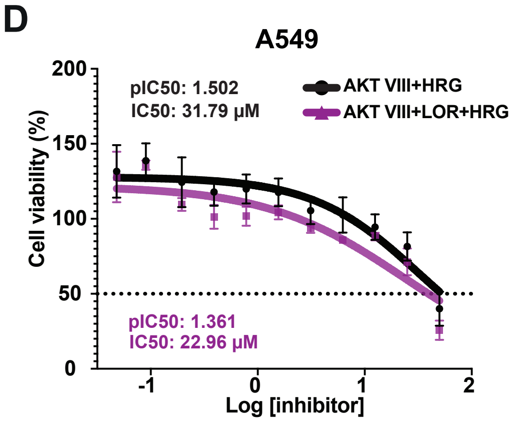

AKT VIII+HRGvsAKT VIII+LOR+HRG-A549 (Two-way ANOVA)

Difference between column means	Value	P value
Mean of AKT VIII+HRG	106.2	
Mean of AKT VIII+LOR (3.12 nM)+HRG	95.42	
Difference between means	10.76	
SE of difference	4.108	
95% CI of difference	-2.315 to 23.83	
Row Factor		<0.0001 (****)
Row Factor x Column Factor		0.4379 (ns)
Column Factor		0.0791(ns)

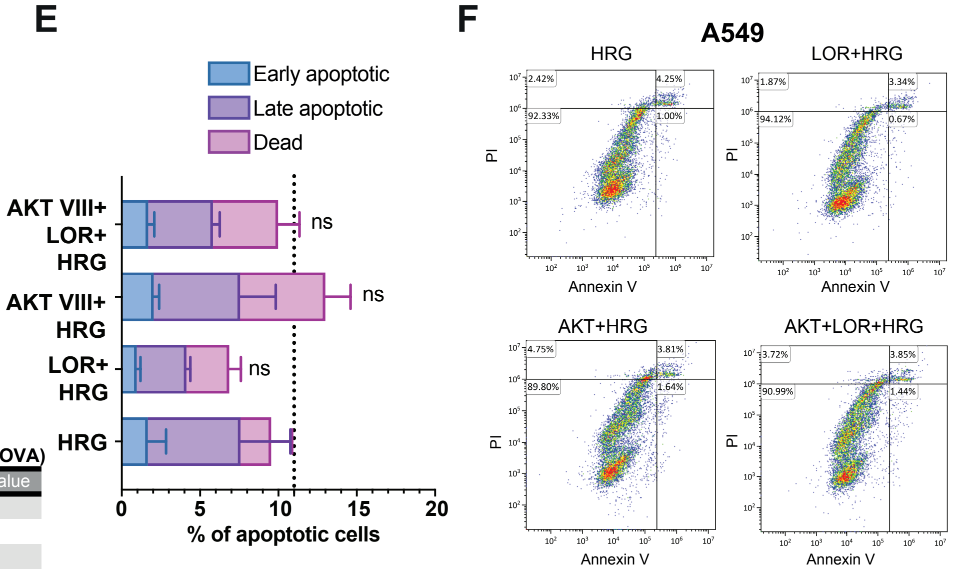

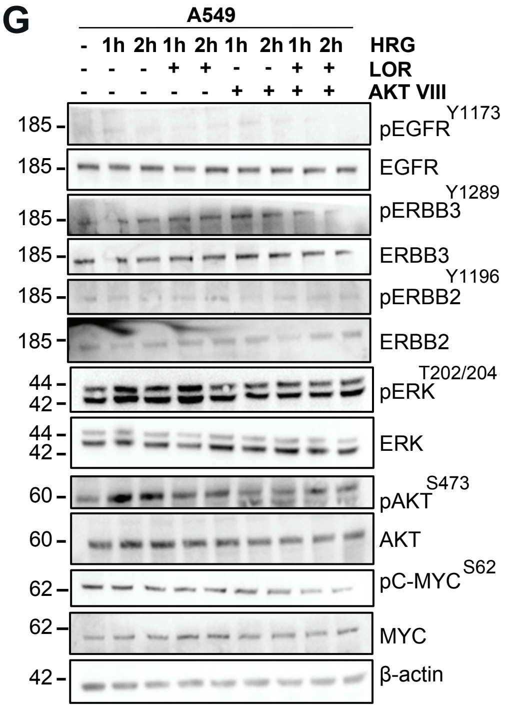

A

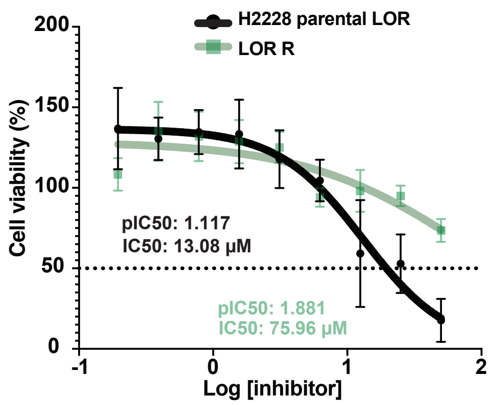

B

Parental LORvsLOR R-H2228 LOR R (Two-way ANOVA)

Difference between column means	Value	P value
Mean of Parental LOR	97.84	
Mean of LOR R	111.5	
Difference between means	-13.61	
SE of difference	7.921	
95% CI of difference	-38.82 to 11.60	
Row Factor		<0.0001 (****)
Row Factor x Column Factor		<0.0001 (****)
Column Factor		0.1843 (ns)

C

LOR RvsERL+LOR R-H2228 LOR R (Two-way ANOVA)

Difference between column means	Value	P value
Mean of LOR R	109.0	
Mean of ERL+LOR R	135.0	
Difference between means	26.02	
SE of difference	12.13	
95% CI of difference	-26.17 to 78.21	
Row Factor		<0.0001 (****)
Row Factor x Column Factor		0.0091 (****)
Column Factor		0.1651 (ns)

D

LOR RvsSAP+LOR R-H2228 LOR R (Two-way ANOVA)

Difference between column means	Value	P value
Mean of LOR R	109.0	
Mean of SAP+LOR R	98.46	
Difference between means	-11.11	
SE of difference	1.210	
95% CI of difference	-14.96 to -7.259	
Row Factor		<0.0001 (****)
Row Factor x Column Factor		<0.0001 (****)
Column Factor		0.0027 (**)

E

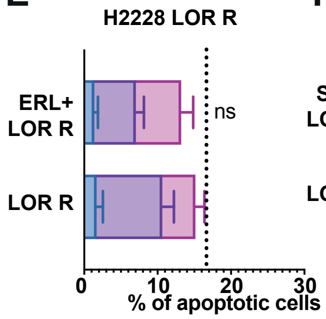

F

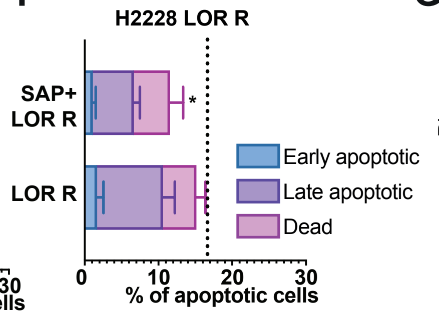

G

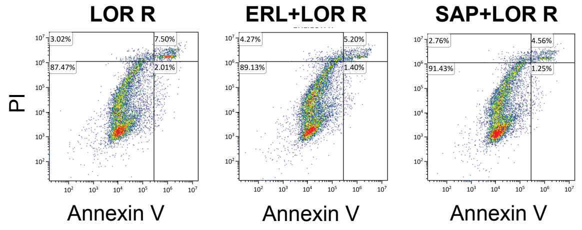

H

LOR R+HRGvsERL+LOR R+HRG-H2228 LOR R (Two-way ANOVA)

Difference between column means	Value	P value
Mean of LOR R+HRG	146.6	
Mean of ERL+LOR R+HRG	71.61	
Difference between means	-75.00	
SE of difference	11.50	
95% CI of difference	-111.6 to -38.41	
Row Factor		<0.0001 (****)
Row Factor x Column Factor		<0.0001 (****)
Column Factor		0.0073 (***)

I

LOR R+HRGvsSAP+LOR R+HRG-H2228 LOR R (Two-way ANOVA)

Difference between column means	Value	P value
Mean of LOR R+HRG	146.6	
Mean of SAP+LOR R+HRG	46.30	
Difference between means	-100.3	
SE of difference	6.126	
95% CI of difference	-119.8 to -80.81	
Row Factor		0.0006 (***)
Row Factor x Column Factor		<0.0001 (****)
Column Factor		0.0005 (***)

J

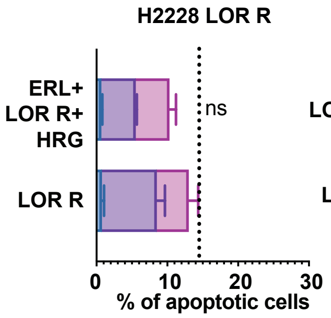

K

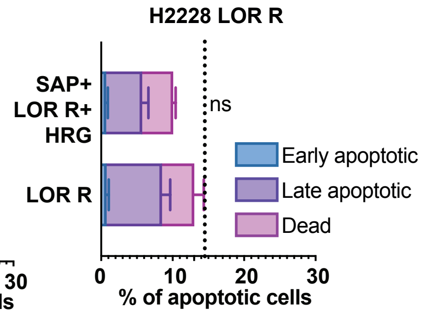

L

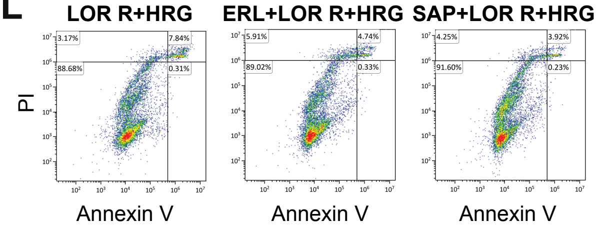

M

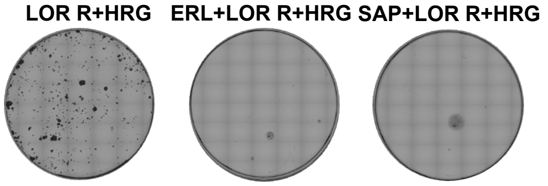

A

LOR RvsAKT VIII+LOR R-H2228 (Two-way ANOVA)

Difference between column means	Value	P value
Mean of LOR R	109.0	
Mean of AKT VIII+LOR R	76.58	
Difference between means	-33.36	
SE of difference	8.893	
95% CI of difference	-61.66 to -5.057	
Row Factor		<0.0001 (****)
Row Factor x Column Factor		<0.0001 (****)
Column Factor		0.0331 (*)

B

LOR R+HRGvsAKT VIII+LOR R+HRG-H2228 (Two-way ANOVA)

Difference between column means	Value	P value
Mean of LOR R+HRG	146.6	
Mean of AKT VIII+LOR R+HRG	55.58	
Difference between means	-91.03	
SE of difference	8.352	
95% CI of difference	-117.6 to -64.45	
Row Factor		0.0004 (***)
Row Factor x Column Factor		0.0004 (***)
Column Factor		0.0017 (**)

C

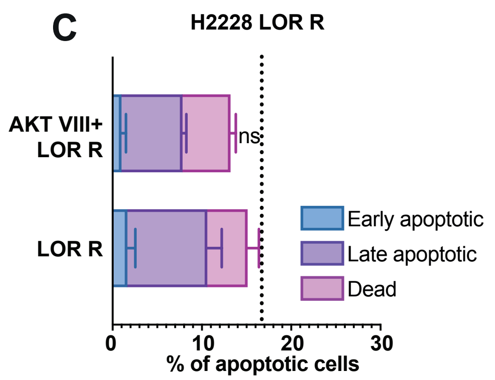

D

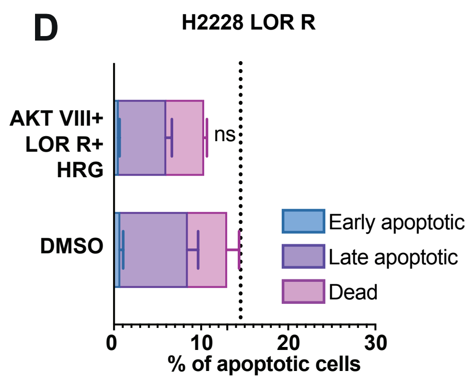

E

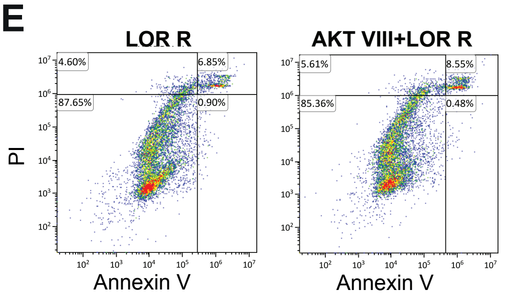

F

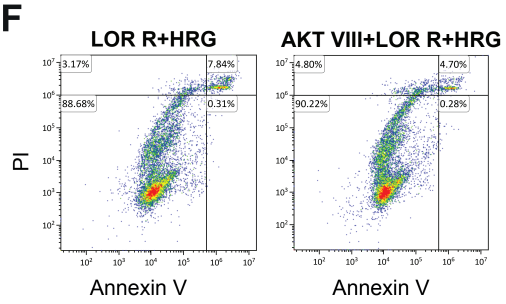

Supplement: Supplementary file 2 — Supplementary Figs. [file 41419_2024_7272_MOESM2_ESM.pdf]
