# Supplementary material for: Targeting ERBB3 and AKT to overcome adaptive resistance in EML4-ALK-driven non-small cell lung cancer
Source: Cell Death Dis. 2024 Dec 18;15(12):912. doi: 10.1038/s41419-024-07272-7 (PMC11655848; doi:10.1038/s41419-024-07272-7)
Supplement: Supplementary file 3 — Supplemental Material Raw Blots [file 41419_2024_7272_MOESM3_ESM.pdf]

Figure 4

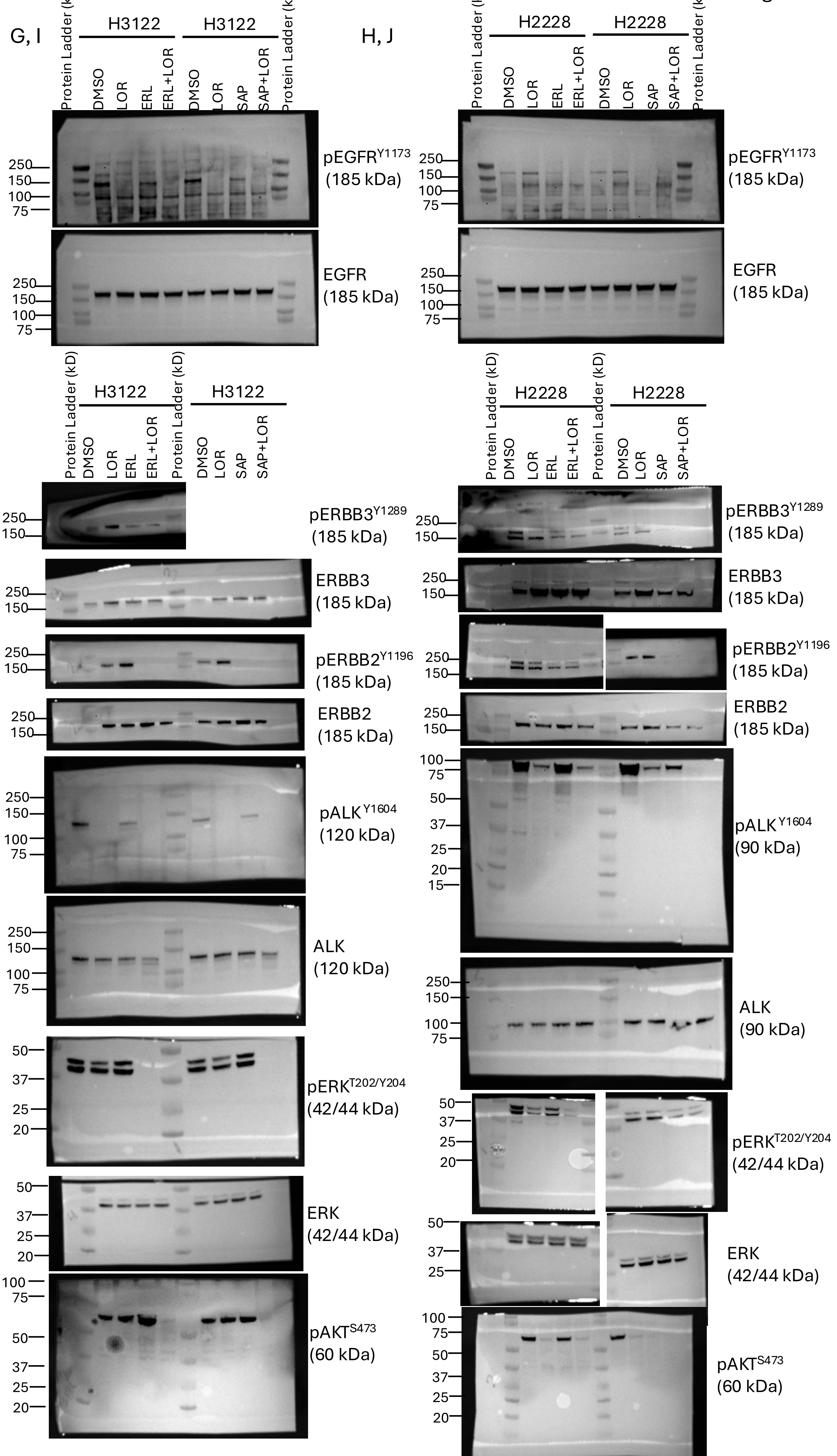

G, I
H, J
H3122
H2228
Protein Ladder (kD)
DMSO
LOR
ERL
ERL+LOR
SAP
SAP+LOR
pEGFR^{Y1173} (185 kDa)
EGFR (185 kDa)
pERBB3^{Y1289} (185 kDa)
ERBB3 (185 kDa)
pERBB2^{Y1196} (185 kDa)
ERBB2 (185 kDa)
pALK^{Y1604} (120 kDa)
ALK (120 kDa)
pALK^{Y1604} (90 kDa)
ALK (90 kDa)
pERK$^{T202/Y204}$ (42/44 kDa)
ERK (42/44 kDa)
pAKT^{S473} (60 kDa)
250
150
100
75
50
37
25
20
15

Figure 4

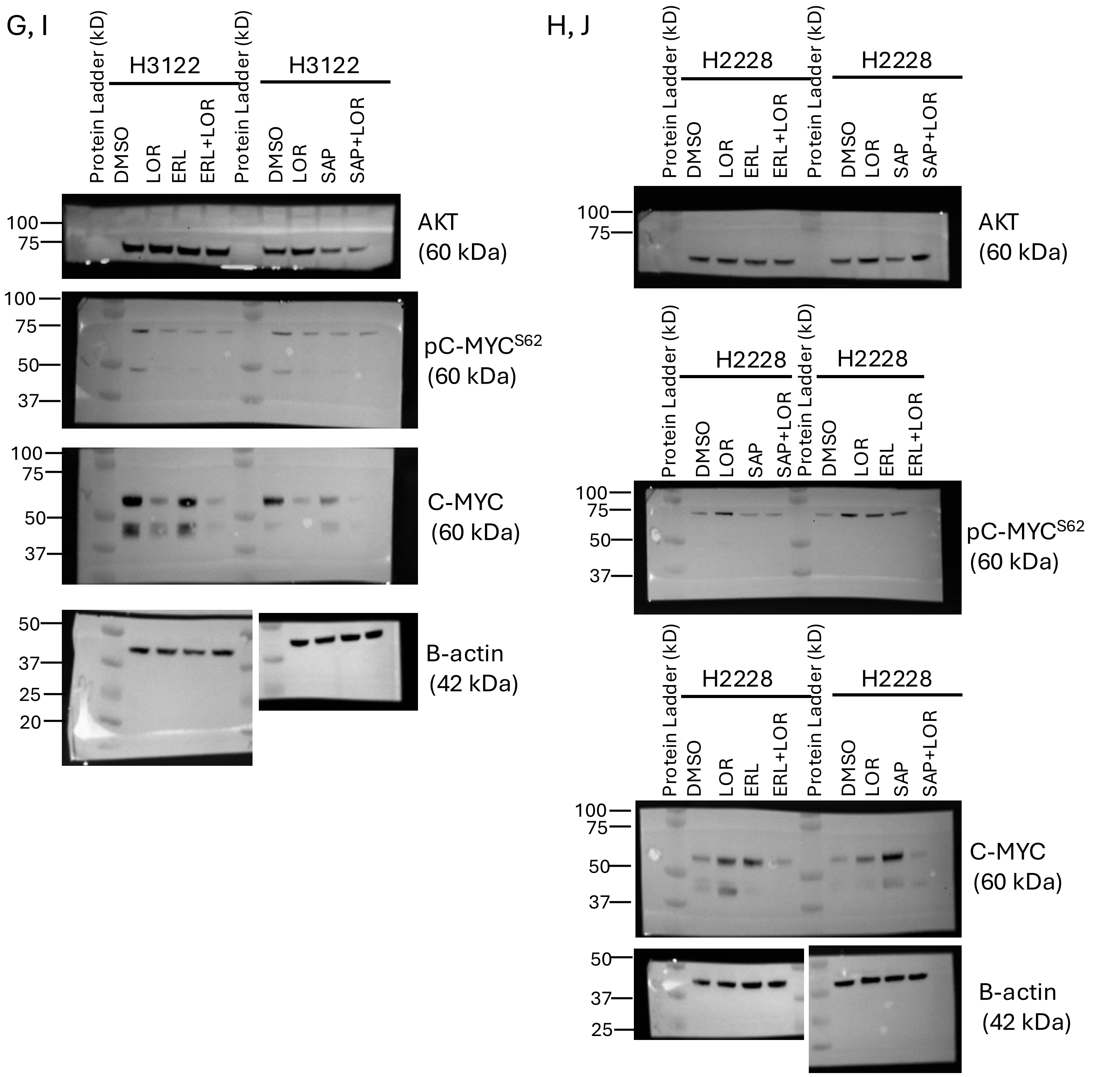
G, I
Protein Ladder (kD)
H3122
DMSO
LOR
ERL
ERL+LOR
Protein Ladder (kD)
H3122
DMSO
LOR
SAP
SAP+LOR
100
75
AKT
(60 kDa)
100
75
50
37
pC-MYC^{S62}
(60 kDa)
100
75
50
37
C-MYC
(60 kDa)
50
37
25
20
B-actin
(42 kDa)
H, J
Protein Ladder (kD)
H2228
DMSO
LOR
ERL
ERL+LOR
Protein Ladder (kD)
H2228
DMSO
LOR
SAP
SAP+LOR
100
75
AKT
(60 kDa)
Protein Ladder (kD)
H2228
DMSO
LOR
SAP
SAP+LOR
Protein Ladder (kD)
H2228
DMSO
LOR
ERL
ERL+LOR
100
75
50
37
pC-MYC^{S62}
(60 kDa)
Protein Ladder (kD)
H2228
DMSO
LOR
ERL
ERL+LOR
Protein Ladder (kD)
H2228
DMSO
LOR
SAP
SAP+LOR
100
75
50
37
C-MYC
(60 kDa)
50
37
25
B-actin
(42 kDa)

Figure 5

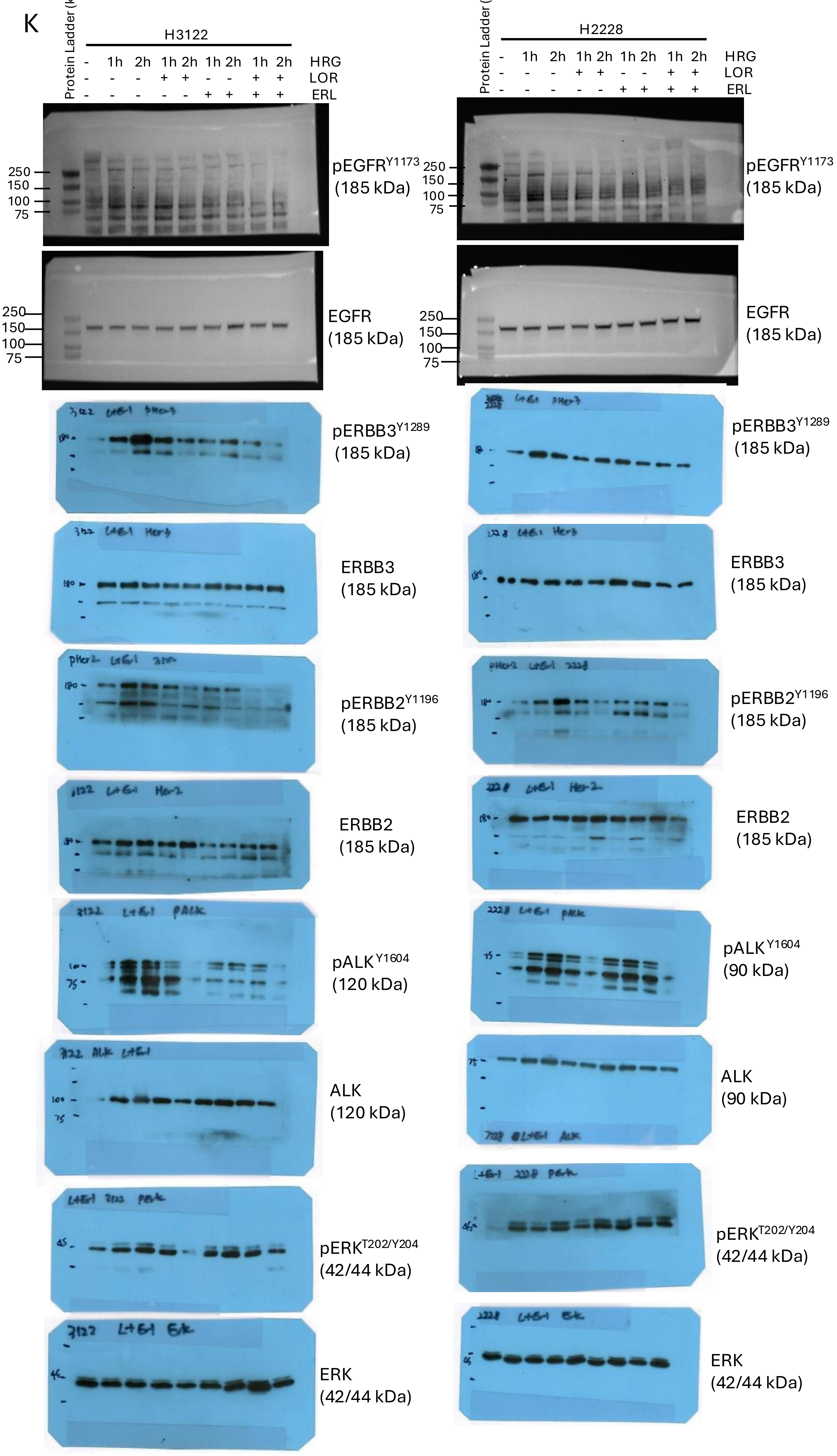
K
Protein Ladder (kD)
H3122
- 1h 2h 1h 2h 1h 2h 1h 2h HRG
- - - + + - - + + LOR
- - - - - + + + + ERL
250
150
100
75
pEGFR Y1173 (185 kDa)
EGFR (185 kDa)
pERBB3 Y1289 (185 kDa)
ERBB3 (185 kDa)
pERBB2 Y1196 (185 kDa)
ERBB2 (185 kDa)
pALK Y1604 (120 kDa)
ALK (120 kDa)
pERK T202/Y204 (42/44 kDa)
ERK (42/44 kDa)
H2228
pALK Y1604 (90 kDa)
ALK (90 kDa)

Figure 5

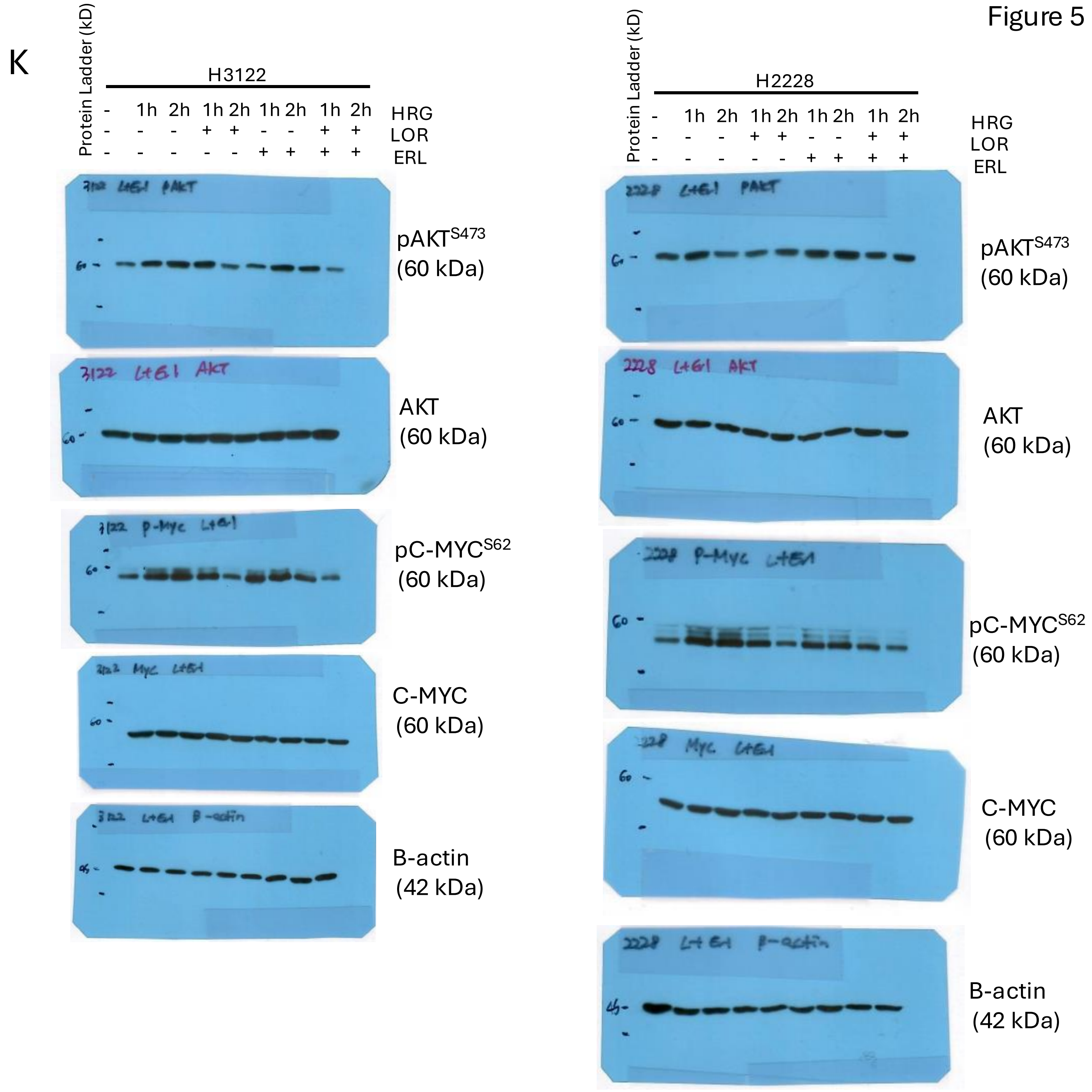
K
Protein Ladder (kD)
H3122
- 1h 2h 1h 2h 1h 2h 1h 2h HRG
- - - + + - - + + LOR
- - - - - + + + + ERL
pAKT^{S473} (60 kDa)
AKT (60 kDa)
pC-MYC^{S62} (60 kDa)
C-MYC (60 kDa)
B-actin (42 kDa)
Protein Ladder (kD)
H2228
- 1h 2h 1h 2h 1h 2h 1h 2h HRG
- - - + + - - + + LOR
- - - - - + + + + ERL
pAKT^{S473} (60 kDa)
AKT (60 kDa)
pC-MYC^{S62} (60 kDa)
C-MYC (60 kDa)
B-actin (42 kDa)

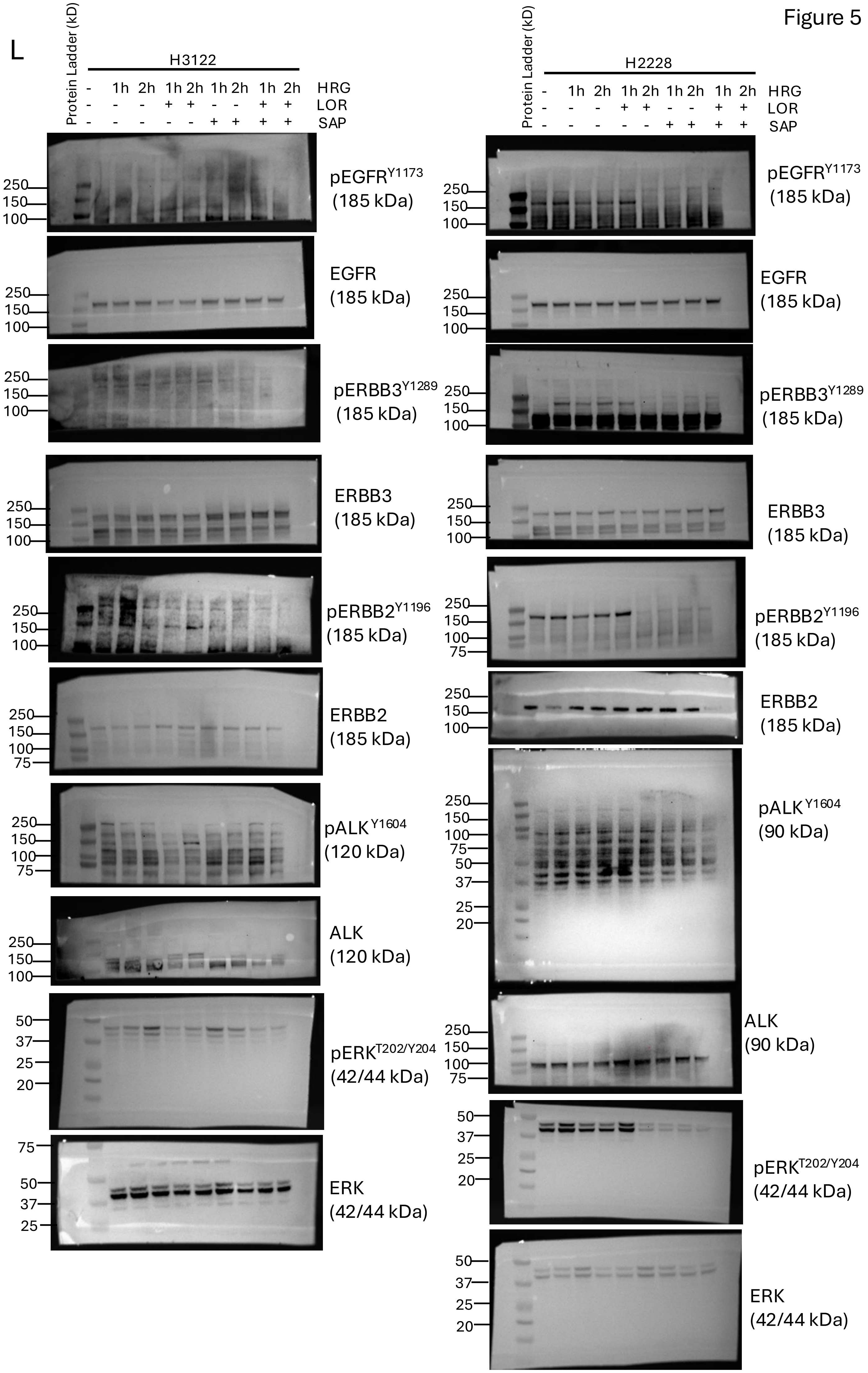
Figure 5
L
Protein Ladder (kD)
H3122
H2228
- 1h 2h 1h 2h 1h 2h 1h 2h HRG
- - - + + - - + + LOR
- - - - - + + + + SAP
pEGFR Y1173 (185 kDa)
EGFR (185 kDa)
pERBB3 Y1289 (185 kDa)
ERBB3 (185 kDa)
pERBB2 Y1196 (185 kDa)
ERBB2 (185 kDa)
pALK Y1604 (120 kDa)
ALK (120 kDa)
pALK Y1604 (90 kDa)
ALK (90 kDa)
pERK T202/Y204 (42/44 kDa)
ERK (42/44 kDa)
250
150
100
75
50
37
25
20

Figure 5

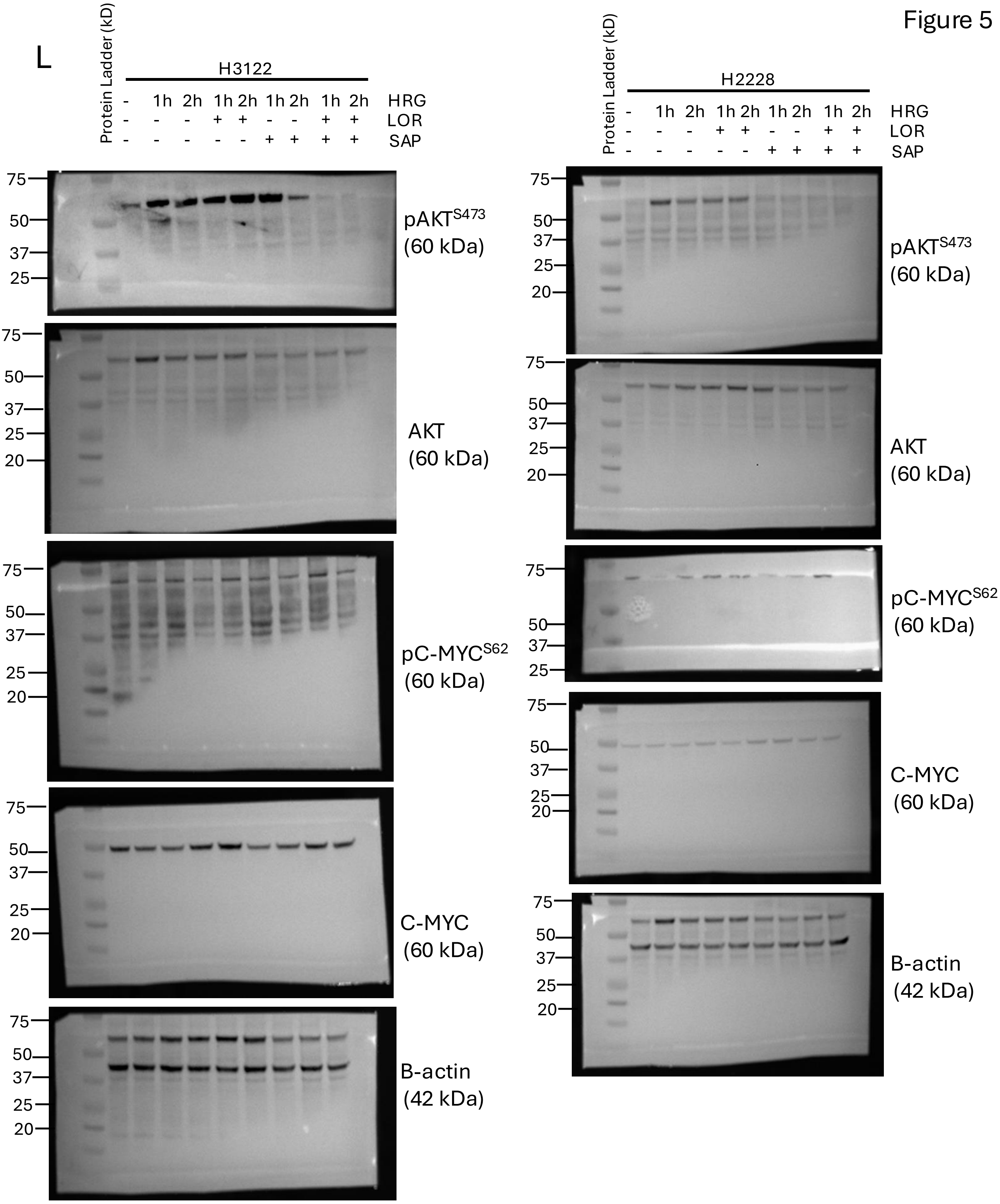
L
Protein Ladder (kD)
H3122
- 1h 2h 1h 2h 1h 2h 1h 2h HRG
- - - + + - - + + LOR
- - - - - + + + + SAP
75
50
37
25
pAKT^S473
(60 kDa)
75
50
37
25
20
AKT
(60 kDa)
75
50
37
25
20
pC-MYC^S62
(60 kDa)
75
50
37
25
20
C-MYC
(60 kDa)
75
50
37
25
20
B-actin
(42 kDa)
Protein Ladder (kD)
H2228
- 1h 2h 1h 2h 1h 2h 1h 2h HRG
- - - + + - - + + LOR
- - - - - + + + + SAP
75
50
37
25
20
pAKT^S473
(60 kDa)
75
50
37
25
20
AKT
(60 kDa)
75
50
37
25
pC-MYC^S62
(60 kDa)
75
50
37
25
20
C-MYC
(60 kDa)
75
50
37
25
20
B-actin
(42 kDa)

Figure 6

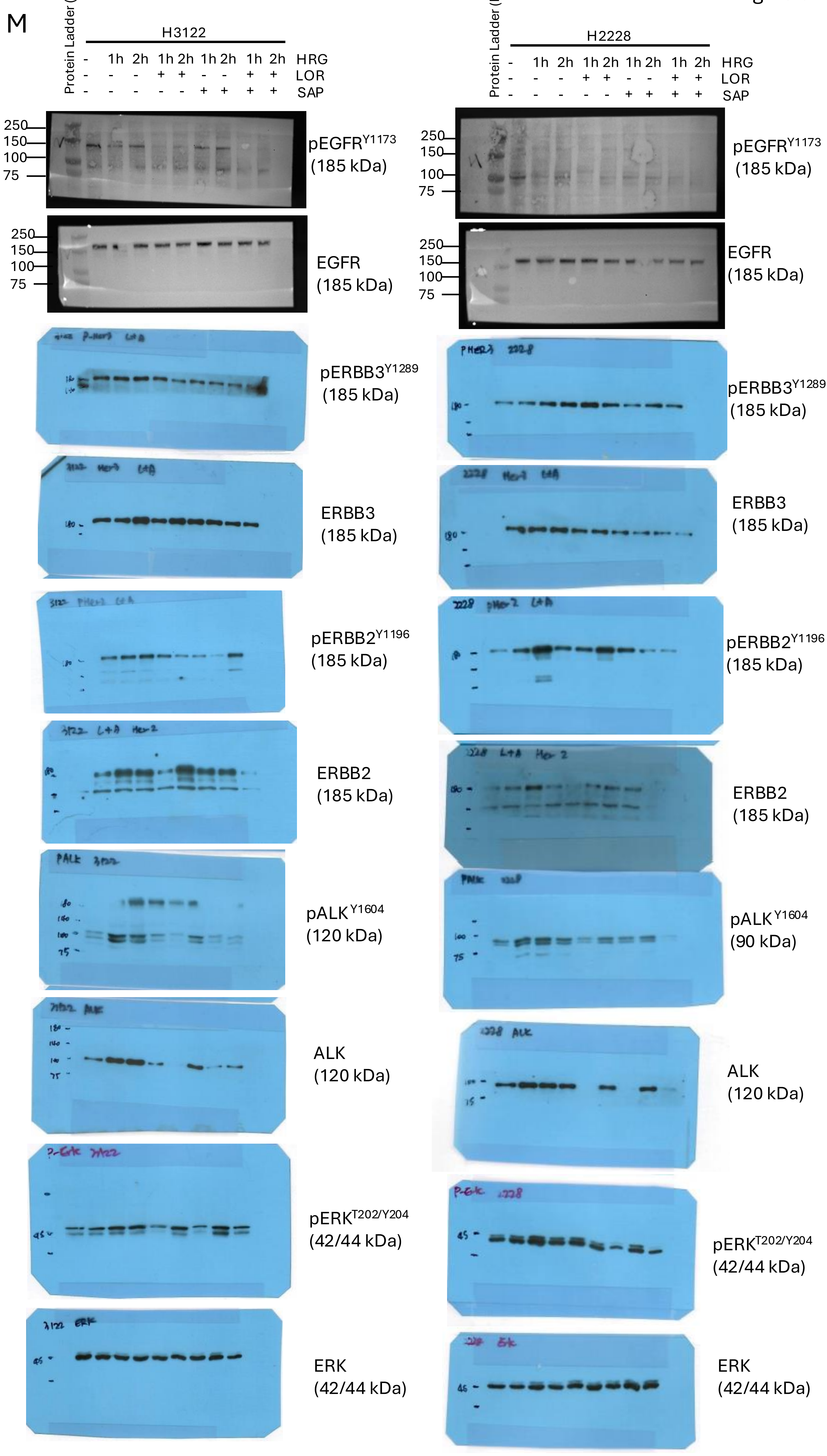
M
Protein Ladder (kD)
H3122
- 1h 2h 1h 2h 1h 2h 1h 2h HRG
- - - + + - - + + LOR
- - - - - + + + + SAP
250
150
100
75
pEGFR^{Y1173} (185 kDa)
EGFR (185 kDa)
pERBB3^{Y1289} (185 kDa)
ERBB3 (185 kDa)
pERBB2^{Y1196} (185 kDa)
ERBB2 (185 kDa)
pALK^{Y1604} (120 kDa)
ALK (120 kDa)
pERK$^{T202/Y204}$ (42/44 kDa)
ERK (42/44 kDa)
Protein Ladder (kD)
H2228
- 1h 2h 1h 2h 1h 2h 1h 2h HRG
- - - + + - - + + LOR
- - - - - + + + + SAP
250
150
100
75
pEGFR^{Y1173} (185 kDa)
EGFR (185 kDa)
pERBB3^{Y1289} (185 kDa)
ERBB3 (185 kDa)
pERBB2^{Y1196} (185 kDa)
ERBB2 (185 kDa)
pALK^{Y1604} (90 kDa)
ALK (120 kDa)
pERK$^{T202/Y204}$ (42/44 kDa)
ERK (42/44 kDa)

Figure 6

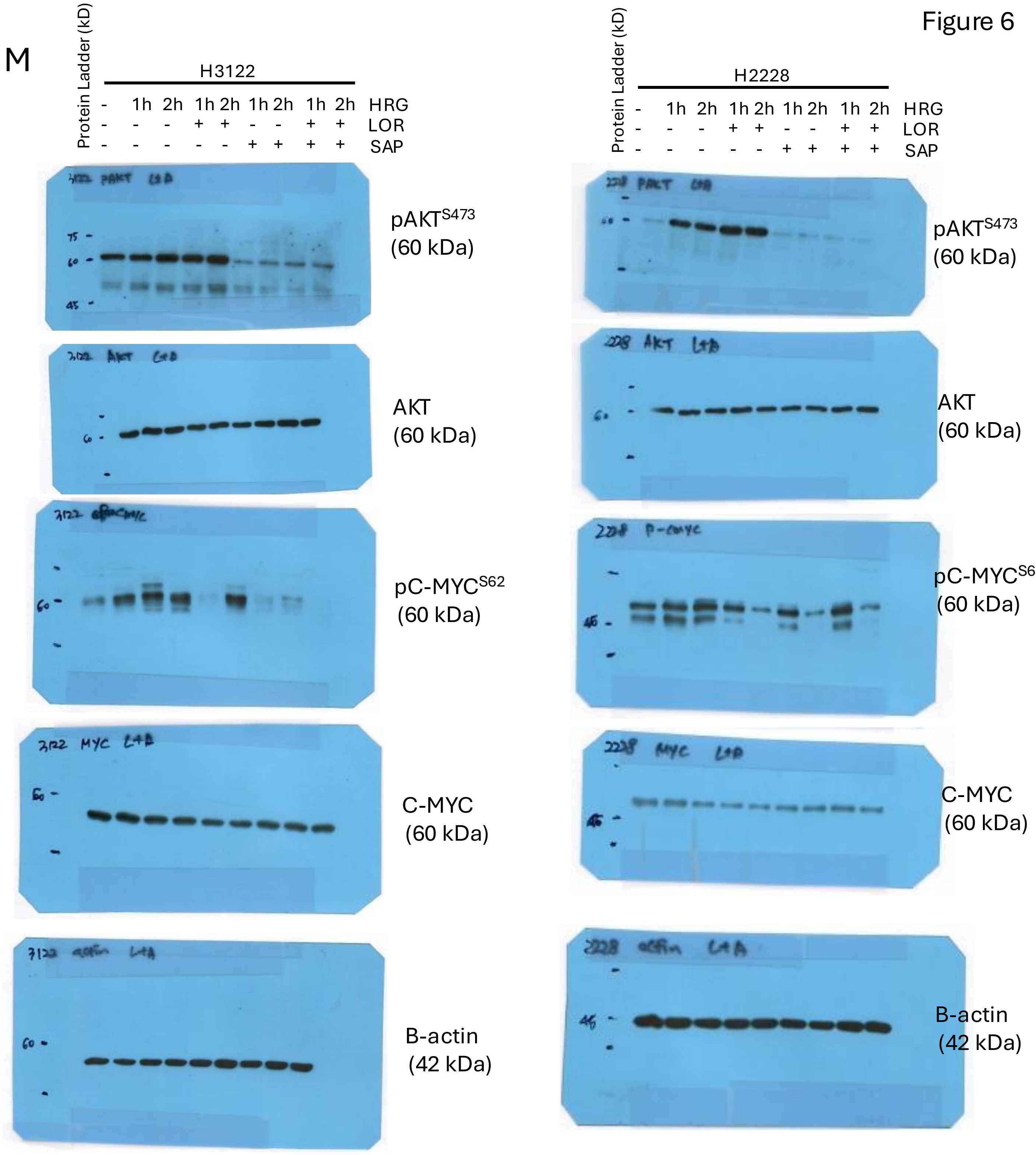
M
Protein Ladder (kD)
H3122
- 1h 2h 1h 2h 1h 2h 1h 2h HRG
- - - + + - - + + LOR
- - - - - + + + + SAP
pAKTS473 (60 kDa)
AKT (60 kDa)
pC-MYCS62 (60 kDa)
C-MYC (60 kDa)
B-actin (42 kDa)
Protein Ladder (kD)
H2228
- 1h 2h 1h 2h 1h 2h 1h 2h HRG
- - - + + - - + + LOR
- - - - - + + + + SAP
pAKTS473 (60 kDa)
AKT (60 kDa)
pC-MYCS6 (60 kDa)
C-MYC (60 kDa)
B-actin (42 kDa)

Figure 7

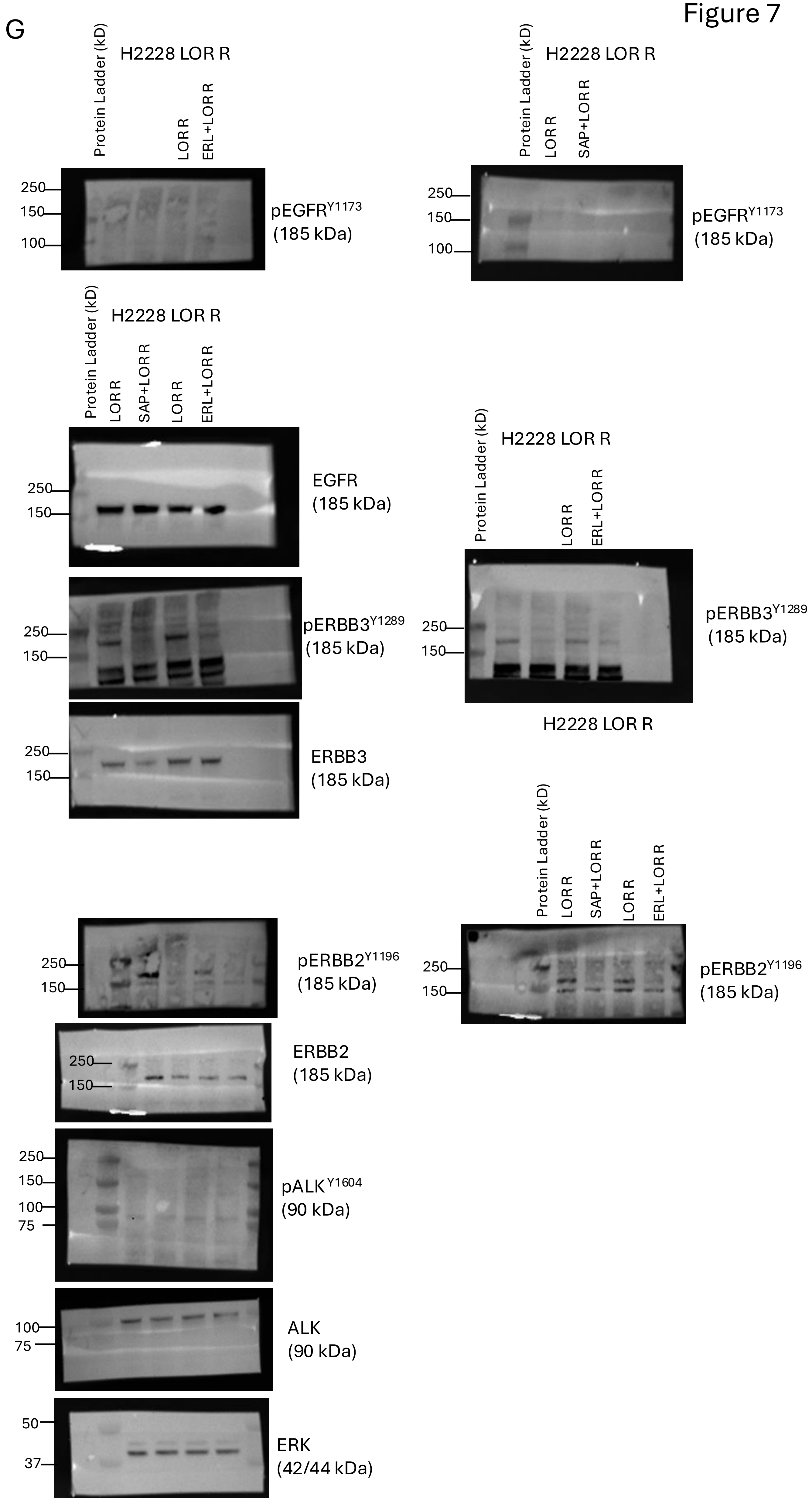

G
Protein Ladder (kD)
H2228 LOR R
LOR R
ERL+LOR R
250
150
100
pEGFR Y1173
(185 kDa)
Protein Ladder (kD)
H2228 LOR R
LOR R
SAP+LOR R
250
150
100
pEGFR Y1173
(185 kDa)
Protein Ladder (kD)
H2228 LOR R
LOR R
SAP+LOR R
LOR R
ERL+LOR R
250
150
EGFR
(185 kDa)
250
150
pERBB3 Y1289
(185 kDa)
250
150
ERBB3
(185 kDa)
Protein Ladder (kD)
H2228 LOR R
LOR R
ERL+LOR R
250
150
pERBB3 Y1289
(185 kDa)
H2228 LOR R
Protein Ladder (kD)
LOR R
SAP+LOR R
LOR R
ERL+LOR R
250
150
pERBB2 Y1196
(185 kDa)
250
150
pERBB2 Y1196
(185 kDa)
250
150
ERBB2
(185 kDa)
250
150
100
75
pALK Y1604
(90 kDa)
100
75
ALK
(90 kDa)
50
37
ERK
(42/44 kDa)

Figure 7

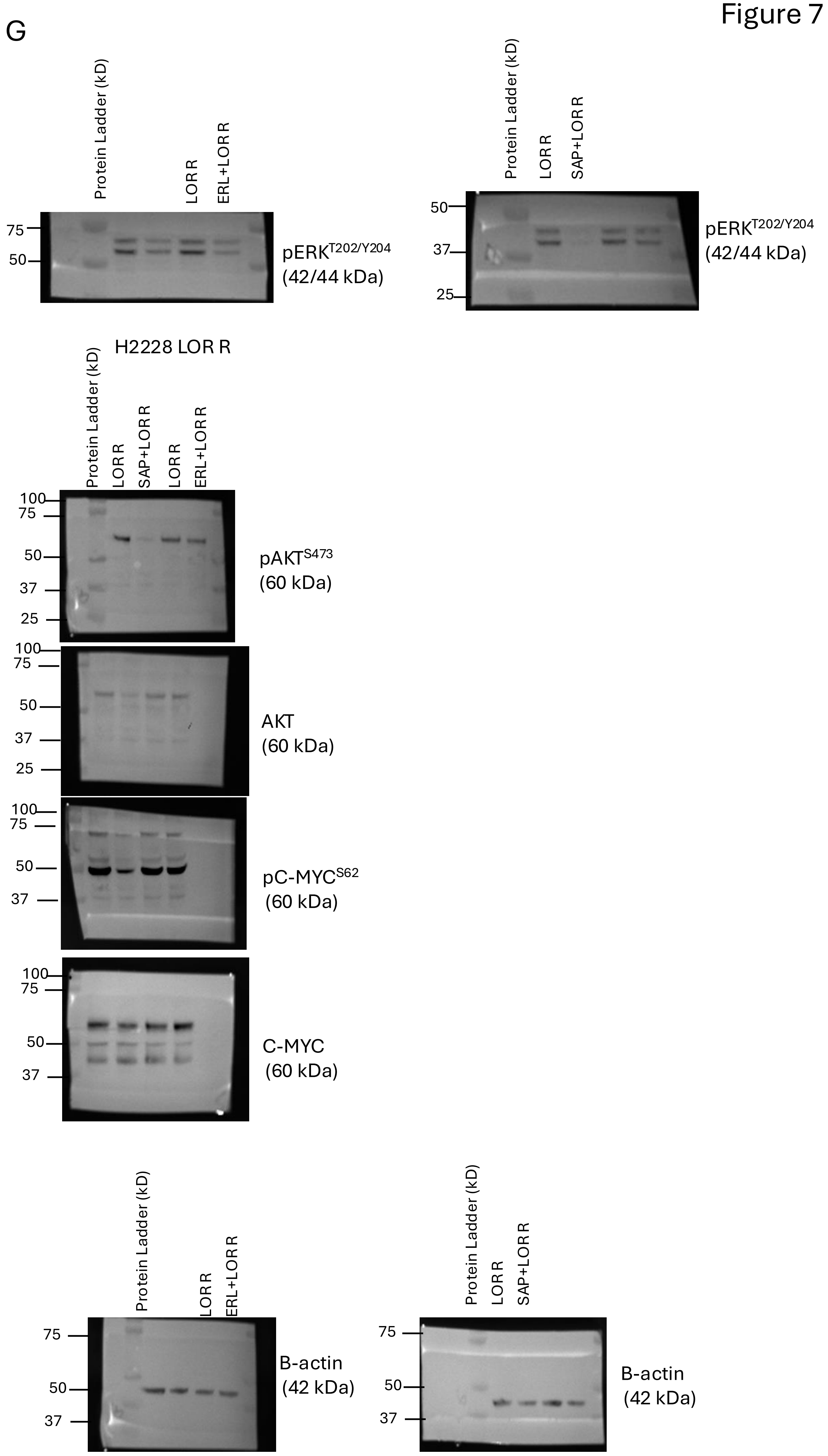
G
Protein Ladder (kD)
LOR R
ERL+LOR R
75
50
pERK T202/Y204
(42/44 kDa)
Protein Ladder (kD)
LOR R
SAP+LOR R
50
37
25
pERK T202/Y204
(42/44 kDa)
H2228 LOR R
Protein Ladder (kD)
LOR R
SAP+LOR R
LOR R
ERL+LOR R
100
75
50
37
25
pAKT S473
(60 kDa)
AKT
(60 kDa)
pC-MYC S62
(60 kDa)
C-MYC
(60 kDa)
Protein Ladder (kD)
LOR R
ERL+LOR R
75
50
37
B-actin
(42 kDa)
Protein Ladder (kD)
LOR R
SAP+LOR R
B-actin
(42 kDa)

Figure 7

L

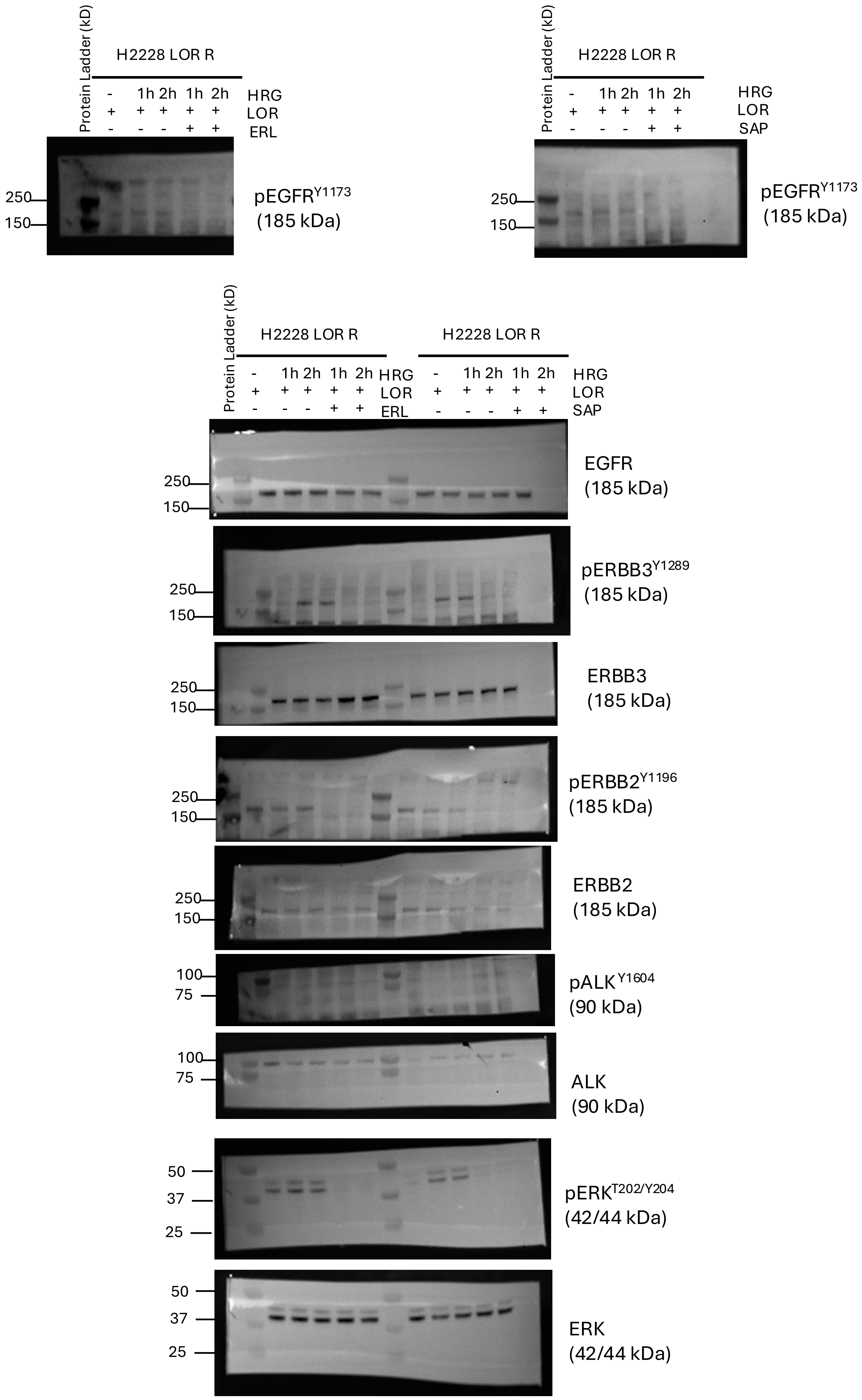

Protein Ladder (kD)
H2228 LOR R
- 1h 2h 1h 2h HRG
+ + + + + LOR
- - - + + ERL
250
150
pEGFR^Y1173
(185 kDa)
Protein Ladder (kD)
H2228 LOR R
- 1h 2h 1h 2h HRG
+ + + + + LOR
- - - + + SAP
250
150
pEGFR^Y1173
(185 kDa)
Protein Ladder (kD)
H2228 LOR R
H2228 LOR R
- 1h 2h 1h 2h HRG - 1h 2h 1h 2h HRG
+ + + + + LOR + + + + + LOR
- - - + + ERL - - - + + SAP
250
150
EGFR
(185 kDa)
250
150
pERBB3^Y1289
(185 kDa)
250
150
ERBB3
(185 kDa)
250
150
pERBB2^Y1196
(185 kDa)
250
150
ERBB2
(185 kDa)
100
75
pALK^Y1604
(90 kDa)
100
75
ALK
(90 kDa)
50
37
25
pERK^T202/Y204
(42/44 kDa)
50
37
25
ERK
(42/44 kDa)

Figure 7

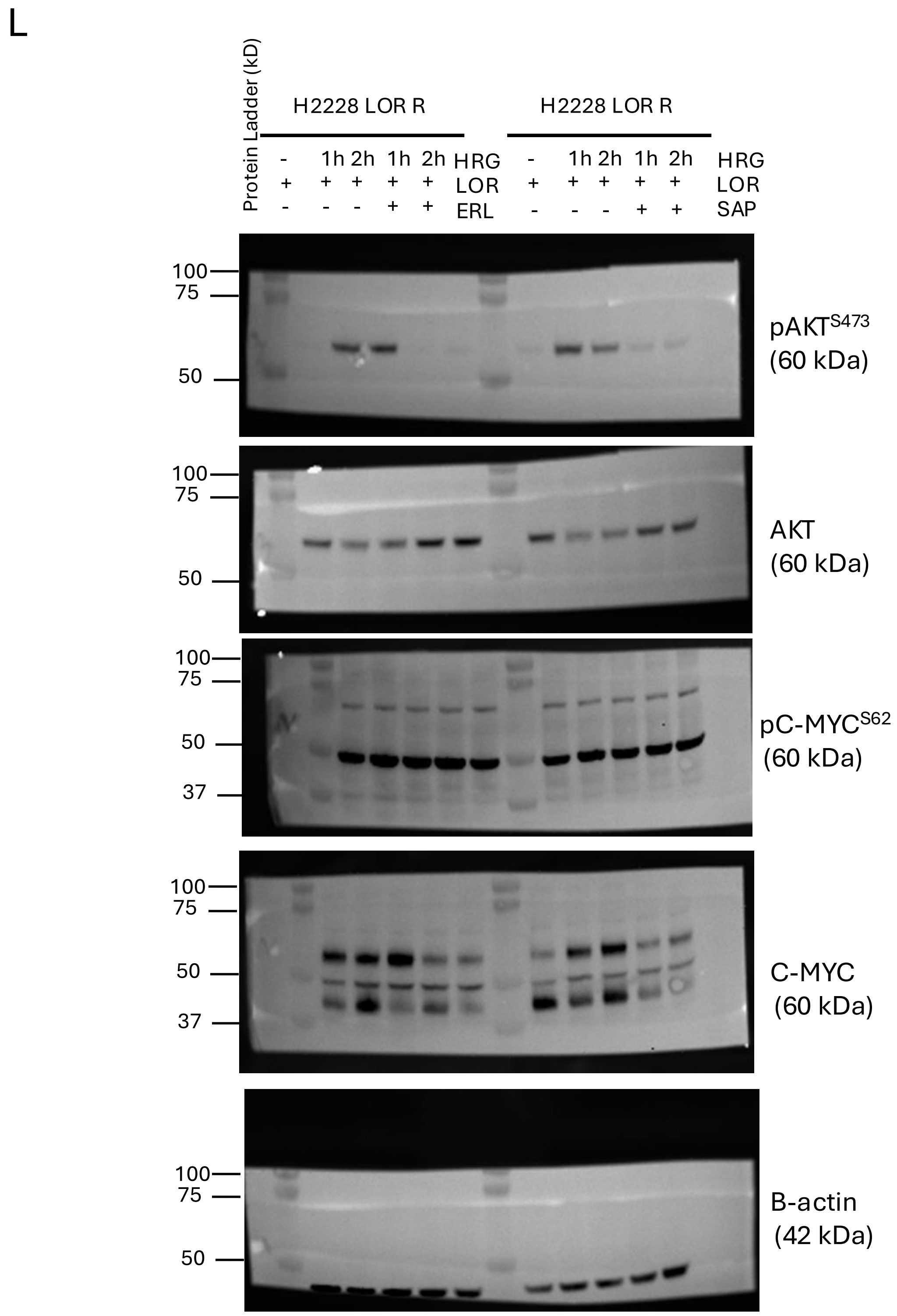
L
Protein Ladder (kD)
H2228 LOR R
H2228 LOR R
- 1h 2h 1h 2h HRG
+ + + + + LOR
- - - + + ERL
- 1h 2h 1h 2h HRG
+ + + + + LOR
- - - + + SAP
100
75
50
37
pAKT^S473 (60 kDa)
AKT (60 kDa)
pC-MYC^S62 (60 kDa)
C-MYC (60 kDa)
B-actin (42 kDa)

Figure 8

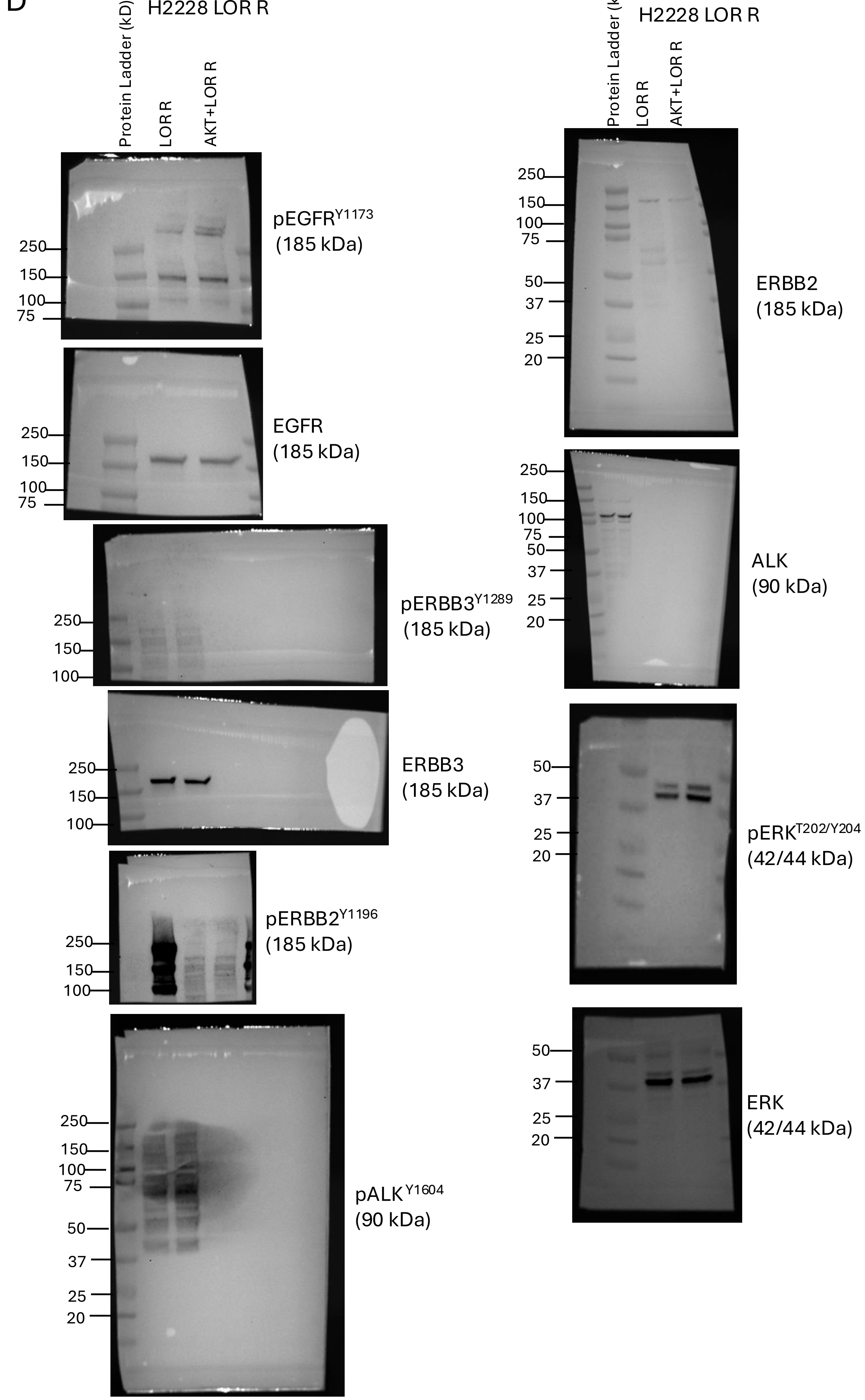
D
H2228 LOR R
Protein Ladder (kD)
LOR R
AKT+LOR R
pEGFR Y1173
(185 kDa)
EGFR
(185 kDa)
pERBB3 Y1289
(185 kDa)
ERBB3
(185 kDa)
pERBB2 Y1196
(185 kDa)
pALK Y1604
(90 kDa)
ERBB2
(185 kDa)
ALK
(90 kDa)
pERK T202/Y204
(42/44 kDa)
ERK
(42/44 kDa)
250
150
100
75
50
37
25
20

Figure 8

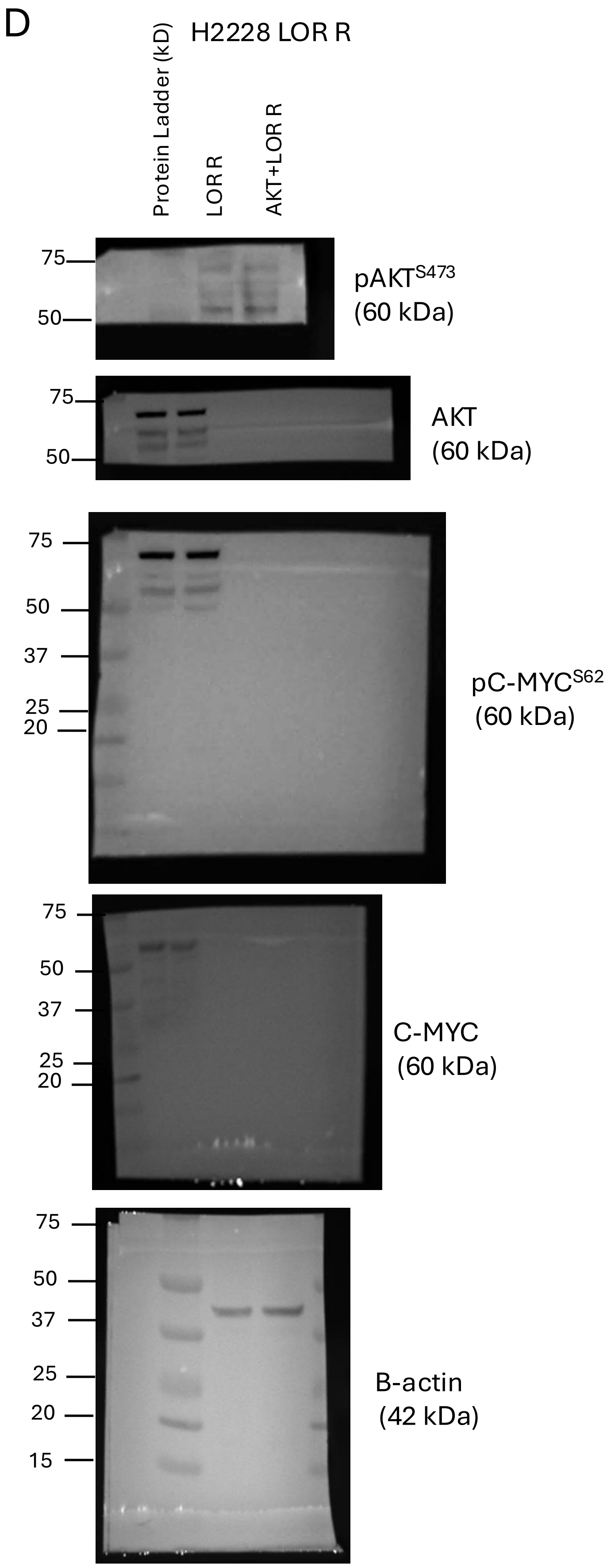
D
H2228 LOR R
Protein Ladder (kD)
LOR R
AKT+LOR R
75
50
pAKT^{S473}
(60 kDa)
75
50
AKT
(60 kDa)
75
50
37
25
20
pC-MYC^{S62}
(60 kDa)
75
50
37
25
20
C-MYC
(60 kDa)
75
50
37
25
20
15
B-actin
(42 kDa)

Figure 8

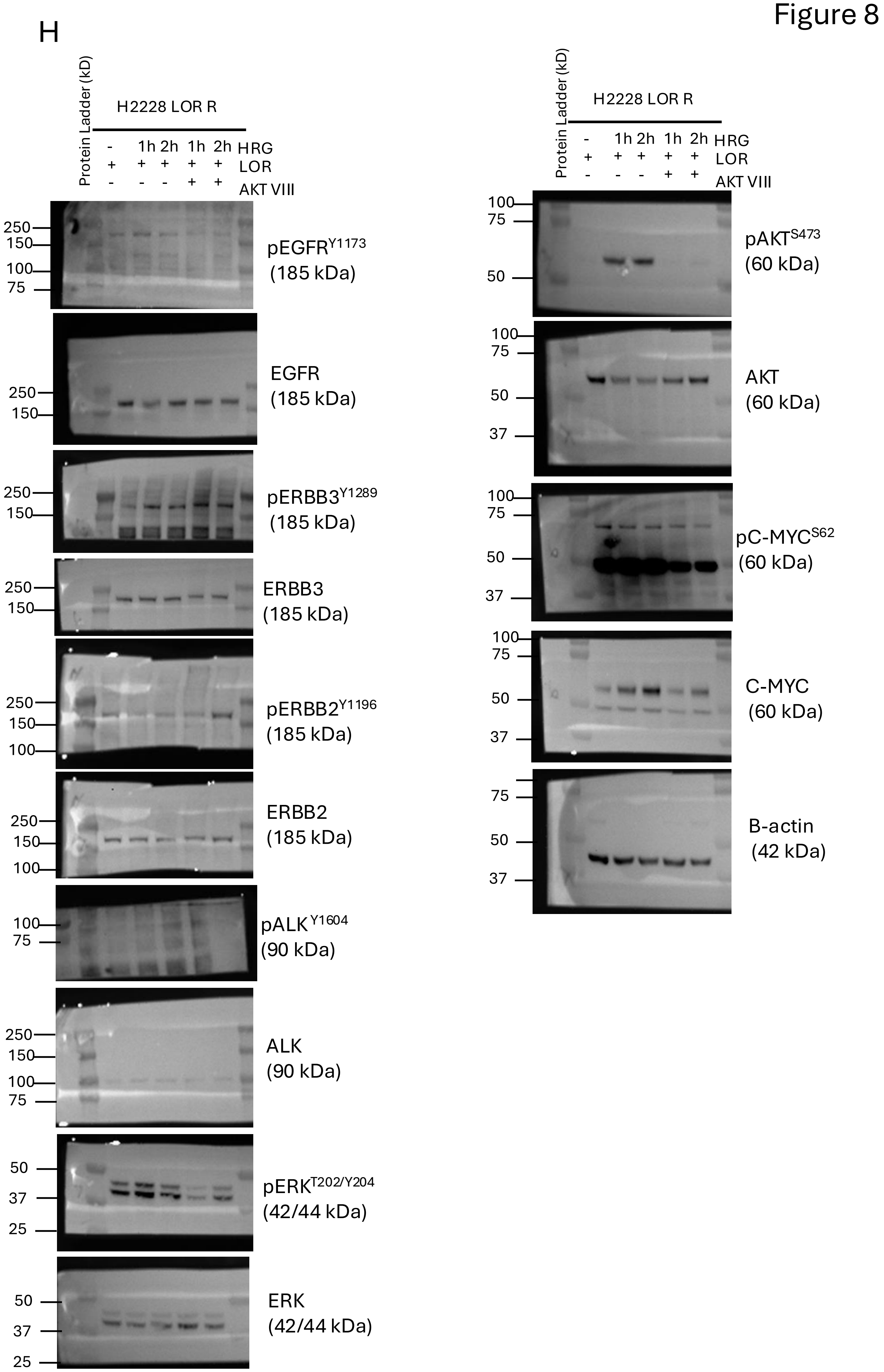
H
Protein Ladder (kD)
H2228 LOR R
- 1h 2h 1h 2h HRG
+ + + + + LOR
- - - + + AKT VIII
250 150 100 75
pEGFRY1173 (185 kDa)
EGFR (185 kDa)
pERBB3Y1289 (185 kDa)
ERBB3 (185 kDa)
pERBB2Y1196 (185 kDa)
ERBB2 (185 kDa)
pALKY1604 (90 kDa)
ALK (90 kDa)
pERKT202/Y204 (42/44 kDa)
ERK (42/44 kDa)
pAKTS473 (60 kDa)
AKT (60 kDa)
pC-MYCS62 (60 kDa)
C-MYC (60 kDa)
B-actin (42 kDa)

Figure S7

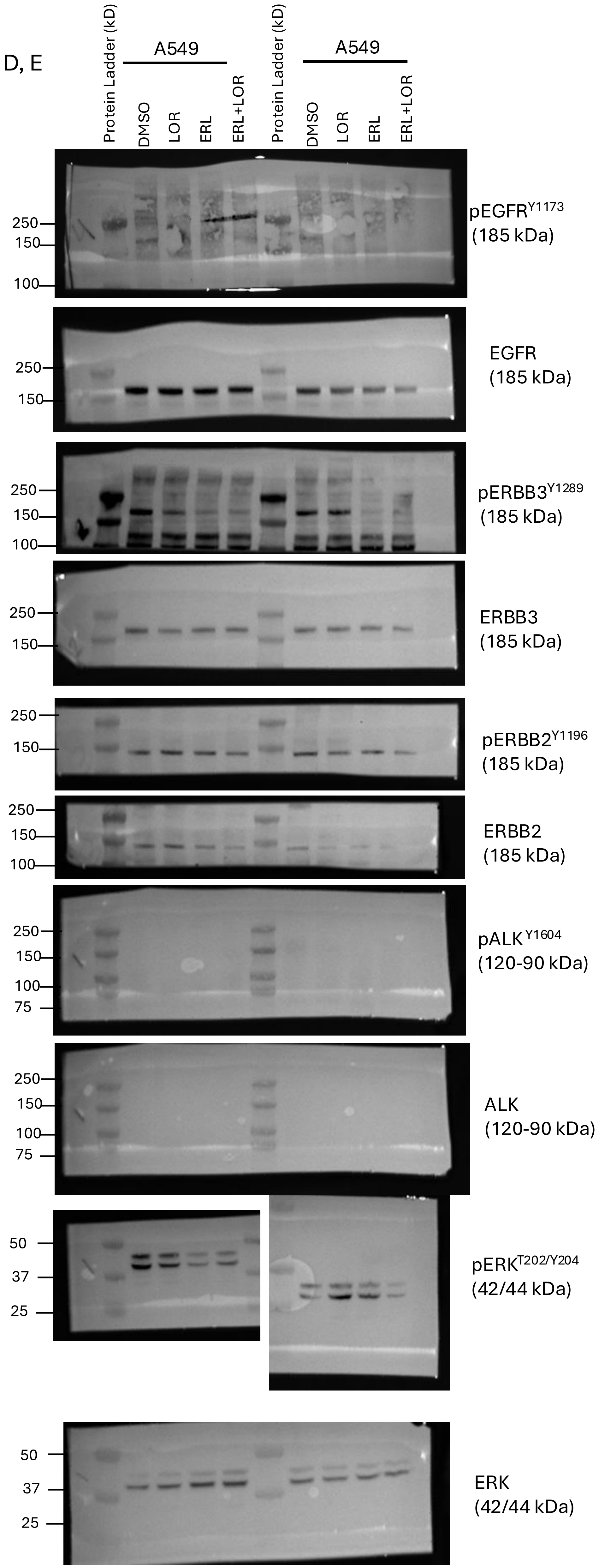
D, E
Protein Ladder (kD)
A549
DMSO
LOR
ERL
ERL+LOR
Protein Ladder (kD)
A549
DMSO
LOR
ERL
ERL+LOR
250
150
100
pEGFR^{Y1173}
(185 kDa)
250
150
EGFR
(185 kDa)
250
150
100
pERBB3^{Y1289}
(185 kDa)
250
150
ERBB3
(185 kDa)
250
150
pERBB2^{Y1196}
(185 kDa)
250
150
100
ERBB2
(185 kDa)
250
150
100
75
pALK^{Y1604}
(120-90 kDa)
250
150
100
75
ALK
(120-90 kDa)
50
37
25
pERK$^{T202/Y204}$
(42/44 kDa)
50
37
25
ERK
(42/44 kDa)

Figure S7

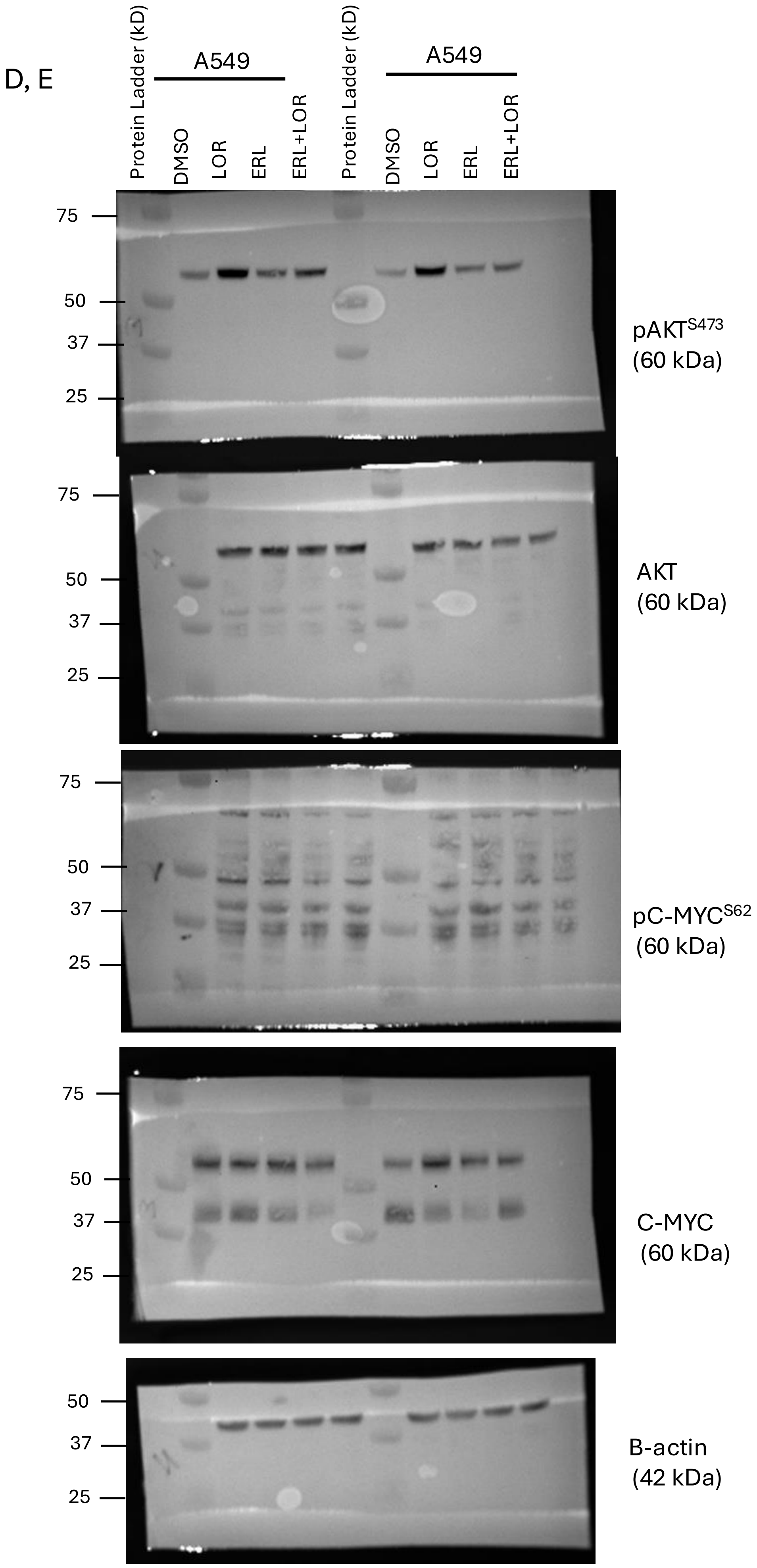

D, E
Protein Ladder (kD)
A549
DMSO
LOR
ERL
ERL+LOR
Protein Ladder (kD)
A549
DMSO
LOR
ERL
ERL+LOR
75
50
37
25
pAKT S473 (60 kDa)
75
50
37
25
AKT (60 kDa)
75
50
37
25
pC-MYC S62 (60 kDa)
75
50
37
25
C-MYC (60 kDa)
50
37
25
B-actin (42 kDa)

Figure S8

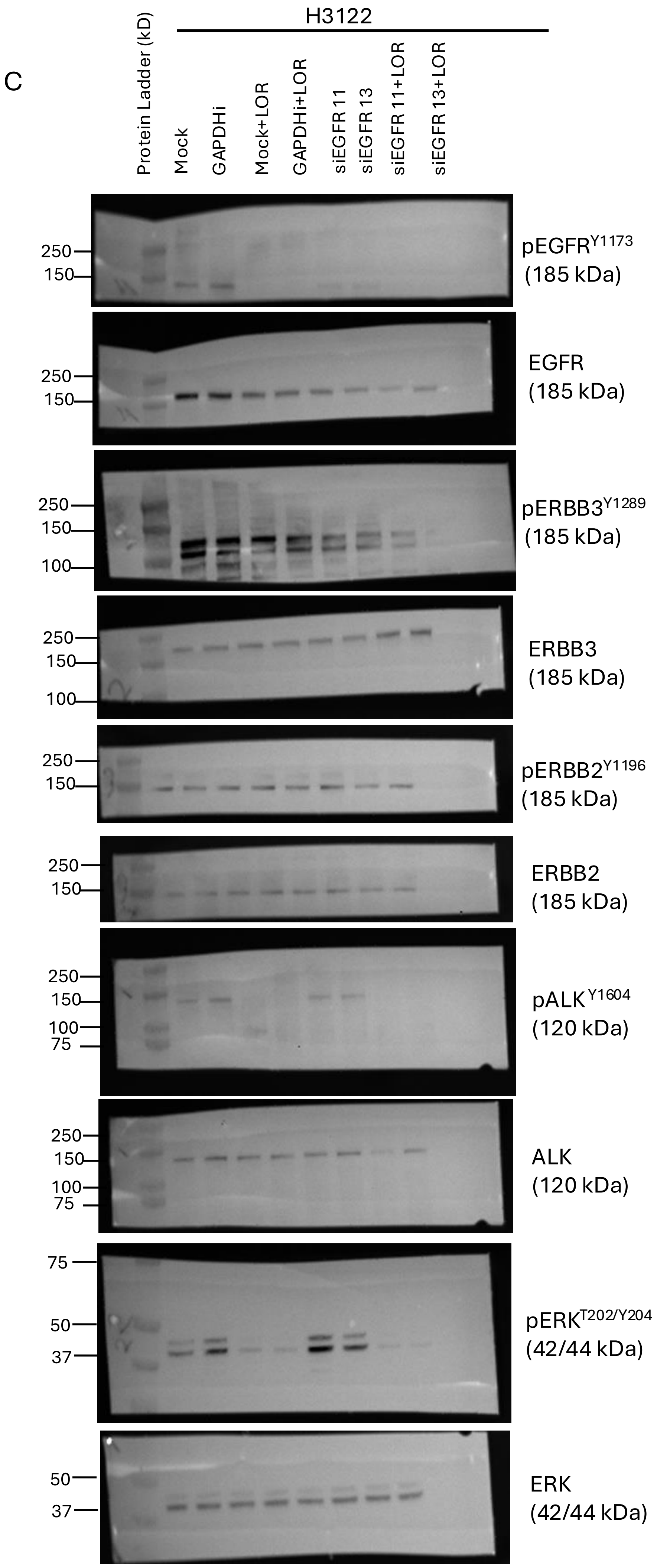
C
H3122
Protein Ladder (kD)
Mock
GAPDHi
Mock+LOR
GAPDHi+LOR
siEGFR11
siEGFR13
siEGFR11+LOR
siEGFR13+LOR
250
150
100
75
50
37
pEGFR Y1173 (185 kDa)
EGFR (185 kDa)
pERBB3 Y1289 (185 kDa)
ERBB3 (185 kDa)
pERBB2 Y1196 (185 kDa)
ERBB2 (185 kDa)
pALK Y1604 (120 kDa)
ALK (120 kDa)
pERK T202/Y204 (42/44 kDa)
ERK (42/44 kDa)

Figure S8

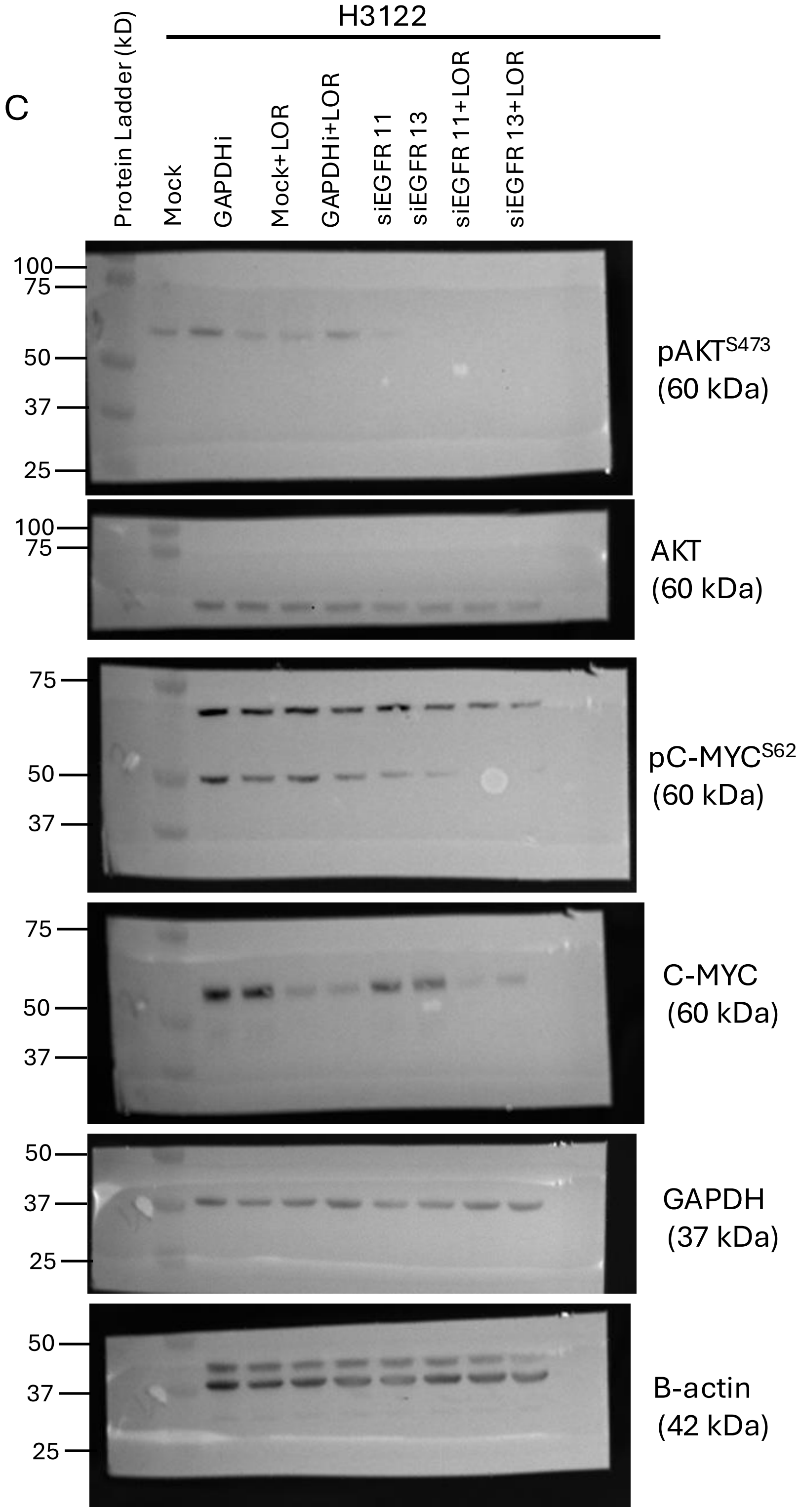

C
H3122
Protein Ladder (kD)
Mock
GAPDHi
Mock+LOR
GAPDHi+LOR
siEGFR 11
siEGFR 13
siEGFR 11+LOR
siEGFR 13+LOR
100
75
50
37
25
pAKT S473
(60 kDa)
100
75
AKT
(60 kDa)
75
50
37
pC-MYC S62
(60 kDa)
75
50
37
C-MYC
(60 kDa)
50
37
25
GAPDH
(37 kDa)
50
37
25
B-actin
(42 kDa)

Figure S8

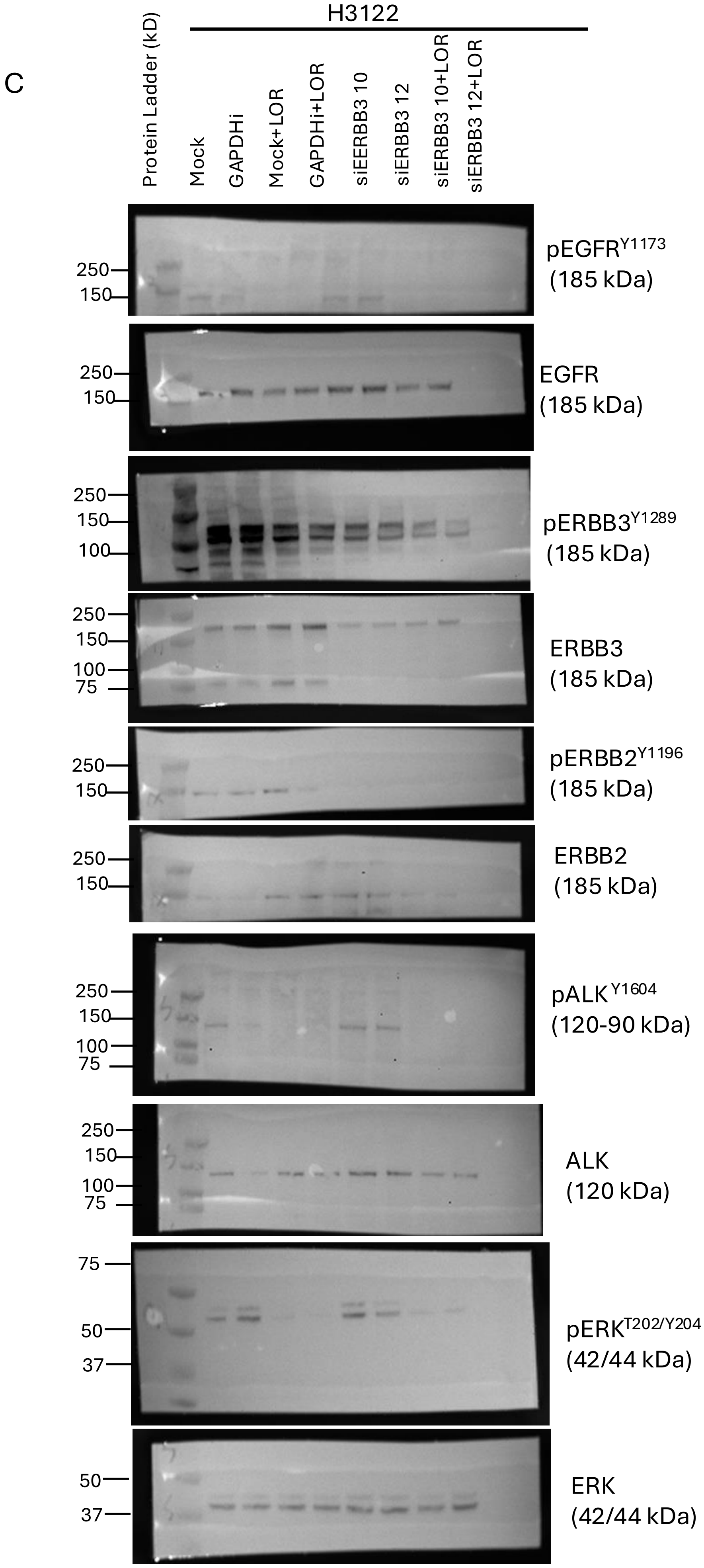
C
H3122
Protein Ladder (kD)
Mock
GAPDHi
Mock+LOR
GAPDHi+LOR
siEERBB3 10
siERBB3 12
siERBB3 10+LOR
siERBB3 12+LOR
pEGFR Y1173 (185 kDa)
EGFR (185 kDa)
pERBB3 Y1289 (185 kDa)
ERBB3 (185 kDa)
pERBB2 Y1196 (185 kDa)
ERBB2 (185 kDa)
pALK Y1604 (120-90 kDa)
ALK (120 kDa)
pERK T202/Y204 (42/44 kDa)
ERK (42/44 kDa)
250
150
100
75
50
37

Figure S8

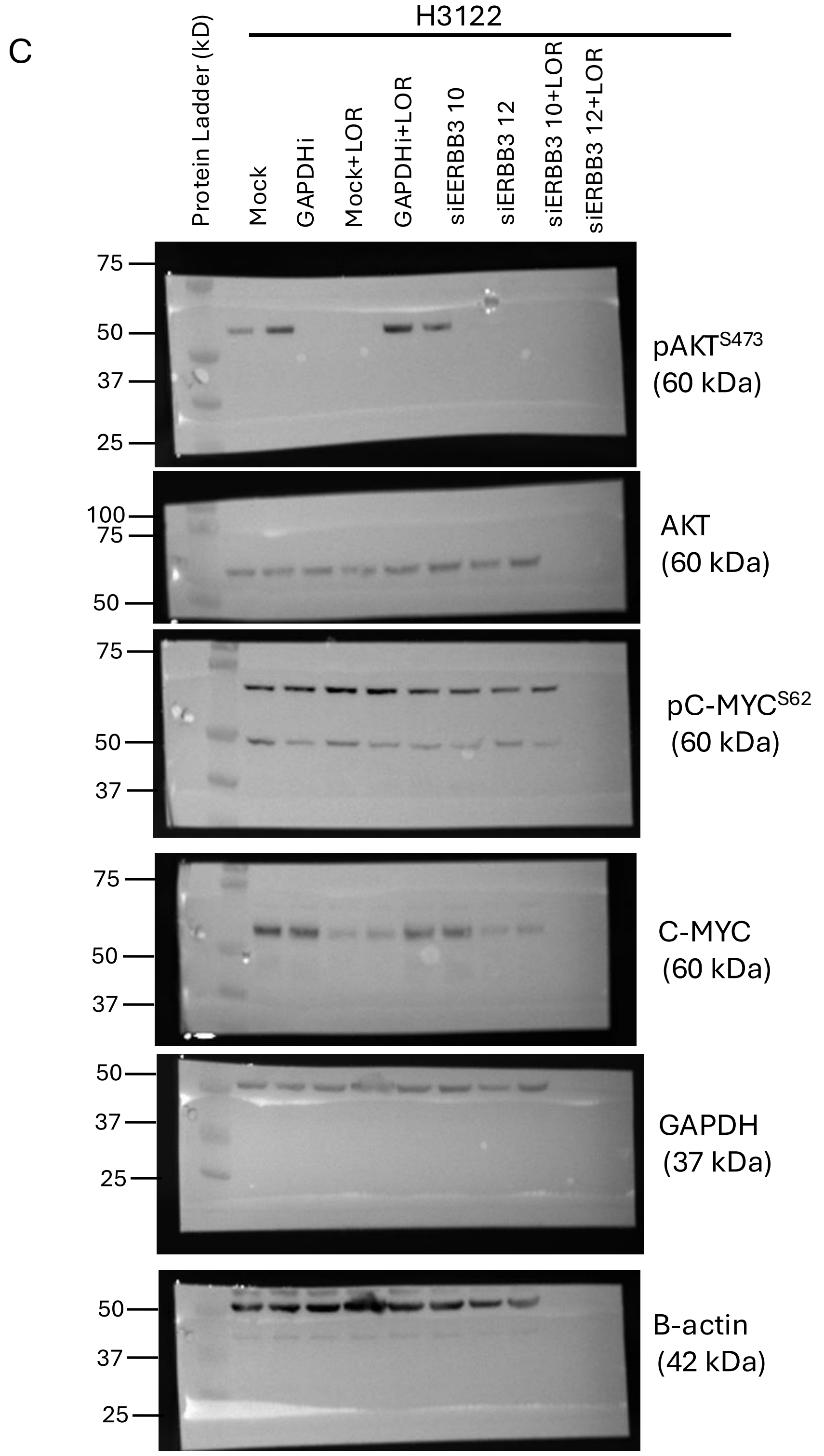
C
H3122
Protein Ladder (kD)
Mock
GAPDHi
Mock+LOR
GAPDHi+LOR
siEERBB3 10
siERBB3 12
siERBB3 10+LOR
siERBB3 12+LOR
75
50
37
25
pAKT S473
(60 kDa)
100
75
50
AKT
(60 kDa)
75
50
37
pC-MYC S62
(60 kDa)
75
50
37
C-MYC
(60 kDa)
50
37
25
GAPDH
(37 kDa)
50
37
25
B-actin
(42 kDa)

Figure S8

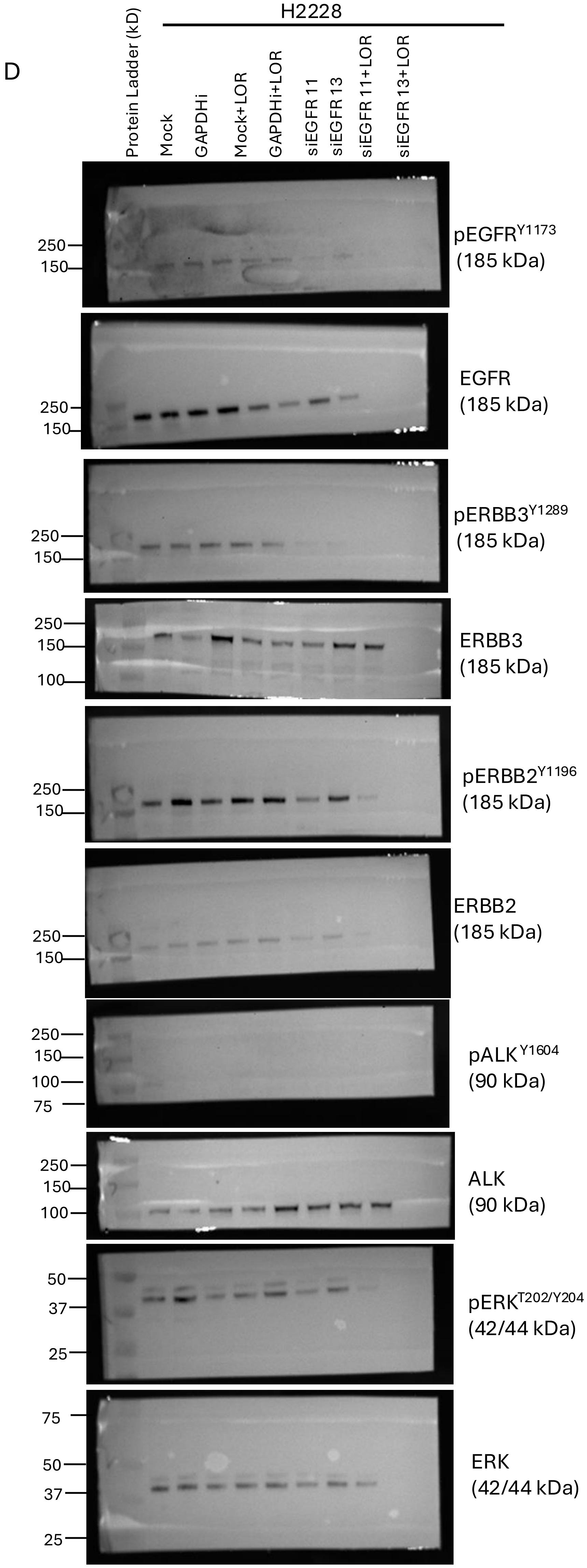
D
H2228
Protein Ladder (kD)
Mock
GAPDHi
Mock+LOR
GAPDHi+LOR
siEGFR11
siEGFR13
siEGFR11+LOR
siEGFR13+LOR
250
150
pEGFR Y1173 (185 kDa)
250
150
EGFR (185 kDa)
250
150
pERBB3 Y1289 (185 kDa)
250
150
100
ERBB3 (185 kDa)
250
150
pERBB2 Y1196 (185 kDa)
250
150
ERBB2 (185 kDa)
250
150
100
75
pALK Y1604 (90 kDa)
250
150
100
ALK (90 kDa)
50
37
25
pERK T202/Y204 (42/44 kDa)
75
50
37
25
ERK (42/44 kDa)

Figure S8

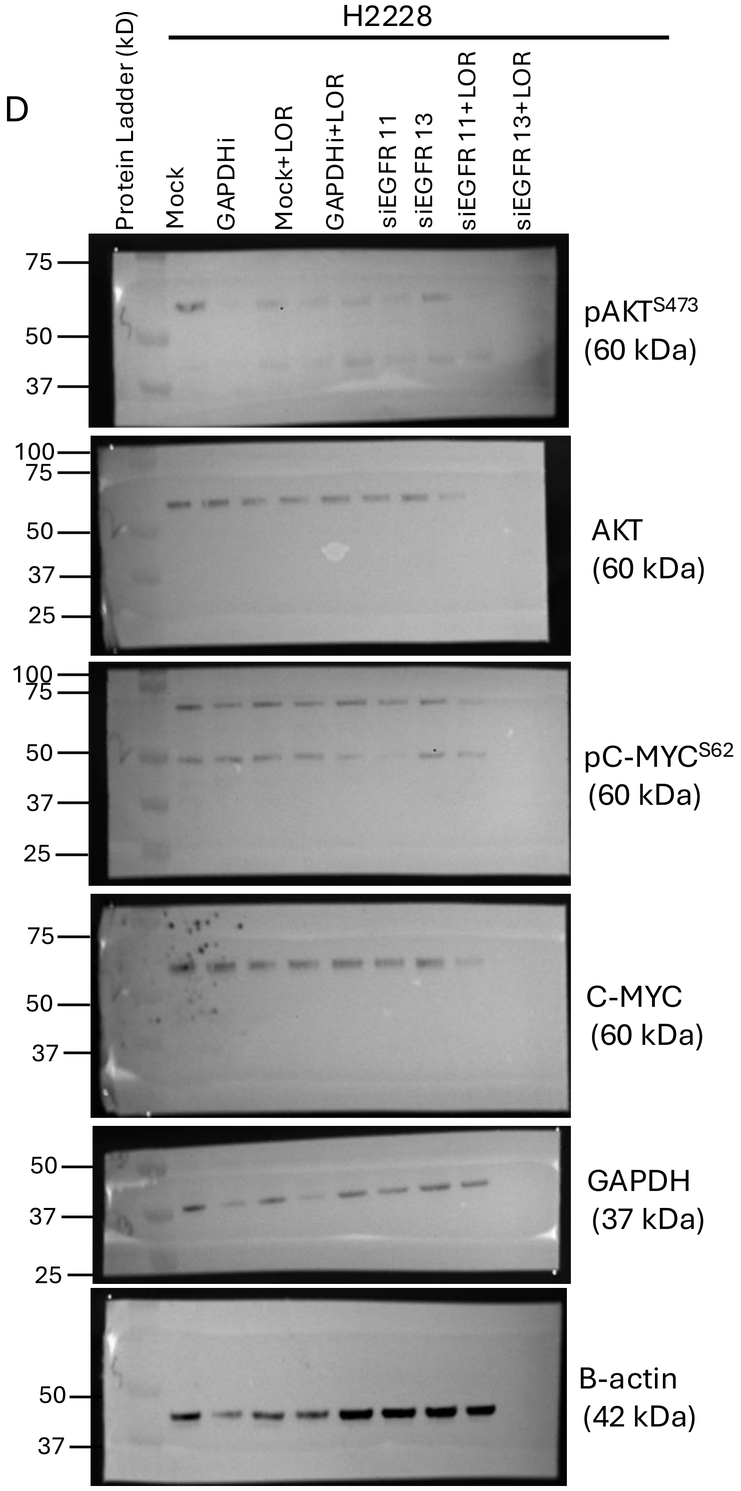

D
H2228
Protein Ladder (kD)
Mock
GAPDHi
Mock+LOR
GAPDHi+LOR
siEGFR11
siEGFR13
siEGFR11+LOR
siEGFR13+LOR
75
50
37
pAKT S473
(60 kDa)
100
75
50
37
25
AKT
(60 kDa)
100
75
50
37
25
pC-MYC S62
(60 kDa)
75
50
37
C-MYC
(60 kDa)
50
37
25
GAPDH
(37 kDa)
50
37
B-actin
(42 kDa)

Figure S8

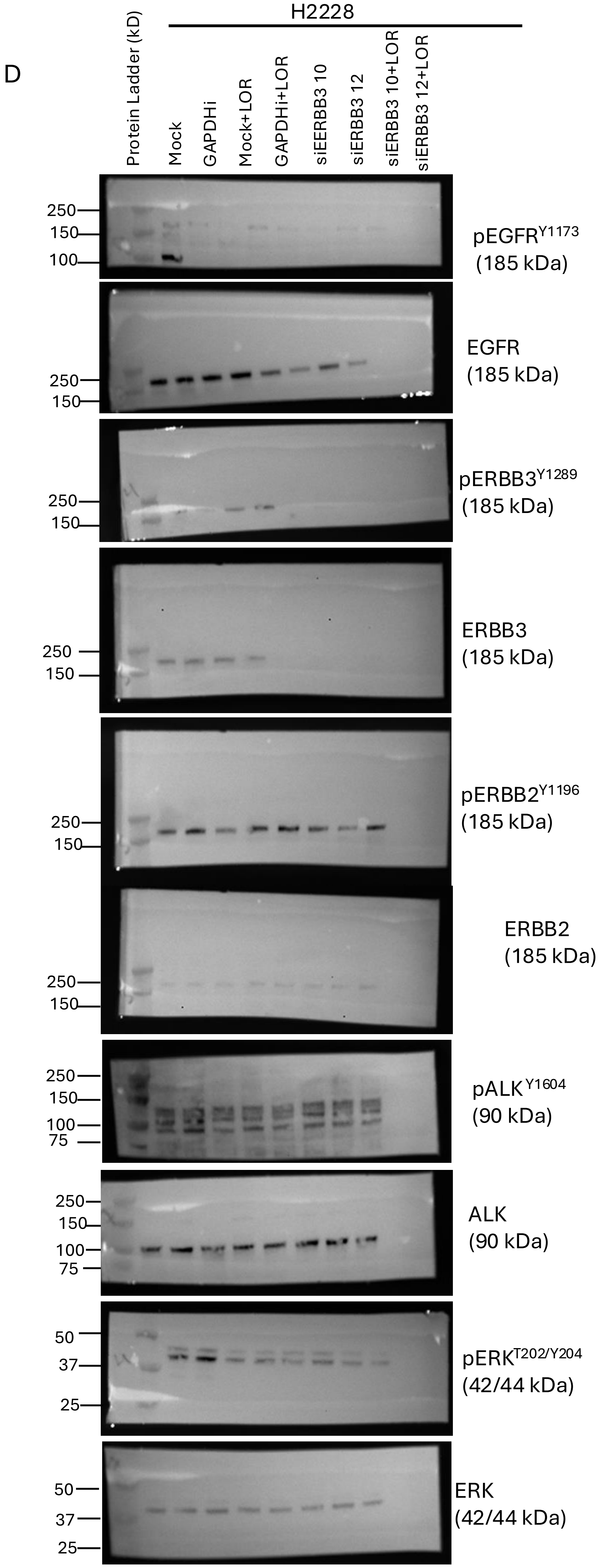
D
H2228
Protein Ladder (kD)
Mock
GAPDHi
Mock+LOR
GAPDHi+LOR
siEERBB3 10
siERBB3 12
siERBB3 10+LOR
siERBB3 12+LOR
pEGFR Y1173 (185 kDa)
EGFR (185 kDa)
pERBB3 Y1289 (185 kDa)
ERBB3 (185 kDa)
pERBB2 Y1196 (185 kDa)
ERBB2 (185 kDa)
pALK Y1604 (90 kDa)
ALK (90 kDa)
pERK T202/Y204 (42/44 kDa)
ERK (42/44 kDa)
250
150
100
75
50
37
25

Figure S8

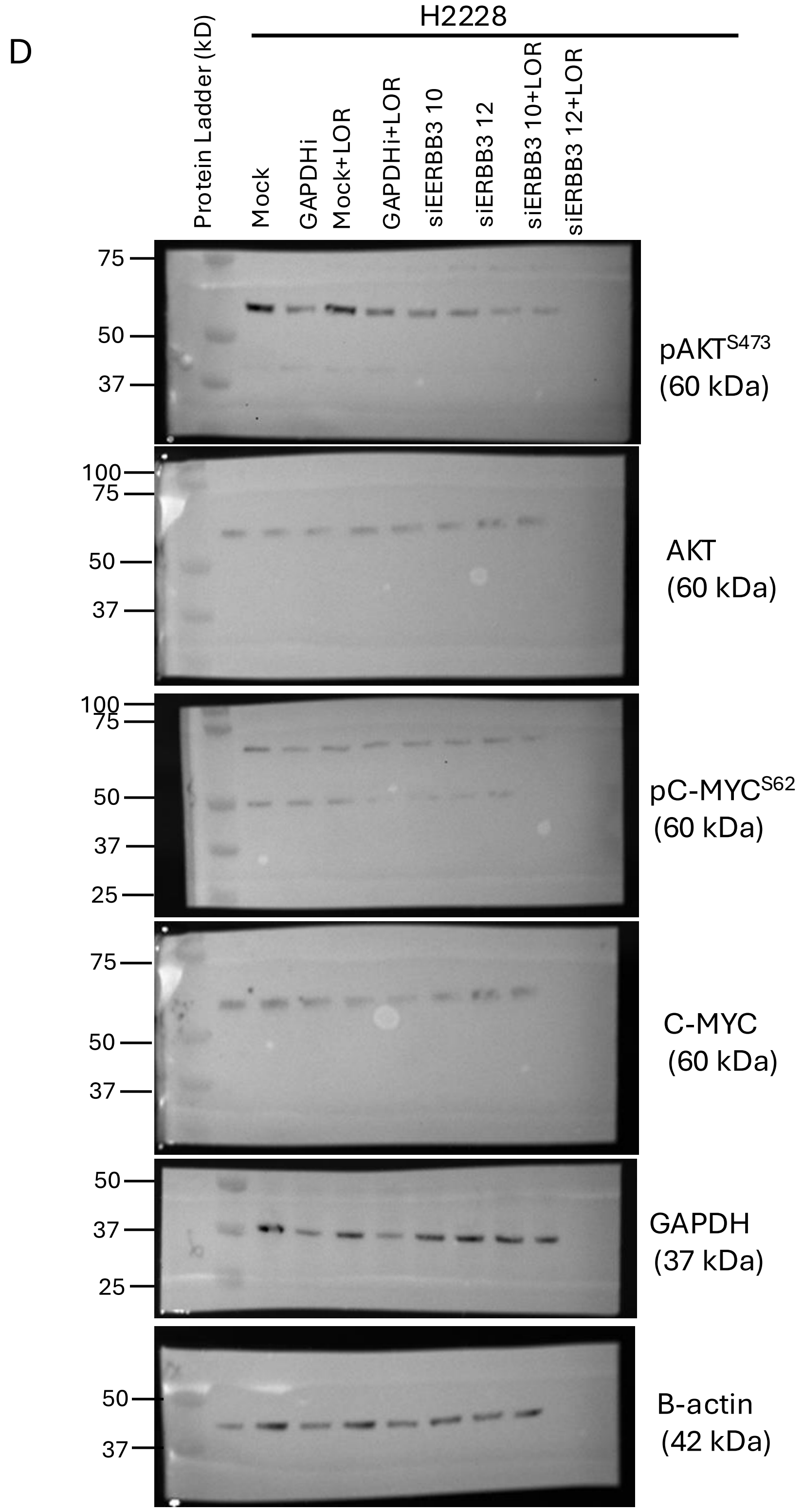
D
H2228
Protein Ladder (kD)
Mock
GAPDHi
Mock+LOR
GAPDHi+LOR
siEERBB3 10
siERBB3 12
siERBB3 10+LOR
siERBB3 12+LOR
75
50
37
pAKT^{S473}
(60 kDa)
100
75
50
37
AKT
(60 kDa)
100
75
50
37
25
pC-MYC^{S62}
(60 kDa)
75
50
37
C-MYC
(60 kDa)
50
37
25
GAPDH
(37 kDa)
50
37
B-actin
(42 kDa)

Figure S9

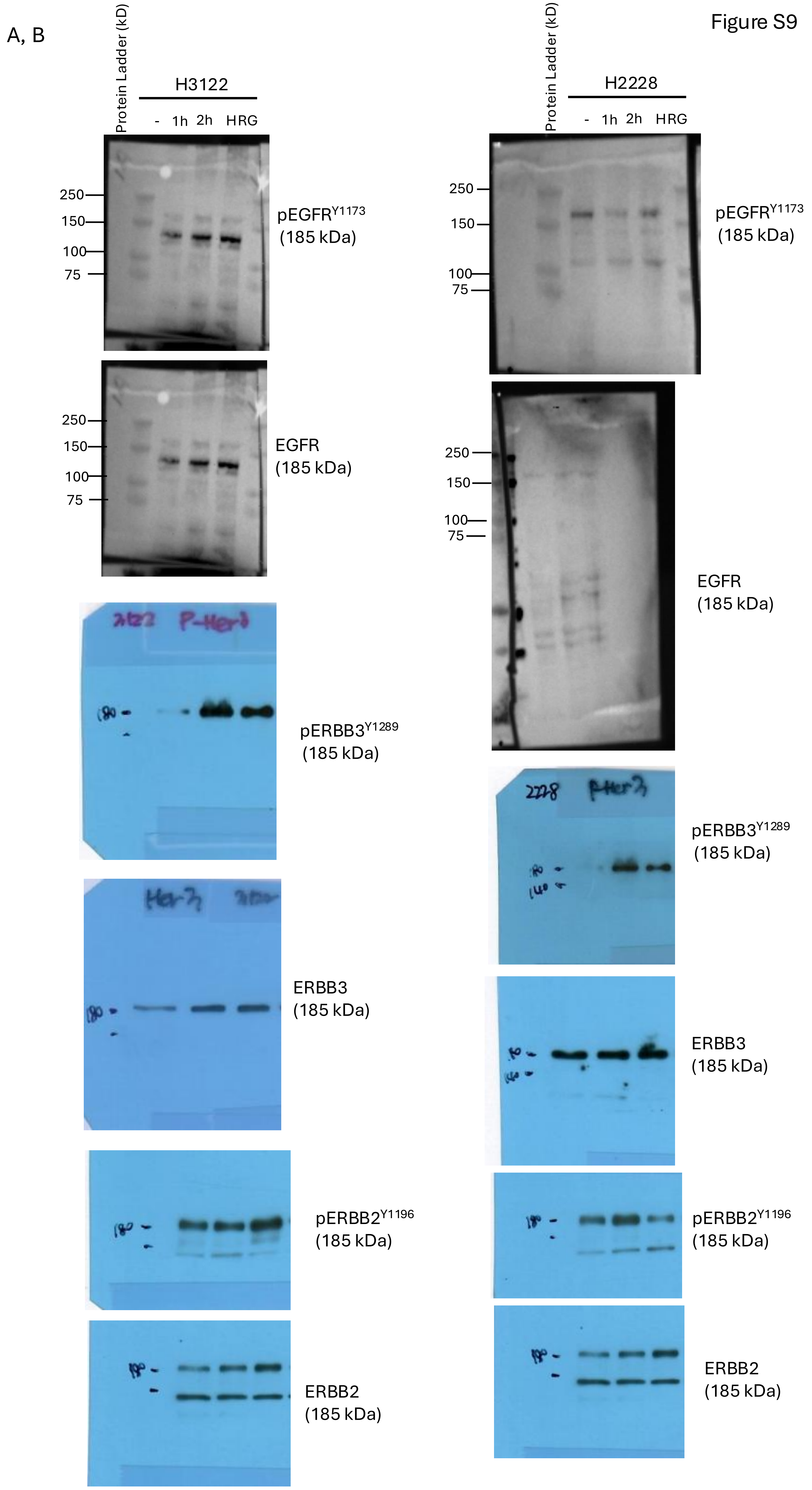
A, B
Protein Ladder (kD)
H3122
- 1h 2h HRG
250
150
100
75
pEGFR^Y1173
(185 kDa)
EGFR
(185 kDa)
pERBB3^Y1289
(185 kDa)
ERBB3
(185 kDa)
pERBB2^Y1196
(185 kDa)
ERBB2
(185 kDa)
Protein Ladder (kD)
H2228
- 1h 2h HRG
250
150
100
75
pEGFR^Y1173
(185 kDa)
EGFR
(185 kDa)
pERBB3^Y1289
(185 kDa)
ERBB3
(185 kDa)
pERBB2^Y1196
(185 kDa)
ERBB2
(185 kDa)

Figure S9

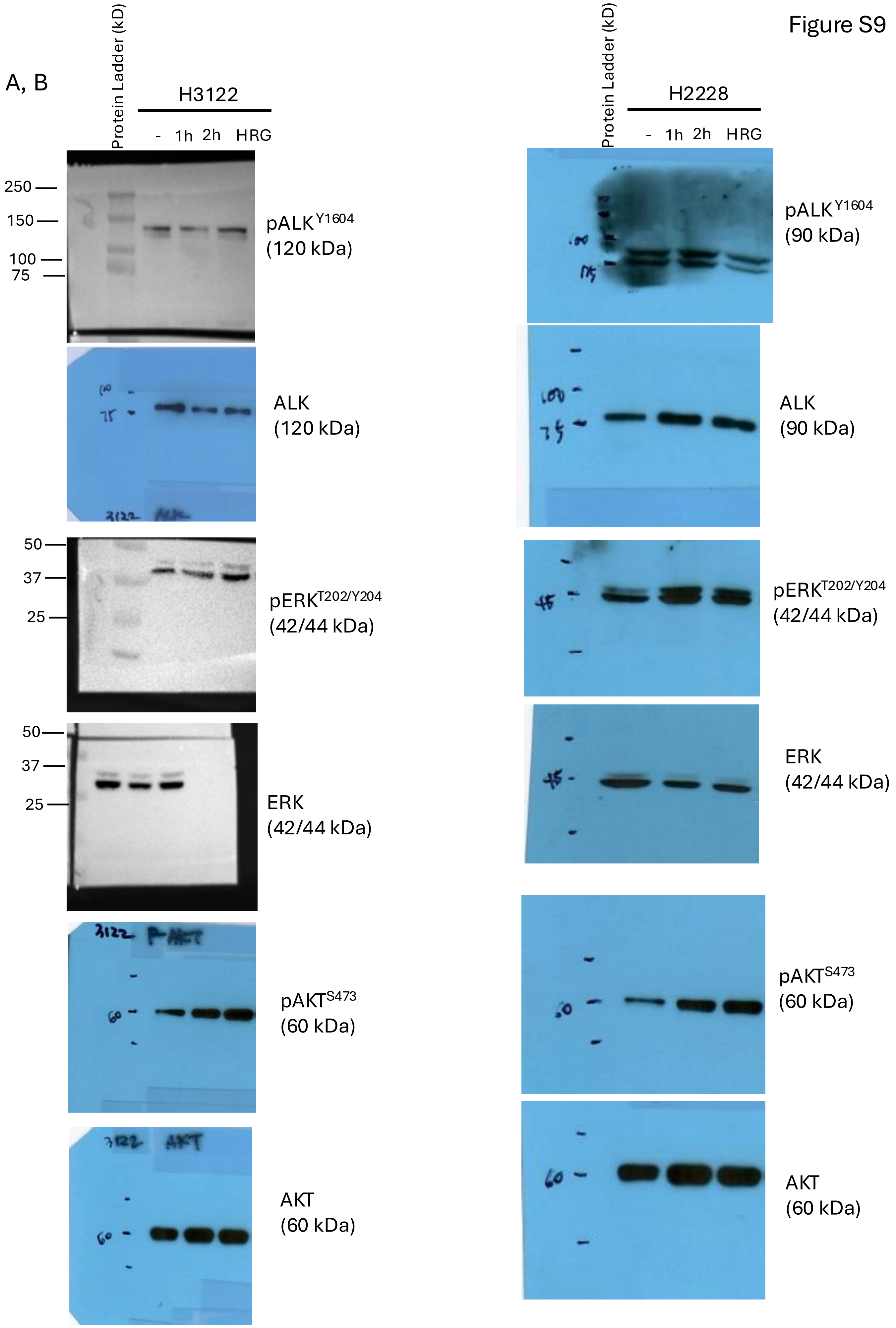
A, B
Protein Ladder (kD)
H3122
- 1h 2h HRG
250
150
100
75
pALK Y1604
(120 kDa)
ALK
(120 kDa)
50
37
25
pERK T202/Y204
(42/44 kDa)
50
37
25
ERK
(42/44 kDa)
pAKT S473
(60 kDa)
AKT
(60 kDa)
Protein Ladder (kD)
H2228
- 1h 2h HRG
pALK Y1604
(90 kDa)
ALK
(90 kDa)
pERK T202/Y204
(42/44 kDa)
ERK
(42/44 kDa)
pAKT S473
(60 kDa)
AKT
(60 kDa)

Figure S9

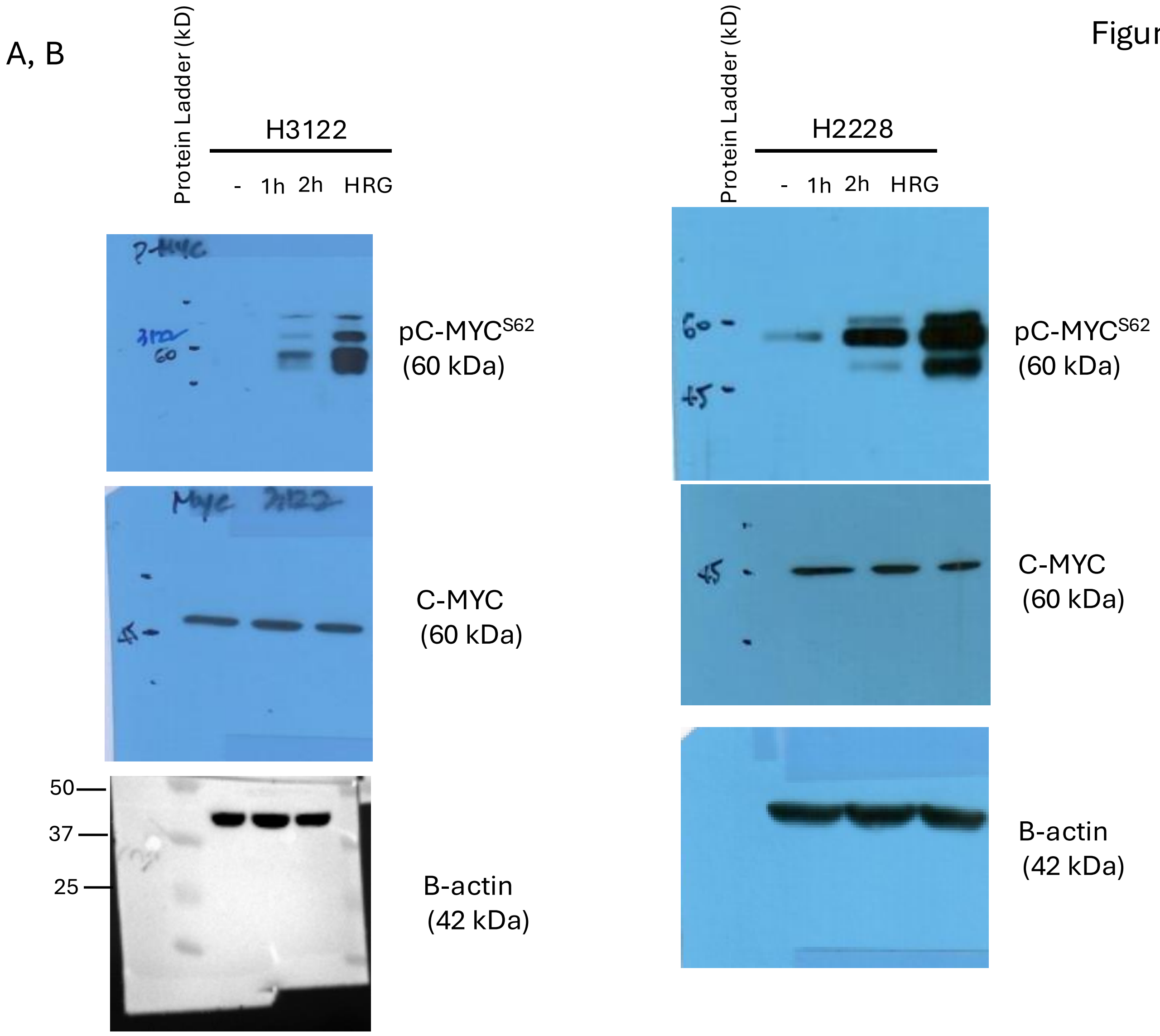
A, B
Protein Ladder (kD)
H3122
- 1h 2h HRG
pC-MYC^S62
(60 kDa)
C-MYC
(60 kDa)
50
37
25
B-actin
(42 kDa)
Protein Ladder (kD)
H2228
- 1h 2h HRG
pC-MYC^S62
(60 kDa)
C-MYC
(60 kDa)
B-actin
(42 kDa)

Figure S11

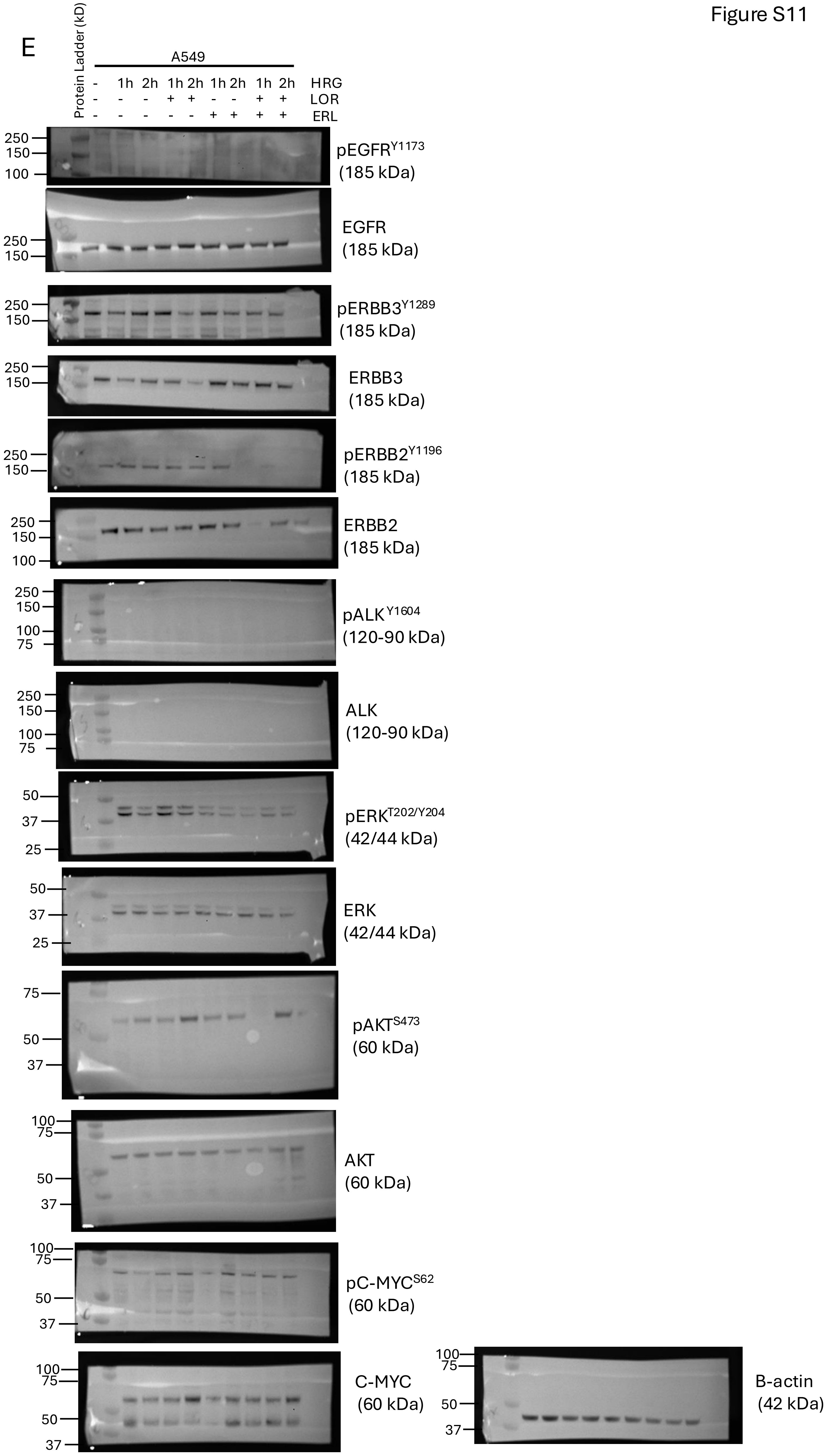
E
Protein Ladder (kD)
A549
- 1h 2h 1h 2h 1h 2h 1h 2h HRG
- - - + + - - + + LOR
- - - - - + + + + ERL
250
150
100
pEGFR Y1173
(185 kDa)
250
150
EGFR
(185 kDa)
250
150
pERBB3 Y1289
(185 kDa)
250
150
ERBB3
(185 kDa)
250
150
pERBB2 Y1196
(185 kDa)
250
150
100
ERBB2
(185 kDa)
250
150
100
75
pALK Y1604
(120-90 kDa)
250
150
100
75
ALK
(120-90 kDa)
50
37
25
pERK T202/Y204
(42/44 kDa)
50
37
25
ERK
(42/44 kDa)
75
50
37
pAKT S473
(60 kDa)
100
75
50
37
AKT
(60 kDa)
100
75
50
37
pC-MYC S62
(60 kDa)
100
75
50
37
C-MYC
(60 kDa)
100
75
50
37
B-actin
(42 kDa)

Figure S11

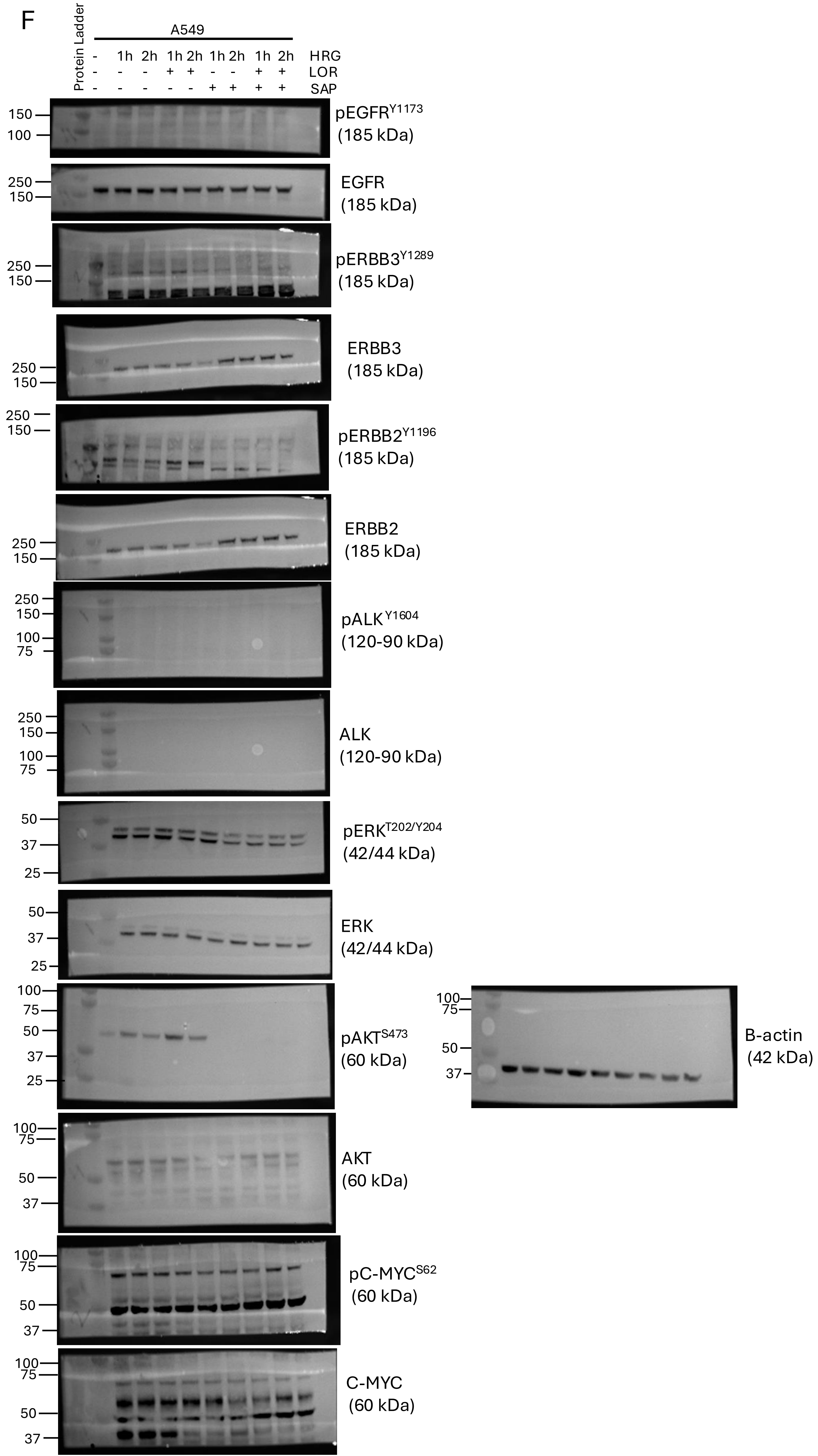

F
Protein Ladder (kD)
A549
- 1h 2h 1h 2h 1h 2h 1h 2h HRG
- - - + + - - + + LOR
- - - - - + + + + SAP
pEGFR^{Y1173} (185 kDa)
EGFR (185 kDa)
pERBB3^{Y1289} (185 kDa)
ERBB3 (185 kDa)
pERBB2^{Y1196} (185 kDa)
ERBB2 (185 kDa)
pALK^{Y1604} (120-90 kDa)
ALK (120-90 kDa)
pERK$^{T202/Y204}$ (42/44 kDa)
ERK (42/44 kDa)
pAKT^{S473} (60 kDa)
AKT (60 kDa)
pC-MYC^{S62} (60 kDa)
C-MYC (60 kDa)
B-actin (42 kDa)
250
150
100
75
50
37
25

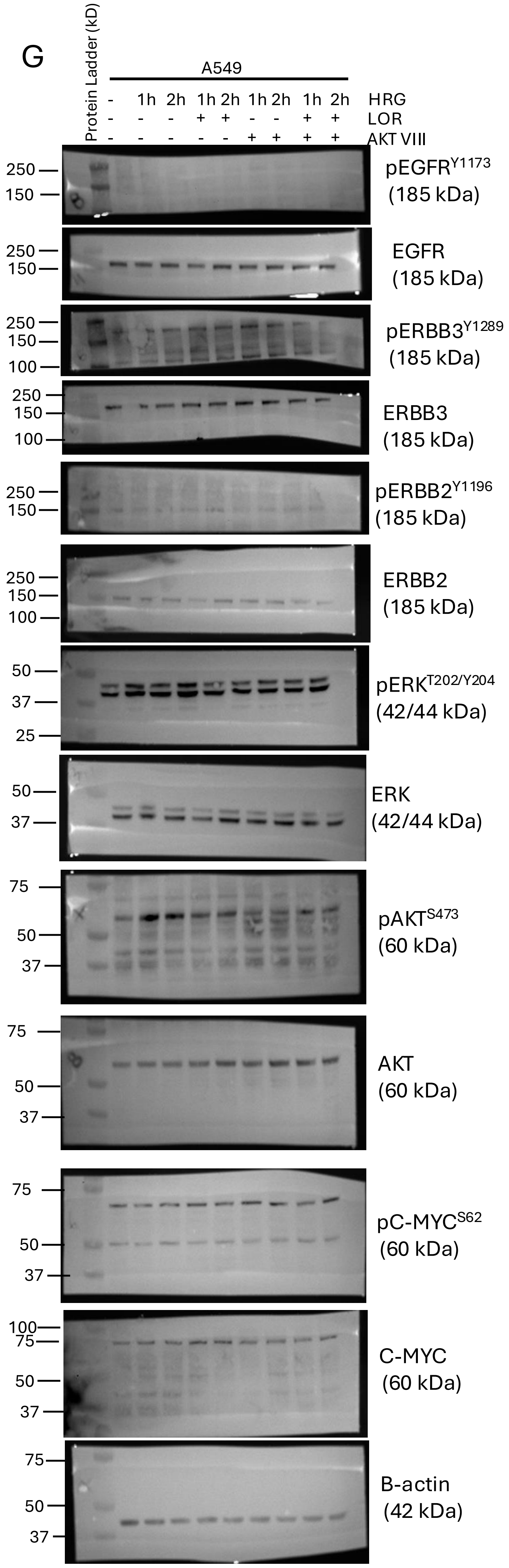
G
Protein Ladder (kD)
A549
- 1h 2h 1h 2h 1h 2h 1h 2h HRG
- - - + + - - + + LOR
- - - - - + + + + AKT VIII
250
150
pEGFR^Y1173 (185 kDa)
EGFR (185 kDa)
pERBB3^Y1289 (185 kDa)
100
ERBB3 (185 kDa)
pERBB2^Y1196 (185 kDa)
ERBB2 (185 kDa)
50
37
25
pERK^T202/Y204 (42/44 kDa)
ERK (42/44 kDa)
75
pAKT^S473 (60 kDa)
AKT (60 kDa)
pC-MYC^S62 (60 kDa)
C-MYC (60 kDa)
B-actin (42 kDa)
